# Plant population responses to environmental variability are primarily driven by survival-reproduction trade-offs and mediated by aridity

**DOI:** 10.1038/s41467-026-73720-x

**Published:** 2026-05-28

**Authors:** Gabriel Silva Santos, Xianyu Yang, Samuel J. L. Gascoigne, Aldo Compagnoni, André T. C. Dias, Shripad Tuljapurkar, Maja Kajin, Roberto Salguero-Gómez

**Affiliations:** 1 National Institute of the Atlantic Forest (INMA), Santa Teresa, Brazil; 2 Department of Ecology, Graduate Program in Ecology and Evolution, Rio de Janeiro State University, Maracanã, Brazil; 3 Department of Biology, University of Oxford, Oxford, UK; 4 Institute of Integrative Biology, ETH Zürich, Zürich, Switzerland; 5 WSL Swiss Federal Institute for Forest, Snow and Landscape Research, Birmensdorf, Switzerland; 6 School of Biological Sciences, University of Aberdeen, Aberdeen, UK; 7 iDiv. Puschstrasse 4, Leipzig, Germany; 8 Institute of Biology Martin Luther University Halle, Halle, Germany; 9 Department of Ecology, Institute of Biology, Universidade Federal do Rio de Janeiro, Rio de Janeiro, Brazil; 10 Department of Biology, Stanford University, Stanford, CA USA; 11 Department of Biology, Biotechnical Faculty, University of Ljubljana, Ljubljana, Slovenia

**Keywords:** Biodiversity, Evolution, Environmental impact

## Abstract

Natural populations may suffer negatively from increased environmental variability due to climate change; however, several mechanisms can mitigate those effects by changing the vital rates of a population (*e.g*., survival, reproduction). Despite important analytical and theoretical advances, we still do not know how and to what extent environmental regimes, life history traits, and evolutionary history determine the buffering capacity of natural populations. To address these questions, we parameterise a Bayesian generalised linear mixed model with high-resolution vital rate data from 121 natural populations across 78 plant species. We show that population responses to environmental variability vary four orders of magnitude along a ‘demographic buffering continuum’. Furthermore, the position of a given population along said continuum is predicted by a survival*-*reproduction trade-off and by the degree of aridity the population experiences. Our findings open a promising avenue of research to improve ecological forecasts and management of natural populations in the Anthropocene.

## Abstract (web summary)

To understand how life-history strategies mitigate demographic impacts of climate vulnerability, authors map plant population responses along a demographic axis and reveal survival-reproduction trade-offs and aridity govern these responses.

## Introduction

Climate change projections anticipate a global increase in not only the mean of key abiotic drivers (*e.g*., temperature, precipitation), but also their temporal variability^1^. This variability encompasses both deterministic patterns (e.g., seasonality, long-term directional trends) and environmental stochasticity—the random, unpredictable fluctuations around those patterns^2^. Together, these sources of variation shape the environmental context in which populations persist and evolve, and both can strongly influence long-term population viability^3,4^. Consequently, climate change has already introduced significant eco-evolutionary challenges to the performance and survival of natural populations^4,5^. Indeed, climate change has been identified as a major driver of biodiversity loss worldwide^6^. As such, understanding whether and how the expected negative effects of environmental variability can be minimised has become a primary mission of Ecology, Evolution, and Conservation Biology.

Half a century of research examining how species’ life history strategies are shaped by natural selection to cope with environmental variability has led to the establishment of two major axes of life history variation: the fast-slow continuum^7,8^ and the reproductive strategies continuum^9–11^. The fast-slow continuum ranks organisms according to a development-survival trade-off from fast-growing, short-lived organisms to slow-growing, long-lived ones^7^. In contrast, the reproductive strategy continuum, which is complementary to the fast-slow continuum^12–14^, categorises organisms based on the length of their reproductive window, from single (i.e., semelparous) to multiple reproductive bouts (*i.e*., iteroparous)^9,15^. Recent advances in biodemography have linked species at the slow-end of the fast-slow continuum to a greater capacity to buffer the negative impacts of environmental variation mediated by natural selection^16–18^. This so-called demographic buffering^16,17^ is accomplished by reducing the temporal variance of those vital rates (e.g., survival, growth, reproduction) that contribute most to stochastic population growth rate (*λ*_*s*_).

So far, the link between life history continua and demographic buffering remains largely untapped for multiple reasons. First, we still lack a general understanding of how each vital rate drives populations’ response to environmental variability. Second, the demographic buffering hypothesis^16,17^ often assumes linear or concave (∩-shaped) environmental-vital rate responses^19–21^, which indeed tends to result in negative effects on long-term of stochastic population growth (*λ*_*s*_) when environmental variation increases. Alternatively, some vital rates, particularly growth or reproduction, may be convex (∪-shape; e.g., exponential response)^20,22^, showing disproportionately high values under favourable conditions, which then offset losses in bad years^23^. In these alternative cases, the resulting stochastic population growth rate, *λ*_*s*_, may exceed the deterministic population growth rate, *λ*, obtained from the mean environment. This outcome, while theoretically possible, requires (1) a substantial contribution from the vital rate to *λ* and (2) environmental variability that stays within a range where its response remains convex. The likelihood of both conditions occurring in nature is debatable^24,25^. Third, and equally important, environmental variability has, to date, not been explicitly considered in studies of demographic buffering. Consequently, populations exposed to reduced temporal variability in climate variables during the study time frame are prone to being interpreted as buffered, regardless of whether they have been exposed to high or low environmental variability. Altogether, these complementary parts of the demographic buffering hypothesis remain a major challenge in linking life history continua to populations’ response to environmental variability in the context of climate change.

Here, we examine how environmental regimes, key life history traits, and evolutionary history predict the extent to which natural populations of plants can buffer against environmental variability along a demographic buffering continuum. Specifically, we parameterise a phylogenetically-corrected Bayesian Generalised Linear Mixed Model (GLMM) with high-resolution vital rate data from 121 natural populations of 78 plant species to test the following hypothesis: (*H*_*1*_) the position of plant species’ populations along the demographic buffering continuum is only weakly predicted by the fast-slow and reproductive strategies continua. Although a study using 36 populations of different species found that longevity (a life history trait aligned with the fast-slow continuum^10,11,14^) is a good proxy of demographic buffering^18^, a more recent study with a greater number of populations and accounting for their evolutionary history provided opposing evidence^26^. Moreover, because each vital rate responds differently to environmental variability^27^, it is challenging to anticipate a general pattern of temporal variation across all vital rates, thus blurring our current understanding of buffering in organisms. To better understand the contribution of vital rates on demographic buffering, we test (*H*_*2*_) whether the phylogenetic signal of the buffering capacities of the examined species is stronger in the vital rates that contribute most to *λ*_s_, since demographic buffering is a result of natural selection^16,28^; Finally, none of the existing studies exploring demographic buffering have explicitly considered the variability of the environmental faced by the studied population. This omission likely inflates the perceived roles of life history traits and phylogenetic relationships in explaining population responses to environmental variability, which leads us to hypothesise (*H*_*3*_) that the extent to which a natural population displays demographic buffering strongly depends on its environmental regime. Specifically, higher variability in precipitation and temperature is expected to lead to greater variances in vital rates, shifting populations toward the less buffered end of the demographic buffering continuum.

To test our hypotheses, we first establish the existence of a continuum of vital rate variance from high to low demographic buffering. This continuum emerges when examining how the observed temporal variance of vital rates has shaped the long-term population performance of each natural population. To do so, we use the sum of stochastic elasticity of the stochastic population growth rate ${\lambda }_{s}$ with respect to vital rate temporal variance, $\sum {E}_{v}^{\sigma }$. This variable was calculated from a subset of matrix population models from COMPADRE^29^ database that depict the demography of populations under natural environmental conditions (see Methods for further details on the data selection criteria). Values of $\sum {E}_{v}^{\sigma }$ close to 0 correspond with small effects of the temporal variation of vital rates on population growth rate ${\lambda }_{s}$ (i.e., high demographic buffering), while absolute $\left|\sum {E}_{v}^{\sigma }\right|$ values greater than 0 represent a more tangible effect on ${\lambda }_{s}$ (low demographic buffering)^30^. Next, we also quantify the responses of 121 natural populations to environmental regime in the context of their positions along multiple axes of variation: the fast-slow and reproductive continua, and environmental variability, using Principal Component Analyses (PCAs).

## Results

**Fig. 1:**
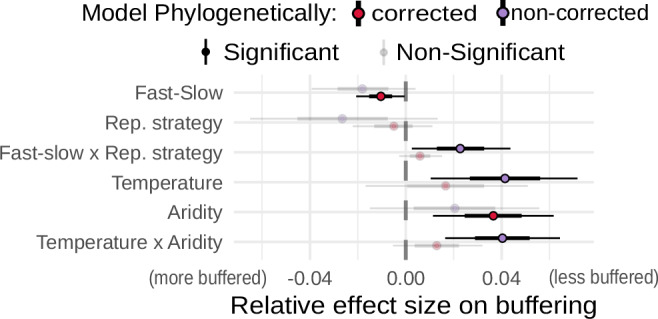
The position of our 121 plant populations along the buffering continuum – as quantified by the absolute value of the sum of stochastic elasticities of population growth rate to changes in the variance of all vital rates (*v*), $\left|\sum {E}_{v}^{\sigma }\right|$ – is mainly determined by variability in aridity. The figure shows the effect sizes (posterior distributions) of the studied variables on $\left|\sum {E}_{v}^{\sigma }\right|$, which were estimated via MCMCglmm models with (red) and without (purple) phylogenetic corrections. Points and bars represent the posterior medians and 95% credible intervals, respectively. Negative effect sizes indicate variables that promote demographic buffering. The models show that the contributions of reproductive strategies and temperature are only significant when phylogenetic relationships are not accounted for. The life-history axes are represented by ${PC}{1}_{{LH}}$ (fast–slow continuum) and ${PC}{2}_{{LH}}$ (reproductive-strategy continuum; see Fig. S2), while environmental variability is summarised by ${PC}{1}_{{Env}}$ (gradient in mean and stochasticity in temperature) and ${PC}{2}_{{Env}}$ (variability in aridity; see Fig. S4).

The 121 plant populations examined unveil a demographic buffering continuum that spans four orders of magnitude, as quantified by $\left|\sum {E}_{v}^{\sigma }\right|$, with the lower value $\left|\sum {E}_{v}^{\sigma }\right|$ = 0.000 ± 0.005 for the Pyrenean violet (*Ramonda myconi*) and maximum value of $\left|\sum {E}_{v}^{\sigma }\right|$ = 2.014 ± 3.420 for the Mexican hat (*Ratibida columnifera*). Based on the most plausible Bayesian Generalised Linear Mixed Model (GLMM) reached, plant populations’ capacity to buffer environmental fluctuations is significantly linked to their position on the life-history continua, but only if environmental variability and evolutionary history are not accounted for. Indeed, this result supports our hypothesis *H*_*1*_, which is the importance of fast-slow and reproductive strategies in positioning plant population along the demographic buffering continuum, measured as $\left|\sum {E}_{v}^{\sigma }\right|$ (the cumulative effect of variation), decreases when evolutionary history and environmental variability are accounted for (Fig. 1; Table S3). For instance, considering our model without phylogenetic corrections, the interaction between the fast-slow and reproductive strategy continua (the first two principal component axes of life histories, ${PC}{1}_{{LH}}\,:{PC}{2}_{{LH}}$, respectively) determines the position of populations along the demographic buffering continuum (non-phylogenetic corrected MCMCglmm $|\sum {E}_{v}^{\sigma }|$, ${\beta }_{{PC}{1}_{{LH}}\,:{PC}{2}_{{LH}}}\,$= 0.022 [0.002 − 0.044]; posterior mean and 95% credible interval). However, after controlling for phylogenetic relationships in our data, only the fast-slow continuum (${PC}{1}_{{LH}}$) remains an important predictor of $|\sum {E}_{v}^{\sigma }|$ (phylogenetically corrected MCMCglmm $|\sum {E}_{v}^{\sigma }|$, ${\beta \lambda }_{{PC}{1}_{{LH}}\,}\,$= −0.011 [−0.022 − 0.000]; Fig. 1; Table S3). These aforementioned discrepancies between phylogenetically and non-phylogenetically corrected models support the idea that evolutionary history plays an important role in the placement of populations along the demographic buffering continuum.

**Fig. 2:**
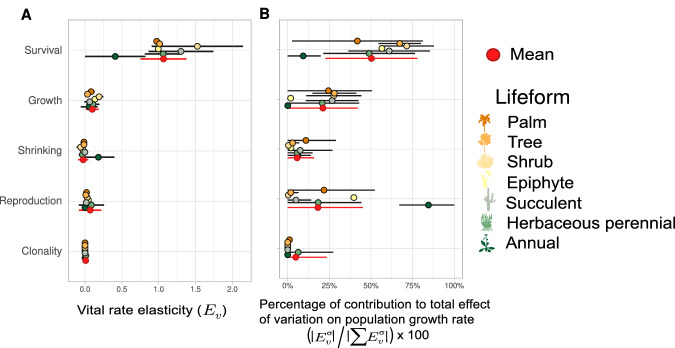
Variance in survival is the primary driver of population responses to environmental variability in most of our 121 plant populations, followed by individual-level growth and reproduction. **A** The proportional contribution of each vital rate (survival, growth, shrinkage, reproduction, clonality) to stochastic population growth rate ${\lambda }_{s}$ quantified from the stochastic elasticities (${E}_{v}$). **B** The proportional contribution of each vital rate to the populations’ positions along the buffering continuum, as quantified by ${E}_{v}^{\sigma }$. Note that panel B is scaled to show proportional contributions as percentages, calculated as $\left[\frac{|{E}_{v}^{\sigma }|}{\sum {{|E}}_{v}^{\sigma }|}\right]\times 100$. Different life forms of plants are represented by different colours. Points and bars represent the mean and standard deviation, respectively. Life-form silhouettes and their respective credits are available at https://www.phylopic.org/permalinks/08530f888922c9aa86e65b93ec833826377ac7daad224fdf814c1abd7a6a7e04.

**Fig. 3:**
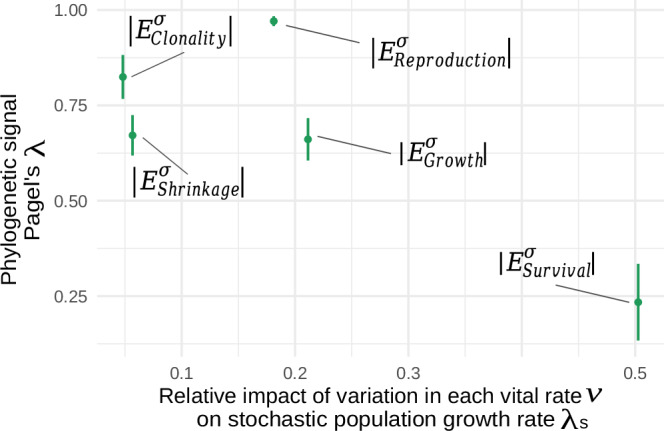
The vital rates that most drive buffering in plant populations are not necessarily those whose contributions are best predicted by evolutionary history. The figure shows the absolute contribution of each vital rate *v* to 121 plant populations’ positions along the buffering continuum, ${{|E}}_{v}^{\sigma }|$, and its phylogenetic signal *(*Pagel’s *λ*). Points and bars represent the mean and standard deviation, respectively. Here, Pagel’s *λ* quantifies how evolutionary history explains the contribution of each vital rate to demographic buffering. A phylogenetic signal closer to 1 indicates higher predictability by phylogeny. Contrary to our hypothesis (*H₂*), the vital rates whose temporal variation contributed most to ${\lambda }_{s}$ (i.e., ${E}_{{Survival}}^{\sigma }$) did not show a stronger phylogenetic signal.

**Fig. 4:**
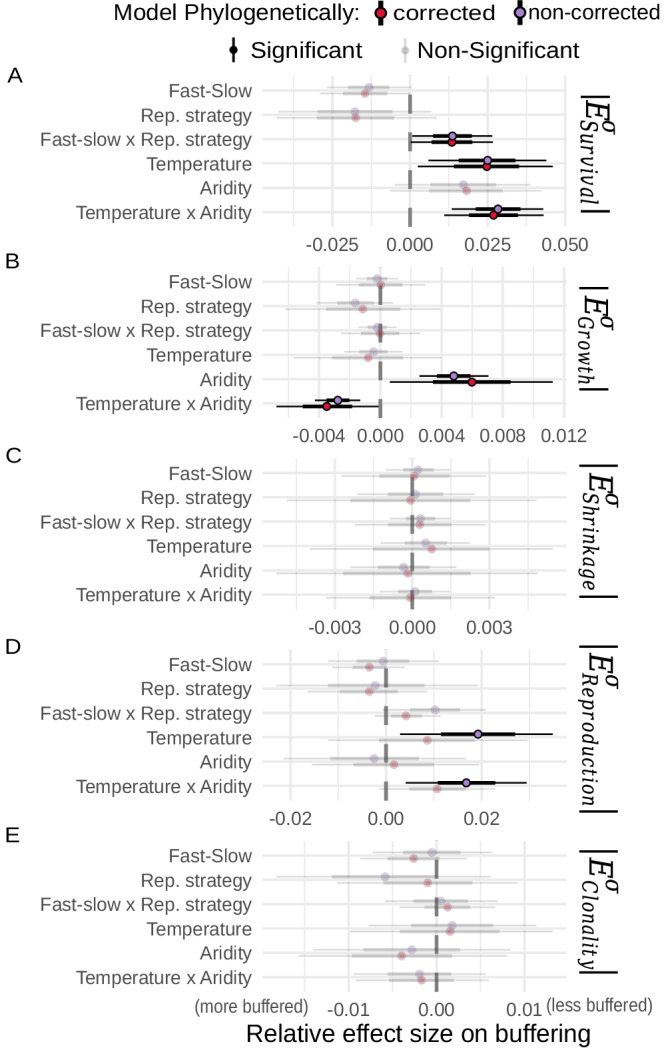
The effects of the contributions of life history strategies and environmental variability to the position of our examined 121 plant populations along the buffering continuum vary greatly across vital rates ($|{E}_{v}^{\sigma }|$; *e.g*., $|{E}_{{Survival}}^{\sigma }|$, $|{E}_{{Clonality}}^{\sigma }|$). MCMCglmm models with (red) and without (purple) phylogenetic corrections are presented. Each panel represents a separate model for a specific vital rate: **A** Survival, **B** Growth, **C** Shrinking, **D** Reproduction, and **E** Clonality. Points and bars represent the posterior medians and 95% credible intervals, respectively. The life-history axes are represented by ${PC}{1}_{{LH}}$ (fast–slow continuum) and ${PC}{2}_{{LH}}$ (reproductive-strategy continuum; see Fig. S2), while environmental variability is summarised by ${PC}{1}_{{Env}}$ (gradient in mean and stochasticity in temperature) and ${PC}{2}_{{Env}}$ (variation in aridity; see Fig. S4).

It is important to consider that each vital rate contributes differently to demographic buffering and presents a different degree of phylogenetic conservatism. Indeed, the position of populations along the demographic buffering continuum is mainly determined by the temporal variance of survival (${E}_{{Survival}}^{\sigma }$ = 50.26 ± 27.60%; Fig. 2b), followed by individual-level growth (${E}_{{Growth}}^{\sigma }$ = 21.13 ± 21.01%) and reproduction (${E}_{{Reproduction}}^{\sigma }$ = 18.12 ± 27.05%), with minor contributions from individual-level shrinkage (${E}_{{Shrinkage}}^{\sigma }$ = 5.67 ± 10.12 %) and clonality (${E}_{{Clonality}}^{\sigma }$ = 4.81 ± 18.57 %; Fig. 2b and Table S4). However, contrary to our expectations (*H*_*2*_), we did not find a positive relationship between the vital rates that contribute most to this buffering capacity and their phylogenetic signal (r = −0.7, S = 34, p-value = 0.230). Indeed, survival, the vital rate that contributes most to buffering, shows the weakest phylogenetic signal (Pagel’s $\lambda$ of ${E}_{{Survival}}^{\sigma }$ = 0.240; Fig. 3). On the other hand, the contributions of temporal variation in sexual reproduction and clonality to ${\lambda }_{s}$ (${E}_{{Reproduction}}^{\sigma }$ and ${E}_{{Clonality}}^{\sigma }$) are highly phylogenetically conserved, as revealed by the strongest phylogenetic signal (Pagel’s $\lambda$ of ${E}_{{Reproduction}}^{\sigma }\,$= 0.972 and Pagel’s $\lambda$ of ${E}_{{Clonality}}^{\sigma }$ = 0.823, respectively; Fig. 3). This high phylogenetic signal is also evident in the GLMM models, particularly for reproduction, where the contribution of the fast-slow continuum (${PC}{1}_{{LH}}$) on determining ${E}_{{Reproduction}}^{\sigma }$ disappears when phylogenetic relationships are accounted for in our analyses (see Fig. 4d).

Finally, our phylogenetically and non-phylogenetically corrected models both support *H*_*3*_, that populations located in more variable environments are positioned towards the lower-buffering end $(\left|\sum {E}_{v}^{\sigma }\right|\gg 0)$ of the demographic buffering continuum. Indeed, in our 121 natural populations of plants, temporal variation in aridity ($\left|\sum {E}_{v}^{\sigma }\right|,\,{\beta \lambda }_{{PC}{2}_{{Env}}\,}\,$= 0.036 [0.008 to 0.060]), more so than in temperature (${\beta \lambda }_{{PC}{1}_{{Env}}\,}\,$= 0.017 [−0.017 to 0.052]; Fig. 1), act as the main determinant of the placement of populations along the demographic buffering continuum. Moreover, the effect of temporal variation in aridity (${\beta \lambda }_{{PC}{2}_{{Env}}\,}$) outweighs over three-fold the estimated effect of the fast-slow continuum $({\beta \lambda }_{{PC}{1}_{{LH}}\,})$ (posteriors ${|\beta \lambda }_{{PC}{2}_{{Env}}\,}$/${\beta \lambda }_{{PC}{1}_{{LH}}}|$ = 0.036 /−0.017). However, the influence of environmental variability on population responses depends on the vital rate under consideration. For example, aridity is the main determinant of the contribution of individual growth to population responses to environmental variability (${E}_{{growth}}^{\sigma }$), with more variability in aridity being associated with lower demographic buffering (${E}_{{growth}}^{\sigma },$ ${\beta \lambda }_{{PC}{2}_{{Env}}\,}\,$= 0.006 [0.001 to 0.011]; Fig. 4b). In contrast, temperature and aridity act together to determine the importance of survival (${E}_{{survival}}^{\sigma }$) on reducing buffering in plants (${E}_{{survival}}^{\sigma },\,{\beta \lambda }_{{PC}{1}_{{Env}}:{PC}{2}_{{Env}}\,}\,$= 0.027 [0.011 to 0.044]; Fig. 4a). These findings likely reflect the greater dependence of plants, as sessile organisms, on their capacity to survive and grow under heatwave-induced drought events^31^.

## Discussion

The fast-slow and reproductive strategies continua of life history traits have long dominated predictions of species adaptation to environmental conditions^32,33^. However, these axes have somewhat limited power in predicting population responses to environmental variability^34,35^. Over the past two decades, a novel framework to examine population responses to environmental variability has emerged based on the idea that natural selection must canalise the temporal variation in vital rates to buffer against the expected negative impacts of environmental variability^16,28^, a prediction referred to as the ‘demographic buffering hypothesis’^16,28^. Despite significant progress in the pertinent theory^17^ and praxis^36^, we still lack a solid understanding of the drivers of population responses to environmental variability, their relationship to the well-known fast-slow and reproductive strategy continua, and the role of evolutionary history and environmental regimes^17,26^. Here, we provide a critical assessment of this knowledge gap and report several key findings: (i) Populations’ responses to environmental variability can be represented along a single axis—from more buffered to less buffered; (ii) This axis reflects the combined influence of species’ life-history (*i.e*., fast-slow and reproductive strategies), evolutionary history, and the environmental variability faced by each population; (iii) However, these relationships are complex, and considering phylogenetic relationships offers a different perspective. Notably, only the fast-slow continuum — but not the reproductive strategies continuum — remains an important predictor of populations’ responses to environmental variability; (iv) Yet, the predictive power of the fast-slow continuum on the ability of a population to buffer against the environment is outweighed by variation in aridity; (v) And finally, whilst plants’ populations responses to environmental variability are strongly determined by evolutionary history, contrary to predictions^37^, the phylogenetic signal is stronger in reproduction (sexual and clonal) instead of the survival – the vital rate where temporal variance contributes most to population growth rate.

In our study, environmental variability outweighs life history traits in predicting the buffering capacities of natural populations. This occurs because survival—the vital rate with the greatest influence on buffering and the lowest phylogenetic conservatism—is primarily shaped by environmental variation, causing environmental variability to be a stronger predictor than species-specific traits. This result is in line with the well-known challenge of predicting survival of individuals in a variable environment^31,35^. Here, we highlight three major challenges to use evolutionary history and life history traits to predict plant survival in a variable environment. First, organisms may possess life history traits that were once advantageous in the ancestral environmental regimes but, given the fast climate changing^1^, are no longer beneficial. For instance, some species have evolved specific windows for recruitment that were adaptive in environments with predictable fluctuations, but are maladaptive in current/future unpredictable environments^38^. Second, individual responses to environmental variability are highly heterogeneous and tend to be determined by their capacities to obtain and conserve energy rather than their evolutionary life history^35^. Third, covariation between physiological and morphological traits can amplify or dampen the impact of environmental variability on individual performance^31,39^. Consequently, environmental variability may result in nonlinear responses that pose important challenges for ecological forecasts^21,40^. Together, these challenges likely weaken the predictive power of the fast-slow and reproductive strategies axes, which do not explicitly consider demographic variation.

Because life history axes do not capture the effects of environmental and individual variation, we argue that they reflect how species have evolved to cope with past environmental regimes rather than the current or future pressures that populations are likely to face. On the other hand, these complex responses exemplify the numerous evolutionary processes that can influence temporal variation in vital rates, upon which the demographic buffering hypothesis was developed^19,41^. The existence of a demographic buffering continuum that quantifies the temporal variance in vital rates, as we report here, is a promising tool to generate new insights regarding which and how populations and communities^42^ respond to environmental variability. For instance, it is worth noting that plant populations with higher long-term performance (${\lambda }_{s}$) are located at intermediary levels of temporal variance in their vital rates, suggesting that some variance is potentially beneficial, but too much may not be (see fig. S1 for details).

Despite being subject to stronger phylogenetic conservatism, the contribution of reproduction to demographic buffering is relatively low compared to other vital rates. This finding contrasts with the high influence but low phylogenetic conservatism of survival. Together, these findings call for more research exploring how survival-reproduction trade-offs will shape population performance in the Anthropocene. For instance, recent studies have shown how reductions in survival might be compensated by increases in reproduction, a phenomenon known as demographic compensation^43,44^. Although a growing body of literature has supported the widespread existence of demographic compensation among natural populations^43,45,46^, their capacity to compensate against the negative impact of environmental variability remains poorly understood^43^. Given our finding that reproduction has a generally low contribution but a highly predictable influence on population responses to environmental variability, one could predict that its role in demographic compensation has a limited impact but can be anticipated with confidence. Investigating such a question is particularly important when predicting population responses to climate change^47^, especially under uncertain increases in environmental variability^1^. Additionally, we suggest that because the effects of reproduction on population responses to environmental variability are highly predictable by evolutionary history, there is an opportunity to improve the parameterisation and accuracy of demographic models in population viability analyses^48^. This opportunity includes models examining early impacts of exploitation, such as seed harvesting^49^, and might be particularly important for modelling annual plants, as reproduction is particularly important to drive their population response to environmental variability. Moreover, when survival, the most important and less predictable vital rate, cannot be fitted in model parameterisation, reducing uncertainty in other vital rates, such as reproduction, is imperative^48^. Our findings show a promising avenue for research in this direction.

It is critical to recognise that our framework, using the sum of stochastic elasticities of the stochastic population growth rate ${\lambda }_{s}$ with respect to vital rate temporal variance, $\sum {E}_{v}^{\sigma }$, has two areas for improvement. First, demographic buffering and its counterpart, the demographic lability hypothesis—which proposes that natural selection favours temporal variation in the most influential vital rates^17,21,41^—both rest on evolutionary mechanisms which have never been directly tested. Instead, stabilising (if buffered) and disruptive (if labile) selection have only been inferred, often via nonlinearity indices^19^ or second-order derivatives^41^, which cannot account for observed temporal variation and its effect on the stochastic growth rate ${\lambda }_{s}$. By contrast, our framework quantifies that temporal variation and its impact on population performance, but does not disentangle the selective processes (i.e., stabilising vs. disruptive selection) driving it. Thus, while our framework is not the final stop of this important line of enquiry, our framework provides a unique set of opportunities to complement recent evolutionary metrics such as nonlinearity indices^19^ and second-order derivatives^41,50^. Further studies may integrate such metrics into the present framework to link the forces of natural selection and their effects on the current and future performance of natural populations.

A second area for improvement is the suboptimal representation of long-term demographic studies across taxonomies and geographies required for comparative demographic studies. The existing databases we use here, though state-of-the-art regarding the number of demographic studies, remain biased toward perennial species in the Plant Kingdom^51,52^. For instance, while the robustness of survival as the main driver of buffering holds for most of our studied populations, annual plants may represent an alternative strategy for how survival-reproduction trade-offs shape population performance in variable environments. However, the limited sample size for annual plants limited any inference, pointing out the need for further studies. Similarly, given the small amount of available data on animal populations, we failed to perform similar analyses satisfactorily for comparison. With this taxonomic coverage, filling the demographic gap across continental scales, particularly in South America and Africa, remains a pressing issue to gain a holistic understanding of how natural populations respond to environmental variability^51,52^. It is worth noting that stochasticity in temperature shows a trend to increase from tropical areas to higher latitudes, suggesting further studies comparing demographic buffering across latitude might be an interesting venue. However, the acquisition and communication of high-resolution demographic data takes, by definition, a long time, which makes filling this gap an enduring commitment by population ecologists in the coming decades.

Ecology and conservation biology will improve the ecological forecasting of population responses to environmental conditions by considering a more flexible life history axis that explicitly accounts for the capacity of natural populations to respond to environmental variability: the demographic buffering continuum. Indeed, this continuum explicitly quantifies how populations respond to environmental variability by evaluating their effects on long-term population viability in real environments. By developing and applying the demographic buffering continuum here, we succeeded in quantifying the effect of environmental variability on population responses and linking these responses to life history traits and their evolutionary history. Our approach and findings highlight a promising way to better parameterise population models and improve population forecasts in the Anthropocene.

## Methods

To test if the demographic buffering capacity of plant populations could be predicted by life-history, environmental variability and evolutionary history, we developed a Bayesian Generalised Linear Mixed Model (GLMM) with and without phylogenetic corrections (see Eq. 1). Doing so allows us to test the robustness of our results to the role of shared ancestry in our comparative analyses. Below, we provide a detailed description of the analytical framework. Briefly, the analysis began with the establishment of a demographic buffering continuum of variance (Fig. S1). We then fitted separate models to assess the importance of five vital rates (survival, growth, shrinkage, reproduction, and clonality) in determining the position of species’ populations along said continuum. These models accounted for a life history and environmental variability in multivariate spaces derived from Matrix Population Models (MPMs^53,54^;) and environmental data extracted from CHELSAcruts^55^. Data collection, model parameterisation, and the overall analytical approach are detailed below.

### Demographic data and species responses to environmental variability

To assess the roles of vital rates in shaping stochastic population growth rates (${\lambda }_{s}$), we used time series of MPMs from natural populations. An MPM is a discrete-state mathematical representation of the life cycle of a species, typically represented by a matrix **A**. Each matrix element ${a}_{{ij}}$ in a **A** represents the contribution of a current (st)age to the next (st)age via survival, sexual and clonal reproduction, and development of individuals^53,56^. Development, in turn, can be decomposed into two processes: growth (development/progression to a larger or more advanced stage) and shrinkage (retrogression to a smaller or earlier stage). In total, five vital rates are entangled in the matrix **A:** survival, growth, shrinkage, reproduction, and clonality. To disentangle each vital rate, these **A** matrices can be decomposed into submatrices representing the different processes of the life cycle: the **U** submatrix describes survival-dependent transitions (e.g., progression, retrogression), while the **F** and **C** submatrices describe sexual and clonal reproductions, respectively^55^. The MPMs and their decomposed submatrices used in this study were selected from the COMPADRE Plant Matrix Database v6.23.5.0 ^29,57^, which contains 792 plant species with 8,994 MPMs.

Population responses to environmental variability were analysed by estimating the overall effect of temporal variation in vital rates on the stochastic population growth rate ${\lambda }_{s}$. Population responses were assessed via the well-established method of the sum of stochastic elasticities within respect to variance, $\sum {E}_{v}^{\sigma }$ ^30,41,56^. Briefly, this approach estimates the extent to which small changes in the mean and variance of a given vital rate *v* (or matrix element, ${a}_{{ij}}$) affect *λ*_*s*_. In mathematical terms, stochastic elasticity is the partial derivative of the vital rates *v* (or matrix elements, denoted by ${e}_{{ij}}^{s}$) over the MPM time series weighted by the relative contribution to λ_s_, and is typically expressed as ${e}_{{ij}}^{s}=({a}_{{ij}}\times \partial {\lambda }_{s})/({\lambda }_{s}\times \partial {a}_{{ij}})$ ^53^. Thus, ${e}_{{ij}}^{s}$ can be calculated iteratively based on how observed differences in ${a}_{{ij}}$ affected λ_s_^53^. The overall contribution of each vital rate $\sum {E}_{{ij}}^{s}$ to *λ*_*s*_ is always 1, but said overall contribution can be partitioned into the effect of perturbing the mean (*μ*) values of vital rate $\sum {E}_{{ij}}^{\mu }$ or its variance (*σ*), $\sum {E}_{{ij}}^{\sigma }$, such that $\sum {E}_{{ij}}^{s}$ = $\sum {E}_{{ij}}^{\mu }$ + $\sum {E}_{{ij}}^{\sigma }$ ^30,56^. The variance component $\sum {E}_{{ij}}^{\sigma }$ ranges between 0 to -∞, where values close to zero ($\sum {E}_{v}^{\sigma }\approx 0$) means that vital rate temporal variation in vital rates has a nearly negligible impact on ${\lambda }_{s}$ ^30,41^.

For illustration, consider a vital rate $v$ tracked over time in a population. Because environmental conditions are not static, some degree of change in these vital rates may be expected between the intervals *t* → *t* + 1, *t* + 1 → *t* + 2, etc. The differences in the values $v$ and their effect on ${\lambda }_{s}$ each time step represent the population’s response to the environment during the said time interval (or due to lag effects). The relative impact of variation in each vital rate $v$ on stochastic population growth ${\lambda }_{s}$ can be assessed by combining two components: its deterministic elasticity (which reflects how much changes in $v$ affect ${\lambda }_{s}$) and the temporal variability observed in $v$. When the observed variability in a vital rate is weighted by the vital rate’s deterministic elasticity, the result is the stochastic elasticity with respect to the variance, ${E}_{v}^{\sigma }$, which represents the proportional contribution of variance in the vital rate to changes in ${\lambda }_{s}$. Summing all ${E}_{v}^{\sigma }$ values results in $\sum {E}_{v}^{\sigma }$, which can be interpreted as the overall demographic impact of temporal variation in vital rates on ${\lambda }_{{s}}$ ^30,56^. In other words, $\sum {E}_{v}^{\sigma }$ represents how responsible variation in its vital rate $v$ is for population performance. In the absence of variation in vital rates, or if the variation occurs only in those vital rates that contribute less to ${\lambda }_{s}$, we should find $\sum {E}_{v}^{\sigma }\approx 0$. On the other hand, even small variation in vital rates with greater contribution to ${\lambda }_{s}$ is likely to increase the value of $\sum {E}_{v}^{\sigma }$ considerably.

Importantly, while previous works have developed this approach for matrix elements^30,41,58,59^, here we extend the method to evaluate the effects of the underlying components of matrix elements – vital rates, $v$ – thus offering higher resolution and allowing evaluating the impact of survival independent of other survival-development processes such as growth and shrinkage^60^. Unlike matrix elements, elasticities in the underlying vital rates might be negative^60^, thus the sum of stochastic elasticity of ${\lambda }_{s}$ with respect to vital rate temporal variance for the underlying vital rates ranges from -∞ to +∞. However, the main pattern persists, with $\sum {E}_{v}^{\sigma }\approx 0$ representing more demographically buffered populations, and $|\sum {E}_{v}^{\sigma }|\gg 0$ representing populations where temporal variation of vital rates strongly affects ${\lambda }_{s}$, either negatively or positively. As such, our approach explicitly accounts for the way that a given environmental regime may impact vital rates and how these vital rates, in turn, shape the overall performance of the population^30,56^. In our study, vital rates were retrieved from MPMs organised into three submatrices: **U,** **F** and **C**^54,60^. Specifically, survival, growth, and shrinkage were obtained from submatrix **U**, whereas sexual reproduction and clonal reproduction were estimated from the submatrices **F** and **C**, respectively. To do so, we used the family function *vr_* (*e.g*., vr_growth, vr_shrinkage*)* in the Rage R package^54^. Having access to already decomposed submatrices in COMPADRE, we explored the proportional contribution of each vital rate (*e.g*., ${E}_{{Survival}}^{\sigma }$, ${E}_{{Reproduction}}^{\sigma }$) to $\sum {E}_{v}^{\sigma }$, represented by $\left[\frac{|{E}_{v}^{\sigma }|}{\sum {{|E}}_{v}^{\sigma }|}\right] \times 100$.

The variable $\sum {E}_{v}^{\sigma }$ estimates population responses under variable environment by recognising multiple evolutionary and physiological processes. For instance, physiological constraints as well as canalising selection^61^ might limit variation^62^, both evolutionary processes pushing populations to the more buffered end of the demographic buffering continuum^41^. Meanwhile, disruptive selection and high phenotypic plasticity might support adaptive variation, thus pushing populations to the less-buffered end^21,40^. Furthermore, high values of $\left|\sum {E}_{v}^{\sigma }\right|$ indicate heightened responsiveness to temporal variation in vital rates, regardless of whether this temporal variation in vital rates is adaptive^30^. Despite limited inference, this approach overcomes existing approaches by: (1) considering the environmental variability experienced by populations not explicitly accounted in the nonlinearity index, which is calculated from their mean MPMs^19^; and (2) not being affected by constrained variation existing near the extreme values of vital rates that have puzzled the correlative approaches between vital rate variance and sensitivities^16,17,26^.

### Demographic data selection and phylogenetic relationships

To ensure comparability across and within species, we selected MPMs from COMPADRE that fulfilled a series of carefully planned selection criteria to ensure comparability and robust results: (1) only natural populations (*e.g*., no botanic garden data) to explicitly link vital rates to their natural environments; (2) studies containing MPMs with at least three annual contiguous censuses to capture environmental variability in the vital rates; (3) irreducible, primitive, and ergodic MPMs to ensure that each MPM represents a complete life cycle; (4) MPMs with separated **U,** **F**, and **C** submatrices that allow us to disentangle the effects of vital rates on the overall population growth rate obtained from the matrix **A**; (5) populations with known GPS coordinates, so we could match the environmental data during the study period (see below) to vital rate variation; and (6) MPMs whose species are present in a well-resolved phylogeny to test the role of phylogenetic inertia on demographic buffering continuum (*H*_*3*_). Finally, we removed five populations identified as outliers (see *Life history traits, life history axes, and the life history PCA*). These selection criteria yielded a total of 889 MPMs across 121 populations of 78 plant species.

To account for the evolutionary history in our models, we used the phylogenetic trees available in the MOSAIC database^63^. The trees are continuously updated from the EOL project^64^ and comprise most of the species available in COMPADRE. In them, polytomies were resolved using the multi2di function from the ape R package^65^. Further details regarding the construction of the phylogenetic trees are found in Salguero-Gómez et al.^14^.

### Life history traits and axes of variation

The position of each species’ populations along the fast-slow and reproductive strategy continua was used as covariates to test *H*_*1*_. To assess the position of each of the 121 populations along these two axes, we performed principal component analyses of the six examined life history traits. Life history traits define the timing, intensity, frequency, and duration of key demographic processes along the life cycle of any organism^7,8^. In life-history PCAs, life history traits are reduced to a series of dominant axes that capture the dominant combinations of life history traits and their trade-offs, thus offering a quantitative perspective on the emerging life history strategies (e.g., ref. ^66^). In plants and animals, PCAs of life-history traits have highlighted the existence of two dominant axes reflecting a trade-off between development and survival (the fast-slow continuum)^67,68^, and different reproduction strategies, from semelparous to extremely iteroparous (the reproductive strategies continuum)^68^.

Here, we derived the following six key life history traits from our examined MPMs: (1) individual development (*γ*); (2) mean life expectancy (*η*_*e*_), (3) distribution of mortality risk along the life cycle (*P*); (4) probability of achieving reproduction before dying (*p*_*α*_); (5) mean age at first reproduction (${L}_{a}$); and (6) reproductive window (*L*). A detailed description of how each vital rate was calculated is provided in the supplementary information (Table S1). The combination of these six life history traits in populations of plants adequately defines their life history strategies via multivariate analyses such as principal component analyses (PCA^13,14^). Once the life history traits were derived, we ensured a robust implementation of the PCA by removing outlier populations based on the Mahalanobis distance using the mahalanobis_distance function from the *rstatix* R package^69^. Briefly, this approach calculates the distance of each species population from the centre of the multivariate distribution and removes those populations whose distance is greater than expected by chance, assuming a 99% confidence interval.

We performed a PCA using the PCA function from the factoMineR package in R^70^. We retained the first two principal components (PC) because: 1) only these two first axes present eigenvalues higher than 1 following the Kaiser criterion — a common threshold used in PCA analyses; 2) these two axes together captured a similar amount of variation to that achieved by recent studies used to define the fast-slow and reproductive strategy continua^13,68^. Together, these ${PC}{1}_{{LH}}$ (life history PC1) and ${PC}{2}_{{LH}}$ explained 66.4% of the variation in life history traits (${PC}{1}_{{LH}}$: 44.95 %; ${PC}{2}_{{LH}}$: 19.55 %; Fig. S2). To quantify the position of each population in the two-dimensional space defined by ${PC}{1}_{{LH}}$ and ${PC}{2}_{{LH}}$, we obtained the PCA scores with the function scores of the Vegan R package v2.5.7^71^.

### Environmental data, environmental variability, and the environmental PCA

To test *H*_*3*_, if more variable environments push less buffered responses, we linked demographic responses to environmental variability. We started by quantifying the environmental variability experienced by our 121 examined populations, and extracted environmental information from CHELSAcruts^55^. To do so, we downloaded 1 km² gridded monthly values of maximum and minimum temperature and total monthly precipitation that corresponded to latitude and longitude, as well as the years of each study selected from COMPADRE. The CHELSAcruts database includes climatic information from 1901 to 2016, thus including the temporal extent of all populations used in this study (earliest start in 1938 and latest end in 2011; See Supplementary Data 1).

Because we are interested in demographic responses to environmental variability rather than mean temperature or precipitation, we decomposed each climatic variable $Y$ into three groups of variables that represent trend $(T)$, seasonality ($S$), and stochasticity ($R$). We derived these variables using a seasonal decomposition through an additive moving averages model toward the decompose function in the base R package^72^. Briefly, this function computes the trend component of the variable of interest (*e.g*., monthly temperature) using an additive moving average approach. This trend is then subtracted from the time series. The resulting time series represents the periodic fluctuation—the seasonality component ($S$). Finally, trend and season are discounted from the raw time series, and the residuals represent the stochasticity of the environmental component—*R*^72^. For each component of the time series $Y$ (*i.e*., ${T}_{Y},{S}_{Y},{R}_{Y}$), this approach allows us to derive the mean (${\bar{T}}_{Y},{\bar{S}}_{Y},{\bar{R}}_{Y}$), relative amplitude ($\frac{\max -\min }{{mean}}$; $\Delta {T}_{Y},\Delta {S}_{Y},\Delta {R}_{Y}$), and coefficient of variation (relative stochasticity hereafter, ${T}_{Y}^{{CV}},{S}_{Y}^{{CV}},{R}_{Y}^{{CV}}$). It is worth recalling that the studied variable (demographic buffering, $\sum {E}_{v}^{\sigma }$) captures the extent to which temporal variation in vital rates affected the population growth rate. Therefore, we retained only those climatic variables strictly related to changes in mean-variance environment and with direct biological or climate meaning, as these are the potential sources of variation in the vital rates. Thus, retained only the mean trend of the environmental conditions (${\bar{T}}_{Y}$), the amplitude of the trend component $(\Delta {T}_{Y})$, and the relative magnitude of the stochastic component within the environmental variability (${R}_{Y}^{{CV}}$). While ${R}_{Y}^{{CV}}$ represents the monthly environmental uncertainty (*i.e*., the stochastic component of environmental variability), the other two variables (${T}_{Y}$ and $\Delta {T}_{Y}$) collectively capture both the mean magnitude of the trend component(${T}_{Y}$) and the magnitude of fluctuations in that trend ($\Delta {T}_{Y}$) over the study period.

Similar to the procedures applied to the life history traits described above, we also applied a PCA to environmental data (Environmental PCA, ${{PC}}_{{Env}}$ hereafter) to retain those variables that best describe variation in environmental conditions across our 121 populations. Before performing the PCA, we assessed the collinearity among the environmental variables and removed those variables with high pairwise correlations to reduce redundancy ( | ρ | > 0.8 as the threshold; Fig. S3). As such, only the following six variables were further examined through our environmental PCA: (1) Mean value of the maximum temperature trend (${\bar{T}}_{{TempMax}}$), (2) relative stochasticity of maximum temperature (${R}_{{TempMax}}^{{CV}}$), (3) relative amplitude of the trend of the maximum temperature ($\Delta {T}_{{TempMax}}$), (4) relative amplitude of the trend of the minimum temperature$\,(\Delta {T}_{{TempMin}})$, (5) mean value of the precipitation trend (${\bar{T}}_{{Prec}}$), and (6) relative amplitude of the trend of the precipitation $(\Delta {T}_{{Prec}})$.

Similar to the PCA of life-history traits, we performed the PCA for the environmental variables using the *PCA* function from the *factoMineR* package in R^74^. We retained the first three axes because they show eigenvalues greater than 1, and together they explained 68.21% of the variation. Principal component axis 1 (${PC}{1}_{{Env}}$) explains 30.08% of the variation, and primarily reflects a gradient contrasting high mean maximum temperatures with high stochasticity in maximum temperature. Accordingly, populations with lower ${PC}{1}_{{Env}}$ scores are characterised by warmer but more stable temperatures (lower values of ${R}_{{TempMax}}^{{CV}}$), whereas those with higher ${PC}{1}_{{Env}}$ scores experience cooler but more variable thermal environments (Fig. S4). A closer examination of ${PC}{1}_{{Env}}$ reveals that it decreases towards the tropics, mirroring the expected trend of higher ${\bar{T}}_{{TempMax}}$ and lower ${R}_{{TempMax}}^{{CV}}$ in tropical regions (Fig. S5). In contrast, ${PC}{2}_{{Env}}$, which explains 20.59% of the environmental variation, describes differences in the amplitude of the trend in precipitation, $\Delta {T}_{{Prec}}$. ${PC}{2}_{{Env}}$ is higher in more arid places such as deserts and the Mediterranean region. Therefore, we refer to these environmental axes as coupled gradient of mean and stochastic thermal conditions (${PC}{1}_{{Env}}$) and aridity amplitude (${PC}{2}_{{Env}}$). Finally, the third axis of the environmental PCA, ${PC}{3}_{{Env}}$, explained 17.55% of the environmental variation and was related to the amplitude of maximum temperature in the time series ($\Delta {T}_{{TempMax}}$), which is likely to reflect how the environment has changed during the study time.

### Statistical analysis and the phylogenetic component

Once environmental variability ${{PC}}_{{Env}}$ and the population’s position along the life history axes ${{PC}}_{{LH}}$ are quantified, we started our inferential analyses by creating a set of competing phylogenetically corrected GLMMs to explain the population’s position along the buffering continuum, measured as $|\sum {E}_{v}^{\sigma }|$. Thus, a full model considering $\left|\sum {E}_{v}^{\sigma }\right|=\alpha+{\beta }_{{PC}{1}_{{LH}}}\times {\beta }_{{PC}{2}_{{LH}}}\times {\beta }_{{PC}{1}_{{Env}}}\times {\beta }_{{PC}{2}_{{Env}}}\times {\beta }_{{PC}{3}_{{Env}}}$ was created, as well as all possible combinations between ${{PC}}_{{LH}}$ and between ${{PC}}_{{Env}}$ (see Table S2). Next, these competing models were compared based on Deviance Information Criteria (DIC). Like the widely used Akaike Information Criterion (AIC), the DIC quantifies model fit and penalises complexity; however, it has been shown to be more appropriate for Bayesian analyses^73^. Similar to AIC, the model with the lowest value of DIC was assumed to be the most plausible, and differences in DIC greater than 2 were used to inform models that are statistically different. Accordingly to DIC, the best model is $\left|\sum {E}_{v}^{\sigma }\right|=\alpha+{\beta }_{{PC}{1}_{{LH}}}\times {\beta }_{{PC}{2}_{{LH}}}+{\beta }_{{PC}{1}_{{Env}}}\times {\beta }_{{PC}{2}_{{Env}}}$, so including all variables except ${\beta }_{{PC}{3}_{{Env}}}$. The full set of models compared is provided in supplementary information (Table S2). We then applied the same structure of the best model to explore: 1) the importance of each vital rate (e.g., Survival, $\sum {E}_{{Survival}}^{\sigma }=\alpha+{\beta }_{{PC}{1}_{{LH}}}\times {\beta }_{{PC}{2}_{{LH}}}+{\beta }_{{PC}{1}_{{Env}}}\times {\beta }_{{PC}{2}_{{Env}}}$); and 2) create a non-phylogenetically corrected model to better understand the role of phylogeny on determining buffering capacity for each model created.

A detailed look at the biological meaning of the equation is important for better understanding methodological decisions and the resulting biological interpretation in inferential analyses. The equation yielded by the best-fitting model ($\left|\sum {E}_{v}^{\sigma }\right|=\alpha+{\beta }_{{PC}{1}_{{LH}}}\times {\beta }_{{PC}{2}_{{LH}}}+{\beta }_{{PC}{1}_{{Env}}}\times {\beta }_{{PC}{2}_{{Env}}}$) indicates that a population’s buffering capacity against environmental variability (as quantified by $\left|\sum {E}_{v}^{\sigma }\right|$) can be predicted from its position along the two principal life-history axes and the two main environmental axes experienced during the study period. Because PCA reduces the dimensionality of the dataset by transforming variables into new orthogonal axes, it is good practice to assign each component a clear context-specific interpretation that highlights its biological implications. Finally, two more technical details need to be highlighted. First, all covariates in our model were *z*-transformed ($\mu=0$, and *sd* = 1). With this normalisation, we can compare the proportional contribution of each covariate to driving the population’s position along the demographic buffering continuum using estimates of *β* (effect size). For instance, covariates showing a positive effect size indicate an increase in $\left|\sum {E}_{v}^{\sigma }\right|$, thus reducing buffering. Second, we have distinguished between $\beta \lambda$ *and* $\beta$ to refer to the slope estimated (*i.e*., the effect size) of models with and without phylogenetic corrections, respectively.

All Bayesian Phylogenetic Generalised linear model was performed via the MCMCglmm R package^74^. The GLMMs were settled with a flat prior distribution $N\left({\mathrm{0,0.2}}\right)$ with 100,000 iterations. Finally, 95% of the posterior density distributions for each variable were compared and accepted as significant when they did not overlap 0 (fake p-value in the MCMCglmm output). Finally, we estimated the phylogenetic signal using the Pagel’s $\lambda$ (not to be confused with [stochastic] population growth rate $\lambda$ [${\lambda }_{s}$]). Briefly, Pagel’s $\lambda$ ranges between 0 and 1, where values close to 0 represent complete randomness in the examined variable and values close to 1 suggest a strong influence of evolutionary history determining the state of the variable^75^. We used a simple Spearman’s correlation to test if phylogenetic signal is positively associated with the variation in vital rates that contribute most to population buffering capacity.

### Reporting summary

Further information on research design is available in the Nature Portfolio Reporting Summary linked to this article.

## Supplementary information

Supplementary information

Description of Additional Supplementary Files

Supplementary Data 1

Reporting Summary

Transparent Peer Review file

## Data Availability

The demographic data used in this study were extracted from the COMPADRE Plant Matrix Database^29^ (v.6.23.5.0, accessed on 06 May 2023), available at https://www.compadre-db.org and cited as [29]. All data and associated code are available in the Zenodo database under accession code 18922678.

## Code availability

The code generated in this study is also provided in the Zenodo database under accession code 18922678.
