# Supplementary material for: Plant population responses to environmental variability are primarily driven by survival-reproduction trade-offs and mediated by aridity
Source: Nat Commun. 2026 May 28;17:6914. doi: 10.1038/s41467-026-73720-x (PMC13389012; doi:10.1038/s41467-026-73720-x)
Supplement: Supplementary file 1 — Supplementary information [file 41467_2026_73720_MOESM1_ESM.pdf]

SUPPLEMENTARY INFORMATION

Plant population responses to environmental variability are primarily driven by survival-reproduction trade-offs and mediated by aridity

Gabriel Silva Santos [1,2,3,*], Xianyu Yang [4,5] ; Samuel J L Gascoigne [3,6]; Aldo Compagnoni [7,8]; André T.C. Dias [9], Shripad Tuljapurkar [10], Maja Kajin [3,11], Roberto Salguero-Gómez [3,*]

[1] National Institute of the Atlantic Forest (INMA), 29650-000, Santa Teresa, Espírito Santo, Brazil

[2] Department of Ecology, Graduate Program in Ecology and Evolution, Rio de Janeiro State University, 524 São Francisco Xavier street, 20550-900, Maracanã, Rio de Janeiro, Brazil

[3] Department of Biology, University of Oxford, 11a Mansfield Road, OX1 3SZ, Oxford, UK

[4] Institute of Integrative Biology, ETH Zürich, Zürich, Switzerland

[5] WSL Swiss Federal Institute for Forest, Snow and Landscape Research, Birmensdorf, Switzerland

[6] School of Biological Sciences, University of Aberdeen, Aberdeen, UK

[7] iDiv. Puschstrasse 4, 04103 Leipzig, Germany

[8] Institute of Biology Martin Luther University Halle Wittenberg Am Kirchtor 1 06108 Halle / Germany

[9] Department of Ecology, Institute of Biology, Universidade Federal do Rio de Janeiro, Avenida Carlos Chagas Filho 373, 21941-590 Rio de Janeiro, RJ, Brazil.

[10] Department of Biology, Stanford University, Stanford CA 94305, USA

[11] Department of Biology, Biotechnical Faculty, University of Ljubljana, Večna pot 111, 1000 Ljubljana, Slovenia

[*] Corresponding authors

Gabriel Silva Santos: ssantos.gabriel@gmail.com

Roberto Salguero-Gómez: rob.salguero@biology.ox.ac.uk

TABLE OF CONTENTS

Supplementary Tables 3

Table S1. Technical procedures for life history traits derivation and PCA 3

Table S2. Full competing models in the model selection step 6

Table S3. Output of the best selected model 7

Table S4. Elasticities and proportional contribution of each vital rate to the sum of stochastic elasticities within respect to the variance $|\sum E_v^\sigma|$ 9

Supplementary Figures 10

Figure S1. Distribution of populations across the buffering continuum 10

Figure S2. Life history principal component analysis (Life history PCA) 11

Figure S3. Selection of environmental variables – Correlogram 13

Figure S4. Environmental Principal Component Analysis (Environmental PCA) 14

Figure S5. Global geographic distribution of environmental PCA loadings 16

Figure S6. Analysis with raw environmental variables 17

Additional Supplementary Files 19

References 19

SUPPLEMENTARY TABLES

Table S1

Table S1. Formulation of the life-history traits used to assess life-history strategies in the 121 populations of 78 species of plants. Notations used here are: λ is the deterministic population growth rate, corresponding to the dominant eigenvalue of the matrix **A** [1]; l_x and m_x refer to the age-specific survivorship and fertility schedules, respectively, for each age x. p_x is the proportional contribution of reproduction weighted by the population trajectory $p_x = \frac{l_x m_x \lambda^{\{-x\}}}{\sum_{x=0}^{xmax} l_y m_y \lambda^{\{-y\}}}$. The *xmax* refers to the maximum age observed during the study period; **U** and **F** are the submatrices of survival- and fertility-dependent processes, respectively, whereby **A** = **F** + **U**; **U′** is the survival-independent matrix of transition probabilities[1,2]; **w** is the stable stage distribution of the matrix **A**, and *i* and *j* are the row *and* column entries of the matrix population model respectively. Finally, the R function refers to the command used to calculate the life history traits with *Rage* package v.1.6.0[2]

Life history trait	Biological meaning	Formula	R function
Mean age at first reproduction (L_a)	Average amount of time from birth to reproduction	$L_a = \frac{\sum_{x=0}^{xmax} x l_x m_x}{\sum_{x=0}^{\max i} l_x m_x}$	*mature_age*

Probability of achieving reproduction before dying (p_α)	Probability of an individual become reproductive along their lifetime	$p_a = 1 - \prod_{x=0}^{xmax} (1 - l_x m_{x)}$	*mature_prob*
Mean life expectancy (η_e)	Expected lifetime of an individual at its birth	$e = \sum_{x=0}^{xmax} lx$	*life_expect_mean*
Reproductive window (*L)*	Temporal spread of reproduction throughout life span as quantified by Demetrius[3] entropy (L). High/low L values correspond to iteroparous/semelparous populations	$L = -\sum p_x \ln p_x$	*shape_surv*
Individual development (γ)	Mean probability of transitioning forward to a larger/more developed stage in the life cycle of the species, weighted by the stable stage distribution, **w**	$\gamma = \sum_{1}^{m} \overline{U'}_{i,j} \overline{w}_j \big\|_{i<j}$	*vr_growth*

Distribution of mortality risk along the life cycle (*P*)	Shape of the age-specific survivorship curve l_x as quantified by Keyfitz' entropy[4]. *P* >1, =1, <1 correspond to species whose mortality hazards decrease, stay constant, or increase with age respectively.	$P = \frac{-\log(l_x) l_x}{\sum l_x}$	*shape_rep*

Table S2

Table S2. Competing explanatory models for demographic buffering in plants $|\sum E_v^\sigma|$. Models are informed according to degrees of freedom (df) and Deviance Information Criterion (DIC). Models are ranked from lowest to highest DIC. The model with lowest DIC was used in posterior MCMCglmm analyses. Variables included in model selection were those axes with eigenvalues greater than 1: $PC1_{LH}$ (fast–slow continuum) and $PC2_{LH}$ (reproductive-strategy continuum; see Fig. S2) from the life history PCA. From the environmental PCA, we used the $PC1_{Env}$ (variation in temperature), $PC2_{Env}$ (variation in aridity), and $PC3_{Env}$ (amplitude of temperature). Two null models were considered: a standard null model, where none of the variables are informative – named *Null model*, and a second null model where climatic information is no informative – named *Climatic null model*)

Models	df	DIC
$|\sum E_v^\sigma| \sim PC1_{LH} * PC2_{LH} + PC1_{Env} * PC2_{Env}$	9	-400.7529
$|\sum E_v^\sigma| \sim PC1_{LH} * PC2_{LH} + PC1_{Env} * PC2_{Env} * PC3_{Env}$	13	-396.5775
$|\sum E_v^\sigma| \sim 1$ (*Null model*)	3	-384.2591
$|\sum E_v^\sigma| \sim PC1_{LH} * PC2_{LH} + PC3_{Env}$	7	-379.2992
$|\sum E_v^\sigma| \sim PC1_{LH} * PC2_{LH} + PC2_{Env}$	7	-376.4730
$|\sum E_v^\sigma| \sim PC1_{LH} * PC2_{LH} + PC2_{Env} * PC3_{Env}$	9	-376.4730
$|\sum E_v^\sigma| \sim PC1_{LH} * PC2_{LH} + PC1_{Env} + PC2_{Env}$	7	-376.1849
$|\sum E_v^\sigma| \sim PC1_{LH} * PC2_{LH} + PC1_{Env} + PC2_{Env}$	8	-373.1743
$|\sum E_v^\sigma| \sim PC1_{LH} * PC2_{LH} + PC1_{Env} + PC2_{Env} + PC3_{Env}$	9	-372.6623
$|\sum E_v^\sigma| \sim PC1_{LH} * PC2_{LH} + PC1_{Env} * PC3_{Env}$	9	-364.3317
$|\sum E_v^\sigma| \sim PC1_{LH} * PC2_{LH}$ (*Climatic null model*)	6	-342.2609
$|\sum E_v^\sigma| \sim PC1_{LH} * PC2_{LH} + PC1_{Env}$	7	-340.1330
$|\sum E_v^\sigma| \sim PC1_{LH} * PC2_{LH} * PC1_{Env} * PC2_{Env} * PC3_{Env}$	34	-331.3077
$|\sum E_v^\sigma| \sim PC1_{LH} * PC2_{LH} * PC1_{Env} * PC2_{Env}$	18	-257.7108

Table S3

Table S3. The outputs of the models with and without phylogenetic corrections based on the most plausible model, after model selection. Effect size here means the extent to which the axes of life-history and environmental principal component analyses (PCAs) determine the position of our 121 plant populations along the buffering continuum, as quantified by $|\sum E_v^\sigma|$. Note that higher values of $|\sum E_v^\sigma|$ indicate a higher responsiveness of population growth rate (λ_s) to variation in vital rates. Thus, negative effect sizes indicate variables that help reduce the temporal variation in vital rates and consequently stabilise λ_s. Model outputs include posterior means estimated for each covariate (effect size) and its 95% credible interval (95% CI). We also show the effective sample size used to estimate the parameters (eff.samp) along the iterations in the Monte Carlo Markovian Chain and a p-value equivalence (pMCMC). Covariates are considered to significantly drive the buffering capacities of plant populations $|\sum E_v^\sigma|$ when the 95% CI do not cross zero (bold lines).

		Effect size on $|\sum E_v^\sigma|$ (posterior mean)	95%CI	eff.samp	pMCMC
Phylogenetically corrected model					
	Intercept	0.068	[-0.276 - 0.410]	9900.000	0.676
	**Fast-slow ($PC1_{LH}$)**	**-0.011**	**[-0.022 - 0.000]**	**722.010**	**0.042**
	Rep. strategy ($PC2_{LH}$)	-0.006	[-0.024 - 0.013]	263.650	0.520
	Temperature ($PC1_{Env}$)	0.017	[-0.017 - 0.052]	1393.830	0.323
	**Aridity ($PC2_{Env}$)**	**0.036**	**[0.008 - 0.060]**	**1552.420**	**0.017**
	Fast-slow × Rep. strategy ($PC1_{LH} \times PC2_{LH}$)	0.007	[-0.004 - 0.017]	72.020	0.168
	Temperature × Aridity ($PC1_{Env} \times PC2_{Env}$)	0.014	[-0.008 - 0.035]	204.420	0.177
Non-phylogenetically corrected model					
	**Intercept**	**0.066**	**[0.030 - 0.102]**	**10176.00**	**0.001**
	Fast-slow ($PC1_{LH}$)	-0.018	[-0.004 - 0.004]	9900.00	0.105
	Rep. strategy ($PC2_{LH}$)	-0.026	[-0.065 - 0.015]	9900.00	0.204
	**Temperature ($PC1_{Env}$)**	**0.042**	**[0.012 - 0.072]**	**9571.00**	**0.007**

	Aridity ($PC2_{Env}$)	0.020	[-0.015 - 0.056]	9900.00	0.250
	**Fast-slow × Rep. strategy ($PC1_{LH} \times PC2_{LH}$)**	**0.023**	**[0.002 - 0.044]**	**9603.00**	**0.035**
	**Temperature × Aridity ($PC1_{Env} \times PC2_{Env}$)**	**0.041**	**[0.017 - 0.064]**	**9900.00**	**0.001**

Table S4

Table S4. Percentage of contribution of variation in each vital rate v, E_v^σ, to the overall effect of temporal variation in on the stochastic population growth rate λ_s, which is represented by $\sum E_v^\sigma$. Life forms follow COMPADRE v6.23.5.0.[6] The number of populations analysed is denoted by N.

Life forms	N	$E^{\sigma}_{Survival}$	E^{σ}_{Growth}	$E^{\sigma}_{Reproduction}$	$E^{\sigma}_{Shrinkage}$	$E^{\sigma}_{Clonality}$
Annual	4	9.32	0.04	84.5	6.14	0
Epiphyte	1	56.6	1.77	39.7	1.94	0
Herbaceous perennial	92	48.9	20.7	18.3	5.73	6.30
Palm	3	41.8	24.4	21.8	10.9	1.05
Shrub	3	71.5	27.7	0.37	0.48	0
Succulent	10	60.9	26.7	4.95	7.50	0
Tree	8	67.2	28.0	1.84	2.91	0

Supplementary Figures

Figure S1

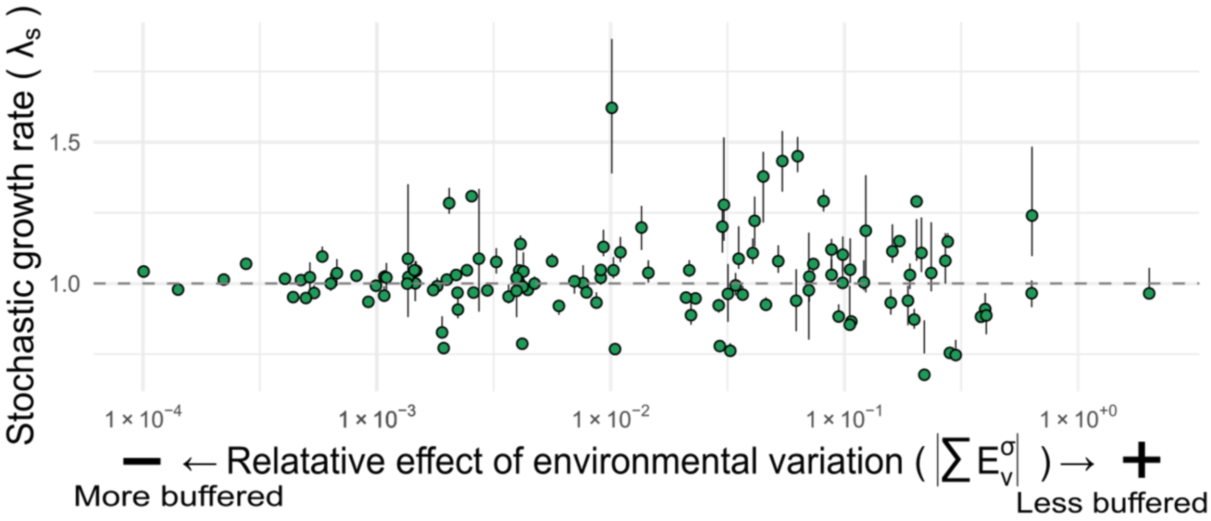

Figure S1. Plant populations span along a continuum of demographic buffering against the effect of temporal variation in vital rates. Said continuum is quantified by the sum of stochastic elasticities within respect to the variance $|\sum E_v^\sigma|$ in the vital rates (*i.e.*, survival, individual-level growth, individual-level shrinkage, reproduction, and clonality). Points and bars represent the mean and the 95% confidence interval, respectively. Values of $|\sum E_v^\sigma|$ represent the extent to which changes in the temporal variation of a given vital rate affect the stochastic population growth rate (λ_s) in 121 natural populations of 78 species of plants retrieved from the COMPADRE (v. 6.23.5.0) database. At the buffering-end of the continuum (left; $|\sum E_v^\sigma|$ ~0), vital rates either vary less or the observed variation has little to negligible effect on λ_s. At the not buffered end (right; $|\sum E_v^\sigma|$ >>0), the observed variation in vital rates strongly shapes λ_s values.

Figure S2

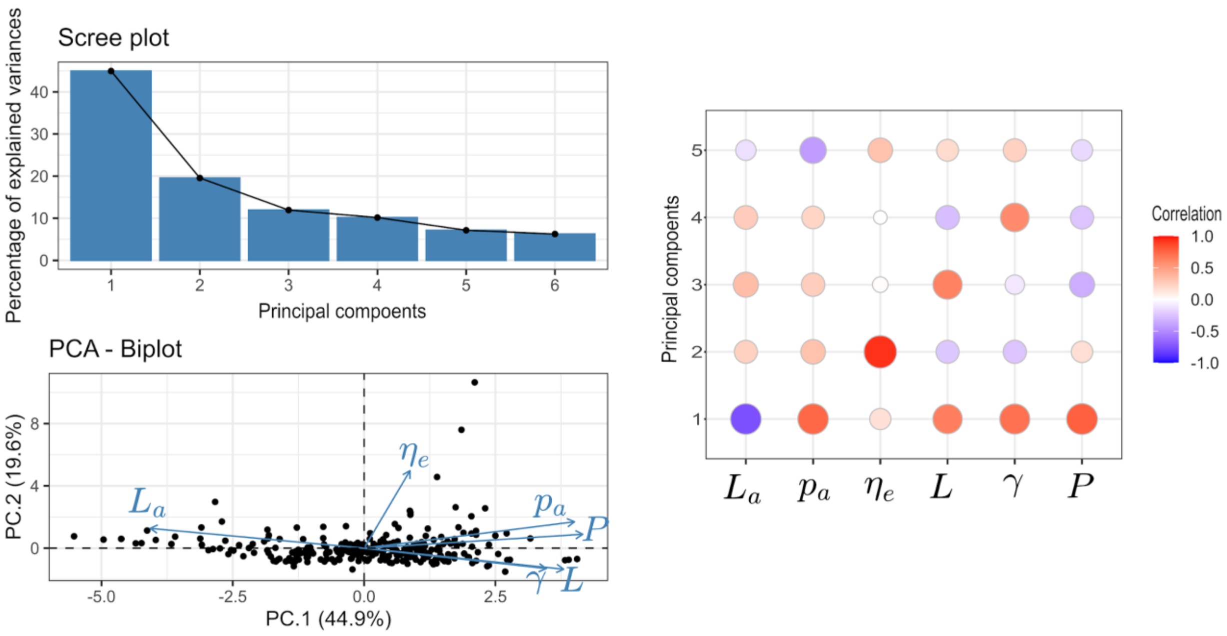

Figure S2. Results from the life history principal component analysis (PCA_{LH}) are depicted, showing that the first two principal components are better represented by mean age at first reproduction (L_a), probability of achieving reproduction before dying (p_a), and Mean life expectancy (η_e). The panel figure shows the scree plot, biplot and correlation plot related to the principal components and the environmental variables. In the top left panel, the scree plot supports that the first two principal components (PCs) capture most of the variance (44.9% and 19.5%, respectively); moreover, these were the only axes with eigenvalues greater than 1, suggesting that they represent the most important patterns in the pace-of-life. $PC1_{LH}$ is more strongly associated with all aspects of the life history traits, representing how individuals allocate their energy between survival and reproduction – the fast-slow continuum. On the other hand, $PC2_{LH}$ is strongly associated with life expectancy, where longer life-spans are related to continuous investment in reproduction (iteroparity) rather than a single reproductive event

(semelparity). These relationships are better visualised in the right panel, which shows how each PC is related to the key life history traits derived. In the bottom left panel, the biplot depicts how the 121 studied populations of plants are distributed along the environmental gradients captured by the first two PCs. The arrows represent the life history traits, with their lengths indicating their relative contribution to each PC. The notations represent six key life history traits: individual development (γ); mean life expectancy (η_e), distribution of mortality risk along the life cycle (P); probability of achieving reproduction before dying (p_α); mean age at first reproduction (L_a); and reproductive window (L).

Figure S3

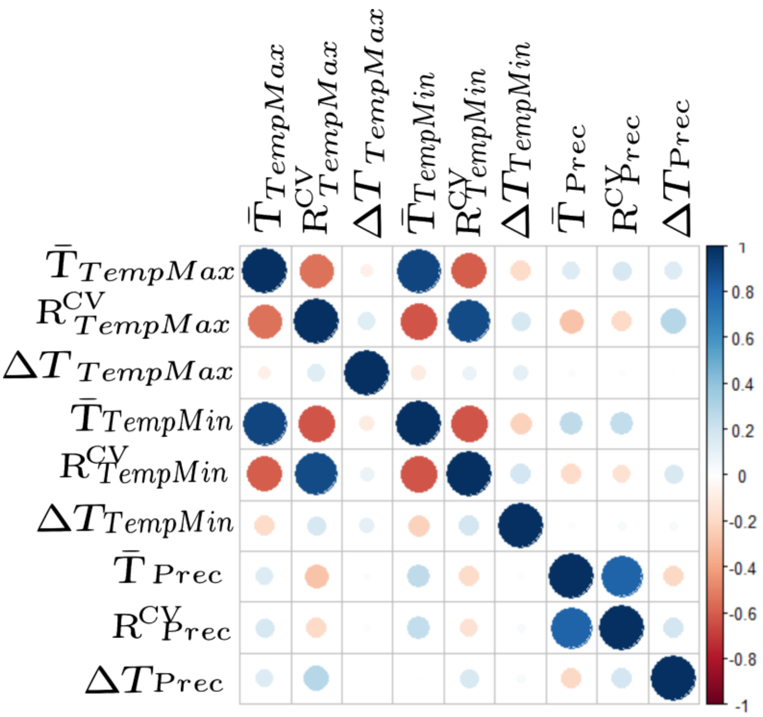

Figure S3. Correlogram showing the pairwise correlations used to identify multicollinearity among environmental variables. Notations represent the following variables: mean trend ($\bar{T}$), amplitude of the trend (ΔT) and proportional environmental stochasticity (R^{CV}). The colours and size represent Pearson's correlation (ρ). When variables are highly correlated (|ρ| > 0.8), indicating potential multicollinearity, only one of the correlated variables was selected for the environmental PCA (see figure S4).

Figure S4

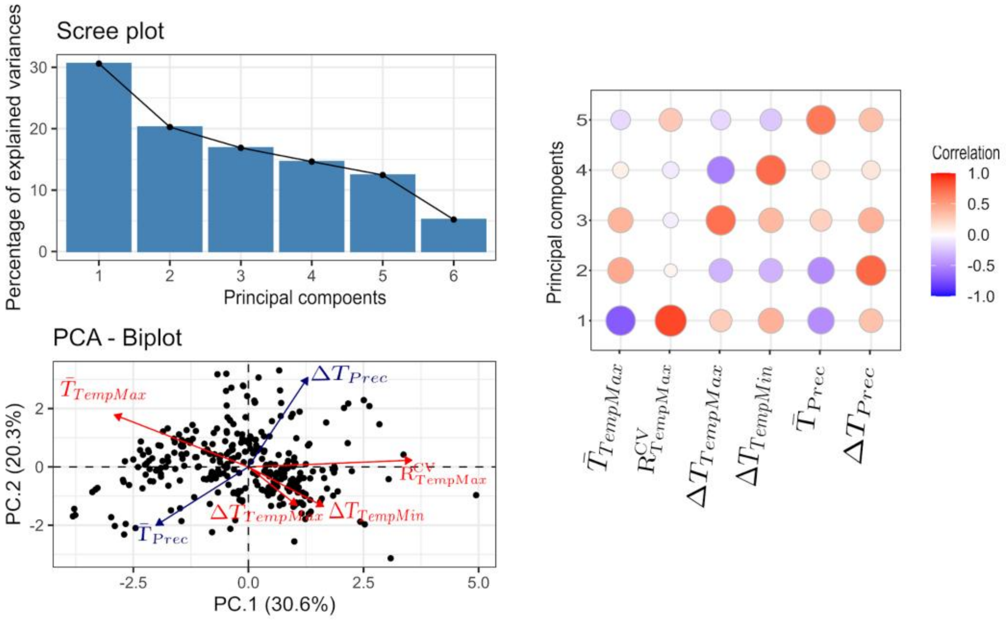

Figure S4. Results from the Environmental Principal Component Analysis (PCA_{Env}) are presented, showing that the environmental variability is better represented by three axes related to temperature and aridity. The panel figure shows the scree plot, biplot and correlation plot related to the principal components and the environmental variables. In the top left panel, the scree plot supports that the first three principal components (PCs) capture most of the variance (30.1%, 20.6% and 17.5%, respectively), suggesting that they represent the most important patterns in the environmental data, but there is still a large amount of variation to be explored by further research. In the bottom left panel, the biplot depicts how the 121 studied populations of 78 species of plants are distributed along the environmental gradients captured by the first two PCs. The arrows represent the environmental variables; their lengths indicate their relative contribution to each PC, and their colours are set to blue for variables related to precipitation and red for variables related to temperature. Notations represent the following variables: Mean value of the maximum temperature trend ($\bar{T}_{TempMax}$), relative stochasticity of maximum temperature

($R^{CV}_{TempMax}$), relative amplitude of the trend of the maximum temperature ($\Delta T_{TempMax}$), relative amplitude of the trend of the minimum temperature ($\Delta T_{TempMin}$), mean value of the precipitation trend ($\bar{T}_{Prec}$), and relative amplitude of the trend of the precipitation (ΔT_{Prec}). $PC1_{Env}$ captures a coupled gradient of mean and stochastic thermal conditions, reflecting the well-known latitudinal covariance between mean climate and variability. On the other hand, $PC2_{Env}$ is primarily driven by the variation in precipitation, as can be more clearly seen in the correlation plot on the right side of the panel. Finally, $PC3_{Env}$ is more closely related to the amplitude of maximum temperature during the study period, but this variable was not included into the most plausible model to explain the population's position along the buffering continuum (see Table S2).

Figure S5

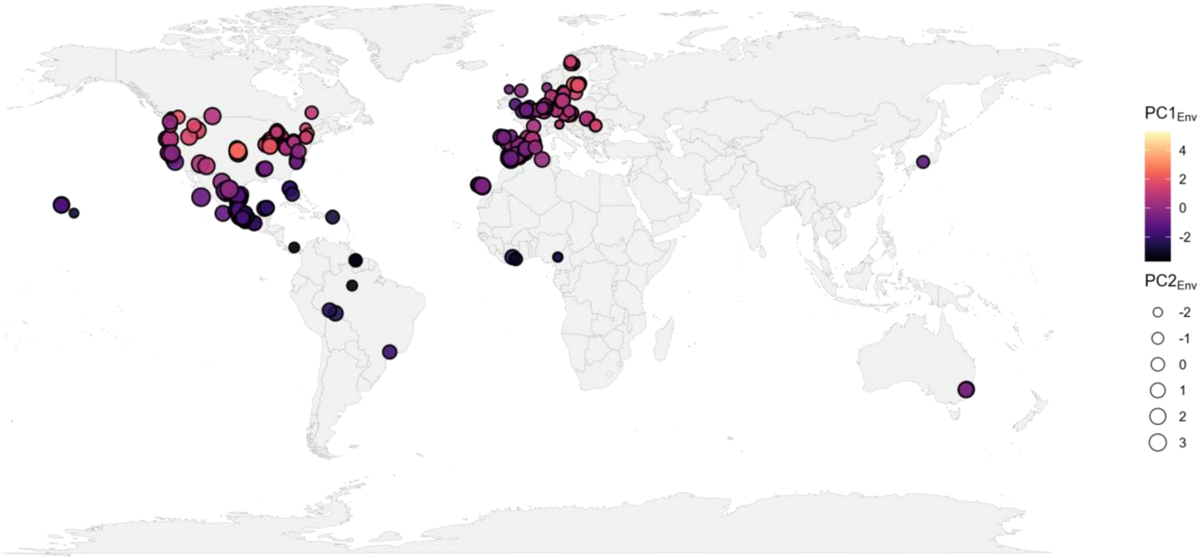

Figure S5. The 121 studied populations from 78 plant species are mapped together with their positions along the environmental gradients captured by the environmental principal component analysis. $PC1_{Env}$, which represents a coupled gradient of mean and stochastic thermal conditions, shows a noticeable trend of increasing with latitude. Despite a more elusive trend, it is possible to identify that $PC2_{Env}$ increases from continental to insular areas; however, this pattern might be masked by topographic features (e.g. altitude), which are not shown. World map silhouette was extracted from rnaturalearthdata package v.1.0.0.[5].

Figure S6

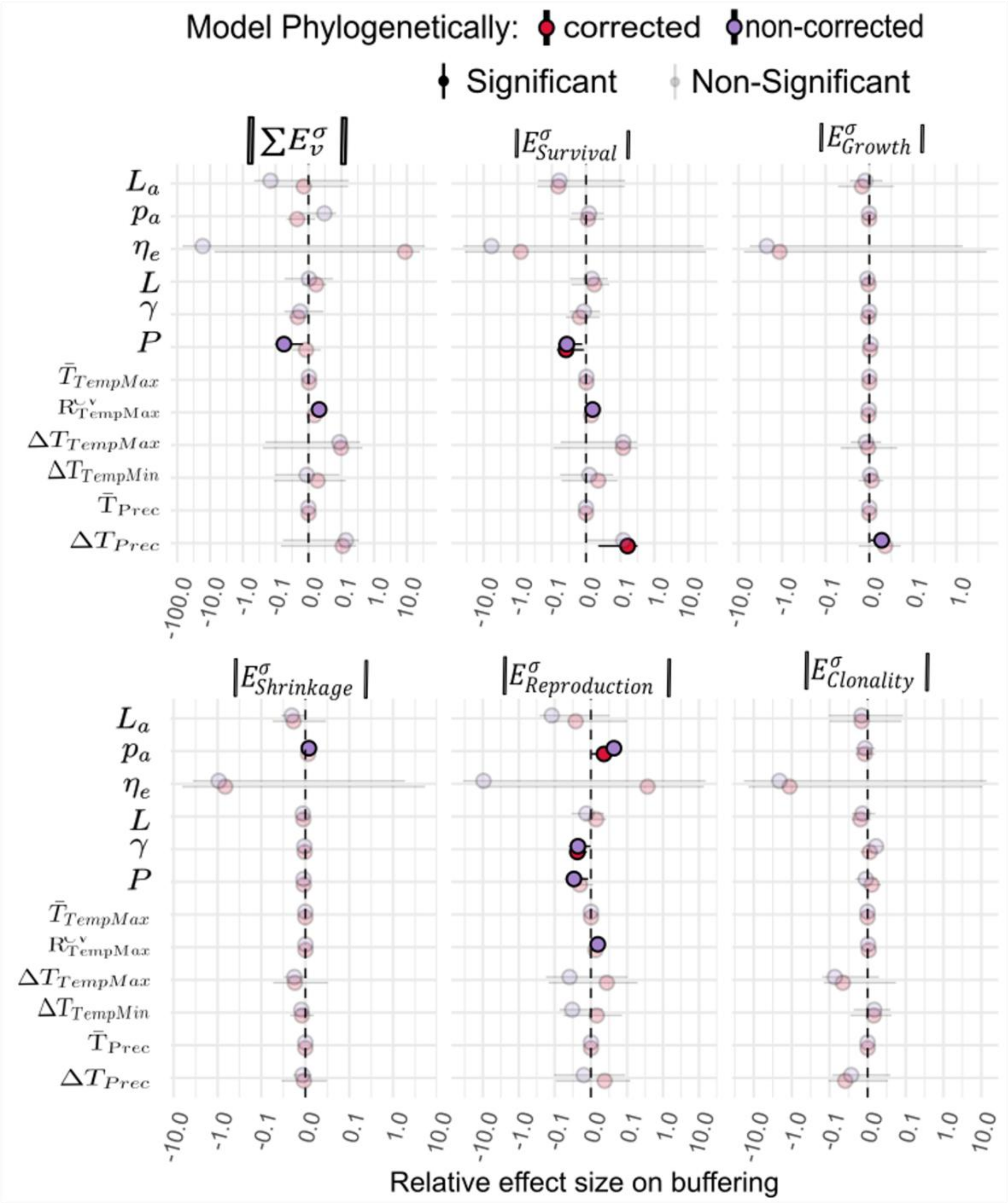

Figure S6. Effects of life-history traits and environmental variables on the positions of the 121 plant populations studied along the buffering continuum and on the contributing vital rates. MCMCglmm models with (red) and without (purple) phylogenetic correction

are shown. Points and bars represent the posterior medians and 95% credible intervals, respectively. Models are additive and do not include interactions. Notations represent six life-history traits and six environmental variables as follows. Life-history traits: individual development (γ); mean life expectancy (η_e), distribution of mortality risk throughout the life cycle (P); probability of achieving reproduction before dying (p_α); mean age at first reproduction (L_a); and reproductive window (L). Environmental variables: mean trend in maximum temperature ($\bar{T}_{TempMax}$); relative stochasticity of maximum temperature ($R^{CV}_{TempMax}$); relative amplitude of the trend of the maximum temperature ($\Delta T_{TempMax}$); relative amplitude of the trend of the minimum temperature ($\Delta T_{TempMin}$); mean value of the precipitation trend ($\bar{T}_{Prec}$); and relative amplitude of the trend of the precipitation (ΔT_{Prec}).

Additional Supplementary Files

- **Source Code and Analytical Framework.** Full scripts and step-by-step framework available at https://doi.org/10.5281/zenodo.18922678.

References

1. Caswell, H. *Matrix Population Models: Construction, Analysis, and Interpretation*. (Sinauer Associates, Sunderland, Mass, 2001).
2. Jones, O. R. *et al.* Rcompadre and Rage—Two R packages to facilitate the use of the COMPADRE and COMADRE databases and calculation of life-history traits from matrix population models. *Methods Ecol Evol* **13**, 770–781 (2022).
3. Demetrius, L. Demographic Parameters and Natural Selection. *Proc. Natl. Acad. Sci. U.S.A.* **71**, 4645–4647 (1974).
4. De Vries, C., Bernard, C. & Salguero-Gómez, R. Discretising Keyfitz' entropy for studies of actuarial senescence and comparative demography. *Methods Ecol Evol* **14**, 1312–1319 (2023).
5. South, A., Michael, S. & Massicotte, P. rnaturalearthdata: World Vector Map Data from Natural Earth Used in 'rnaturalearth'. 1.0.0 https://doi.org/10.32614/CRAN.package.rnaturalearthdata (2017).
6. COMPADRE Plant Matrix Database 2023. Available from: https://www.compadre-db.org [06 May 2023, Version 6.23.5.0].
