# Supplementary material for: Plant population responses to environmental variability are primarily driven by survival-reproduction trade-offs and mediated by aridity
Source: Nat Commun. 2026 May 28;17:6914. doi: 10.1038/s41467-026-73720-x (PMC13389012; doi:10.1038/s41467-026-73720-x)
Supplement: Supplementary file 2 — Description of Additional Supplementary Files [file 41467_2026_73720_MOESM2_ESM.pdf]

Description of Additional Supplementary File

Supplementary Data 1 - Supplementary Data containing the metadata for all studied populations, including species names, geographic coordinates, and primary literature citations
