# Supplementary material for: Plant population responses to environmental variability are primarily driven by survival-reproduction trade-offs and mediated by aridity
Source: Nat Commun. 2026 May 28;17:6914. doi: 10.1038/s41467-026-73720-x (PMC13389012; doi:10.1038/s41467-026-73720-x)
Supplement: Supplementary file 5 — Transparent Peer Review file [file 41467_2026_73720_MOESM5_ESM.pdf]

nature portfolio

Peer Review File

Plant population responses to environmental variability are primarily driven by survival-reproduction trade-offs and mediated by aridity

Corresponding Author: Dr Gabriel Santos

This file contains all reviewer reports in order by version, followed by all author rebuttals in order by version.
Parts of this Peer Review File have been redacted as indicated to remove third-party material.

Version 0:

Reviewer comments:

Reviewer #1

(Remarks to the Author)
This paper addresses an important ecological topic—how populations respond to environmental stochasticity, with demographic buffering emerging because of complex evolutionary and demographic processes. The authors propose a valuable measure of demographic buffering and explore how it relates to environmental regimes, life history traits, and phylogenetic history. The study finds that while both plants and animals experience demographic buffering, plants display stronger evolutionary constraints, with phylogeny playing a larger role. Aridity plays a major role in reducing buffering in plants, likely due to plants' dependence on temperature and precipitation for survival and growth. In contrast, animals exhibit more variability, with life history traits and environmental influences playing less predictable roles in their responses to environmental stochasticity. However, despite the relevance and novelty of the topic, I found the paper challenging to read and follow. As a result, I struggled to identify clear, conclusive patterns and felt confused about the main points the study aimed to convey. Given the complexity of the concepts surrounding demographic buffering, I wonder if it would be more beneficial to present this work in a longer article format rather than a short article. It seems that the depth of the topic, along with the need for additional clarity and methodological detail, would be difficult to achieve within the constraints of a word limit.

Throughout the manuscript, the phrasing used to describe population responses implies an intentional or active modulation of vital rates. For example:
• L24: "Populations can modulate how their vital rates... change through time"
• L27: "...this ability of natural populations to buffer against environmental stochasticity"
• L47: "...how populations minimise the expected negative effects of environmental stochasticity."
Please rephrase these sentences to emphasize that demographic buffering results from evolutionary and demographic processes rather than intentional modulation.

The Introduction introduces several concepts (e.g., fast-slow continuum, reproductive strategy continuum, Jensen's inequality, demographic buffering) in rapid succession. While each is relevant, the way they are presented makes it difficult to see how they fit together.

Similarly, the Results section is packed with statistical details, which makes it hard to extract the most important results.

The conclusion that animals do not align with the fast-slow continuum or reproductive strategies could be influenced by data limitations rather than reflecting a true biological pattern, especially given that only three years of empirical data were used as a filter for species inclusion. Additionally, the database contains a disproportionate number of plant species compared to animal species (78 plant species versus 11 animal species), which may skew the conclusions.

The figures are quite complex due to the multifactorial analyses. Including a schematic figure that summarizes the main results could help improve clarity for readers.

Additionally, the methods section lacks sufficient detail—specifically, the equation for the sum of stochastic elasticities with respect to variance is not fully described. I also found it unclear how the life history traits were derived from the data (line

429), and further clarification on this process would be beneficial.

(Remarks on code availability)

Reviewer #2

(Remarks to the Author)
Manuscript 24-4911-T aims to examine the drivers of species' abilities to buffer against environmental stochasticity. This is an exciting idea with important implications especially under ongoing climate change. However, I do not think the analyses provide a robust test of the proposed idea. I have several large concerns about the interpretation of results that I detail below.

Major concerns:

1. Many of the results compare animals and plants, (e.g., Lines 139-146,156-158), but the analyses include 121 populations belonging to 78 species of plants versus 13 populations depending to 11 species of animals. This makes for a very unequal sample size, and further making inferences for "animals" as a broad category based on 11 species is a huge over interpretation. I understand that this data is hard and time consuming to collect, and I appreciate the thorough cleaning of the data that removed many of the populations, but regardless have concerns about any inferences about animals broadly or comparisons between animals and plants given the sample size of animals.

2. The inferences about the role of environmental stochasticity do not robustly isolate stochastic effects—the main framing of the manuscript—for two reasons.
2a. The first reason is that inferences are made based on the variance of each vital rate observed from the matrix population models, sum(E^\sigma_v). Low variances in observed population vital rates could arise due to small effects of environmental stochasticity, as the authors claim. However, low variances in population vital rates could also arise due to small experienced environmental variation during the time series—even if the population is highly responsive to environmental stochasticity. If I am understanding correctly, this metric does not account for these two alternatives as currently presented, and thus cannot be used to examine sensitivity to environmental stochasticity generally. As a side note, time series length across the different data sets could also alter sum(E^\sigma_v), as longer term time series are more likely statistically to have experienced an extreme event drawing from the tail of the environmental distribution.
2b. The manuscript uses PC1Env and PC2Env to examine effects of the environment, claiming that PC1Env describes environmental stochasticity while PC2Env describes seasonal aridity. While I do not have any concerns about this approach generally, I have concerns about the interpretation. 6 environmental variables went into the PCA analyses, including many effects unrelated to environmental stochasticity, such as mean value of the maximum temperature, relative amplitude of seasonality in minimum temperature etc. Therefore, even though PC1Env largely correlates with CV of maximum temperature, it does not directly quantify environmental stochasticity, making claims about the effects of environmental stochasticity based on PC1Env fraught. Further, the claim (line 480) "The first two PCs emerging from this next step explained 50.9% of the variation in environmental stochasticity" is false, given that other components of the environment that are not part of stochasticity (mean value of maximum temperature) were included in the PCA analysis. Similarly, but a smaller concern, is referring to PC2Env as seasonal aridity throughout the manuscript, since aridity could itself be directly measured. Rather, I suggest referring to this as PC2Env and just stating that PC2 largely correlates with the aridity gradient.

3. I think it is important to examine life history strategy interactions with environment variables (Figures 3, 4 and corresponding analyses), as the fast-slow strategy might greatly influence species' buffering capacities in highly variable environments, but less so in less variable environments. I can easily envision interactive effects with aridity/PC2Env as well.

Minor Comments:
1. Lines 151-153. Would an alternative interpretation be that the reproductive strategy continuum is important for populations' responses, but that reproductive strategy is largely phylogenetically conserved?

2. Lines 121-125, Figure 2 and Table S1. The results discuss the average effect across animals versus plants. Is this from a weighted average based on coverage in the database? This general statement doesn't hold for many of the taxa, especially for the statement that individual-level growth comes second in terms of importance (e.g. reproduction is key for arthropoda, and annual plants).

3. Results: referring to figure subpanels would be helpful.

4. Figure 2: For annual plants, is seed banking ability included in the models? If not, the extreme result for reproduction is unsurprising. It is not clear to me how seedbanking would fit into the vital rates, but is a critical aspect to include for analyzing annual plants.

5. Figures 3 and 4: Suggest changing "Estimated effect size" to "Effect on sum(E^\sigma_v)" or something similar so the reader knows what the response variable of interest is.

6. Ln 266: Define the demographic liability hypothesis.

(Remarks on code availability)

Reviewer #3

(Remarks to the Author)
This manuscript represents a sizable contribution to eco-evolutionary theory by putting commonly held notions on life-history continuums into an appropriate environmentally-influenced and phylogenetically corrected-for context. I found the paper and it's findings genuinely interesting and I think this will be a well-citated piece of work. My main comments are related to the structure of some sections, in that the wording can be a bit opaque or indirect in places and so it can take several read-through's to fully grasp what the authors are saying. I hope my comments are helpful in this respect.

Specific comments
Line 70-80: it's not super clear to me why one would 'expect' H1b, given the authors have set up why these two continua should be related to demographic buffering in the previous paragraph. I get that there are caveats (eg Jensen's inequality) but the text doesn't fully set up why we would expect this to be the case as a hypothesis. This is made clearer below, but I just feel like there is something off with the structuring of this paragraph of the intro such that the progression of ideas isn't super clear, and since it's a key point of the paper I think it just needs a slight bit of reorganization.

Line 125 – This may show my animal-bias, but I don't fully understand shrinkage and how traits related to shrinkage are not the same as those related to growth/survival. The methods section also lists it but doesn't really describe it. Again, given that it is a key finding, I think laying out explicitly what shrinkage is and what it means would be really helpful for the reader.

Line 127 – There is something I'm missing here. In the intro the authors state that their hypothesis '(H1b) the position of species' populations along the demographic buffering continuum is not predicted by the fast-slow and reproductive strategies continua', but here say 'Our analyses provide support for H1b in plants, but not in animals. Indeed, the position of plant populations along the demographic buffering continuum, as quantified by $|\sum E_v^{\sigma}|$, is predicted by the fast-slow and reproductive strategies continua'. I get that the authors then state that this isn't the case when phylogenetic ancestry is considered (and then mostly focus on plants), but the wording here is quite confusing. It took me a few readings of the full paragraph to get what the authors mean here, and so I expect others might get tripped up as well.

Line 173, does the '>> 0' symbol not indicate 'much lower than zero', whereas here the authors mean 'approaching zero'

Line 201 – saying that plants and animals fall along a single axis in their responses to environmental stochasticity seems a bit off since only one axis is investigated. Just a slightly word clarification here would help

Line 479 – There is something here that doesn't seem right. Is 'Delta T precip' supposed to be the amplitude in trend of precipitation (not temperature)? As written it doesn't make sense with the result below of PC2Env

Line 788 should read 'Pearson's correlation'

Line 794 – PC3 here explains very nearly as much variance in PC2. Is there a formal criteria the authors used to decide on two PCs instead of more? It's not super clear from just the scree plot.

Line 834- I particularly like the inclusion of the table listing all the studies/species included in the analysis. Good for transparency.

(Remarks on code availability)

Version 1:

Reviewer comments:

Reviewer #1

(Remarks to the Author)
Thank you for your thorough and careful revisions. The focus on plants has significantly improved the clarity of your results, and the updated figures are much more effective in conveying your key points. However, I still find the manuscript quite technical in both its framing and language. While your contributions to understanding demographic buffering are valuable, some sections—particularly the discussion of Jensen's inequality and the shape of environment–vital rate reaction norms—remain difficult to follow for a broader audience. This may limit the accessibility of your findings beyond a specialist readership.
I also remain unconvinced by the emphasis on convex (U-shaped) reaction norms as a key conceptual innovation. While this is an interesting mathematical possibility, from a biological standpoint U-shaped functional relationships are not commonly expected. More often, bell-shaped (∩-shaped) curves—such as those seen in thermal performance or tolerance—are anticipated due to physiological limits or trade-offs. The relevance of U-shaped norms for vital rates is not clearly justified, and I would encourage you to either provide stronger biological motivation or shift the emphasis toward more ecologically grounded scenarios. Despite these concerns, I found your work to be rigorous and intellectually engaging.

(Remarks on code availability)
NaN

Reviewer #2

(Remarks to the Author)
This is Reviewer 2 from the first round of revisions. First off, I really appreciate all of the effort that the authors clearly put into the revision. I think that the focus on the plant data makes the manuscript much stronger. The clarification to the writing and figures has also made the manuscript less dense and much easier to read. Adding analyses, such as examining time series length, incorporating PC3 etc., have made conclusions more robust than in the previous version. I can tell this was a lot of work, and think the manuscript is much improved.

I still have one major concern remaining for the revision, which is that the link between demographic results and environmental stochasticity as a driver is not, in my view, robust. This relates to previous comment 2b, response R10b>, and the interpretation of PC1 as environmental stochasticity.

To clarify, I have no concerns about the validity of the statistical tests, but rather their interpretation, which I do not feel was addressed in the last round of revision—I think this was just a miscommunication between myself and the authors on the comment. To hopefully clarify for this round, the authors arguments for H3 relies on the role of PC1, which they argue is equivalent to environmental stochasticity. Yet, in PCA analyses, all six environmental variables do (to varying levels) contribute to different PC axes. Indeed, from Figure S4 (right hand side), it looks like bar(T)_TempMax also contributes very significantly to PC1, with other environmental variables, such as bar(T)_Prec contributing, but with a more moderate correlation coefficient. Thus, from the PCA analyses, it is not robust to say that relationships with PC1 are driven by environmental stochasticity. Rather, these results could (nearly) equally be driven by negative relationships with bar(T)_TempMax, given the strong correlation with PC1.

As such, an equivalent interpretation and/or framing for the manuscript, given the statistical tests, could center on the role of the mean value of the maximum temperature in driving demographic buffering. In other words, the authors could use these exact analyses (or nearly so) to write a paper about the role of the mean trend of maximum temperature in driving demographic buffering, including a brief caveat sentence that this environmental variable had a strong positive relationship with mean minimum temperature and a strong negative correlation with relative stochasticity of maximum temperature (from Figure S3). At the end of the day, the challenge lies in the strong correlations between environmental drivers, which is a challenge across a lot of ecology! These strong correlations mean that multiple variables map onto the same PCA axis, it appears. Further, given these strong correlations arise in the datasets analyzed here, interpreting this as the effect of environmental stochasticity alone is a concern to me.

I note that in the authors' response, they state that they couldn't remove the mean trend of temp, and I agree. The problem isn't that it is included, but rather that it ALSO contributes a decent amount to PC1, and as such interpretation of results of PC1 cannot be as simple as environmental stochasticity, since other environmental variables – that do not relate to stochasticity whatsoever – also contribute to PC1.

Instead, I wonder about actually examining the environmental stochasticity effects directly themselves, rather than through a PCA analysis, with the different PC axes used as covariates. Doing so would allow for incorporating other environmental covariates that the authors think/know are important, while directly interpreting stochastic effects themselves. Even with this, including a discussion about the challenges in disentangling environmental stochasticity effects is critically, especially given the strong correlations in environmental covariates.

All of this led to an important, but more minor comment, which is that the manuscript never defines environmental stochasticity. I think this is really important to do in the first or second paragraph, especially given confusion in the literature about stochasticity vs. variability vs. heterogeneity vs. unaccounted for "randomness."

Finally, I note that neither H1 or H2 rely on interpreting environmental stochasticity, which means that many of the results feel really strong to me, despite my longwinded comment above. It's H3 that is problematic, and, to me, reads as the key framing for the manuscript. Yet, H1 and H2 are still really interesting, especially with the revised wording for H2 (which helped me a lot).

(Remarks on code availability)

Reviewer #3

(Remarks to the Author)
I am satisfied that the authors have adequately dealt with my previous comments/concerns, and agree that this represents a substantially clearer representation of their findings. My only comment is that I would advise the authors to perhaps re-order the legend in Figure 2 to match the order in which each group appears (top to bottom) in the figure itself. As such, it took me a bit of time to realize that the different life forms were represented by different colors, even though this was indicated in the legend.

(Remarks on code availability)

Version 2:

Reviewer comments:

Reviewer #1

(Remarks to the Author)
I am satisfied with the revisions. The manuscript is clear, and I have no further comments.

(Remarks on code availability)

Reviewer #2

(Remarks to the Author)
After a careful read through of the revised version of “Plant population responses to environmental stochasticity are primarily driven by survival-reproduction trade-offs and mediated by aridity” my major concerns from the last revision remain unaddressed.

The authors still argue that PC1_Env represents environmental stochasticity, but the table they show in the response letter and their supplementary figures S3, S4, and S5 show that PC1 represents multiple components of the environment. It represents a latitudinal gradient, which in turn represents changes in mean temperature (here bar(T_TempMax)) and variability in temperature (here stochasticity or R CV TempMax) in temperature. In the response they argue that this axis represents primarily environmental stochasticity but it has a correlation of 0.87 with stochasticity while T_TempMax has an almost equally strong negative correlation of -0.74. This is unsurprising and a classic challenge for inferring underlying environmental drivers across a latitudinal gradient, given it is well known that these climate covariates strongly covary, as occurs in this study as well.

The issue remains that the main text introduction, results, and discussion still fully attribute this effect to environmental stochasticity, with only a single sentence at the end (lines 308-310) mentioning this critical fact in the main text. While the methods text is now more precise, this nuance is not translated into the manuscript framing or results. The issue remains that the authors attribute effects to environmental stochasticity when the correlation structure of the covariates makes it difficult or impossible to disentangle temperature stochasticity effects from mean temperature effects from latitudinal effects.

In the response letter, the authors note, “Given the limited importance of PC1_Env, any potential concerns regarding the biological interpretation of this axis should be considered of minor importance (R2e).” If this is the case, it calls into question for me the main framing focused on the role of environmental stochasticity, including: (a the title “plant population responses to environmental stochasticity are primarily driven by survival-reproduction trade-offs and mediated by aridity,” (b) the key take away “(i) Populations’ responses to environmental stochasticity can be linked to life-history strategies and environmental stochasticity along a single axis of variation, from more buffered to less buffered” (lines 210-212), and (c) further discussion points such as “environmental stochasticity outweighs life history traits in predicting the buffering capacities of natural populations” (lines 224-225). Given the current manuscript framing, environmental stochasticity is represented by PCA1_Env. So, if this axis is of limited importance, this contradicts key take away points throughout the manuscript.

The revision adds in a definition of environmental stochasticity equating temporal variability with environmental stochasticity (lines 43-44). This is incorrect. Environmental stochasticity refers to random fluctuations in the environment, while temporal variability includes deterministic trends such as seasonality too – as the authors account for in their analyses. Having a precise definition is critical for the manuscript.

This lack of precision also occurs in the Results/Methods when discussing PCA2_Env. In the results, this axis is referred to as “temporal variation in aridity” (line 184), “stochasticity in aridity” (line 189), and just “aridity” (line 190). In the methods, it is stated that this axis is referred to as “seasonal aridity” (line 554) which is a shorter description of “relative amplitude of the trend of the precipitation” (line 554). While this could be a relatively minor fix in the main text, the comment in the response letter that PC1_Env is of "minor importance” made me wonder if the authors were also including PC2_Env as a measure of stochasticity, as stated on line 189. However, this axis has a very small loading from the stochasticity variable (R CV TempMax) according to Figure S4, so does not represent stochasticity.

(Remarks on code availability)

Version 3:

Reviewer comments:

Reviewer #2

(Remarks to the Author)
I appreciate the authors' revisions during the last round and response to comments. My previous concerns have been fully addressed with the changes in wording throughout the manuscript, and I appreciate the authors' efforts.

(Remarks on code availability)
NA

Open Access This Peer Review File is licensed under a Creative Commons Attribution 4.0 International License, which permits use, sharing, adaptation, distribution and reproduction in any medium or format, as long as you give appropriate credit to the original author(s) and the source, provide a link to the Creative Commons license, and indicate if changes were made.
In cases where reviewers are anonymous, credit should be given to 'Anonymous Referee' and the source.
The images or other third party material in this Peer Review File are included in the article's Creative Commons license, unless indicated otherwise in a credit line to the material. If material is not included in the article's Creative Commons license and your intended use is not permitted by statutory regulation or exceeds the permitted use, you will need to obtain permission directly from the copyright holder.
To view a copy of this license, visit https://creativecommons.org/licenses/by/4.0/

9NCOMMS-24-49711-T

We are grateful for the reviewers' thorough and constructive feedback and appreciate their insightful recommendations We have implemented a careful revision based on these recommendations which we agree significantly improved the robustness of our claims and enhanced the clarity of our findings. The main modifications include:

- Following concerns regarding the unbalanced sample sizes between plants (78 species) and animals (11 species) in our original submission, and given our inability to digitise sufficient demographic information of animal species in a feasible timeframe given multiple competing demands, we have decided to remove the animals from our revised manuscript. As such, the manuscript now only deals with the responses of natural plant populations to environmental stochasticity. In turn, this modification makes the narrative more focused and enables a more detailed discussion of our findings, with clearer links to potential mechanisms. See responses R5 and R20 for how we addressed the removal of animals, and R4 for how this removal improved the manuscript's readability.
- To improve the readability of the manuscript, we also have contextualised the phylogenetic signal into a new hypothesis, where we predict that vital rates with greater contribution to the stochastic population growth rate λ_s should display a higher degree of phylogenetic conservatism in regards to their contribution to $|\sum E_v^\sigma|$. This hypothesis, as detailed in the last paragraph of the Introduction (see new lines 90-93; also, on R3 in this document), is based on the idea that buffering capacity in vital rates is determined by natural selection and that, as such, it should show strong phylogenetic patterns across the Tree of Life (of Plantae, in our case). The support for this hypothesis is presented in Figure 3 (see R4), and its importance to improve readability is discussed in R12.
- Because our modelling approach used in our GLMM was subject of some concerns by Reviewers #2 and #3, we rerun the analyses including a model selection procedure to improve the robustness of our analyses without significant changes in the main conclusions of our manuscript. See R11 and R26 below.
- Because reviewers discussed the complexity of the figures and that the results are packed with too many details, we have also reanalysed the values of each vital rate now as absolute values. This change has helped standardise the biological meaning and interpretation of $\sum E_v^\sigma$ and E_v^σ. We note, however, that this analytical change has not changed our main conclusions. See R4 and R6 below.
- Finally, following constructive feedback by the editor and reviewers, we have improved the readability of the entire manuscript and simplified its figures by (i) separating phylogenetic signal in a different figure; and (ii) adding extra elements to guide readers to understand how estimated effect size on the analysed variables represent in terms of more or less buffering. We argue that these changes have rendered this version much easier to read for a general audience. See R3 and R4 below.

Below, we provide a point-by-point account of whether and how the feedback was integrated (and, when not, why). The responses are chronologically numbered (*e.g.,* R1>, R2>, etc.) to facilitate cross-referencing where appropriate.

Reviewer #1

This paper addresses an important ecological topic—how populations respond to environmental stochasticity, with demographic buffering emerging because of complex evolutionary and demographic processes. The authors propose a valuable measure of demographic buffering and explore how it relates to environmental regimes, life history traits, and phylogenetic history. The study finds that while both plants and animals experience demographic buffering, plants display stronger evolutionary constraints, with phylogeny playing a larger role. Aridity plays a major role in reducing buffering in plants, likely due to plants' dependence on temperature and precipitation for survival and growth. In contrast, animals exhibit more variability, with life history traits and environmental influences playing less predictable roles in their responses to environmental stochasticity. However, despite the relevance and novelty of the topic, I found the paper challenging to read and follow. As a result, I struggled to identify clear, conclusive patterns and felt confused about the main points the study aimed to convey. Given the complexity of the concepts surrounding demographic buffering, I wonder if it would be more beneficial to present this work in a longer article format rather than a short article. It seems that the depth of the topic, along with the need for additional clarity and methodological detail, would be difficult to achieve within the constraints of a word limit.

R1> We appreciate your positive appraisal of our work. We have thoroughly revised the work, addressing all comments provided by editor and reviewers without significantly increasing the length of the manuscript. This improvement has been, in part, achieved by focusing only on plants, following R5 and R9 below. Furthermore, we have engaged with two additional native English-speaking colleagues, not experts in population ecology, whose comments have helped us make sure that the revised version is clearer and easier to follow by the broad readership of *Nature Communications.*

Throughout the manuscript, the phrasing used to describe population responses implies an intentional or active modulation of vital rates. For example:

- L24: "Populations can modulate how their vital rates... change through time"
- L27: "...this ability of natural populations to buffer against environmental stochasticity"
- L47: "...how populations minimise the expected negative effects of environmental stochasticity."

Please rephrase these sentences to emphasize that demographic buffering results from evolutionary and demographic processes rather than intentional modulation.

R2> We thank the reviewer for this comment. We have rephrased the sentences to ensure that the buffering capacity is presented as an eco-evolutionary consequence of

the morpho-behavioural-physiological response to environmental stochasticity. Revised examples (with updated line numbers) include:

Lines 24-26 (summary): "Natural populations may suffer negatively from increased environmental stochasticity due to climate change; however, several mechanisms can mitigate those effects by changing the vital rates of a population (e.g., survival, reproduction)."

Lines 26-28(summary): Despite important analytical and theoretical advances, we still do not know how and to what extent environmental regimes, life history traits, and evolutionary history determine the buffering capacity of natural populations.

Lines 45-47: "… understanding whether and how the expected negative effects of environmental stochasticity can be minimised has become a primary mission of Ecology, Evolution, and Conservation Biology[6]."

The Introduction introduces several concepts (e.g., fast-slow continuum, reproductive strategy continuum, Jensen's inequality, demographic buffering) in rapid succession. While each is relevant, the way they are presented makes it difficult to see how they fit together.

R3> Thanks for pointing it out. We have carefully rewritten the main sections of the introduction to make the flow and balanced introduction of key terms work better for a non-expert audience. Specifically, we have substantially rewritten the third and fourth paragraphs of the introduction to provide a clearer explanation of why environmental stochasticity matters and how it can outweigh the contribution of life-history traits in determining a population's position along the buffering continuum, as shown below. Passages in bold were carefully crafted to improve the readability in the Jensen's inequality as well as the importance of fast-slow continuum and reproductive strategies in our study.

Lines 61-78: *So far, the link between life history continua and demographic buffering remains largely untapped for multiple reasons. First, we still lack a general understanding of how each vital rate drives species responses to environmental stochasticity. Second, the demographic buffering hypothesis[17,19] often assumes linear or concave (∩-shaped) shapes of environment-vital rate reaction norms[20–22], which indeed tends to result in negative effects on long-term of stochastic population growth (λ_s) when environmental variation increases. Alternatively, convex (∪-shaped) reaction norms may represent an entirely different outcome, yet this possibility remains largely overlooked in demographic buffering analyses[21].* ***In convex (∪-shaped) vital rate reaction norms, environmental variability may amplify the beneficial outcomes of higher values of the vital rate, thus outweighing the detrimental effects of lower values[21,24] via the so-called Jensen's inequality[23]. In other words, environmental variability is beneficial when the mean stochastic population growth rate λ_s, is higher than deterministic population growth rate, λ.*** *Importantly, there are limits to the amount of variance that*

a vital rate can exhibit without driving a population locally extinct[25]. Third, ***environmental stochasticity has, to date, not been explicitly accounted for in studies examining demographic buffering****.* ***Consequently, we argue that natural populations reported to show reduced temporal variability in vital rates during the study timeframe might have been incorrectly interpreted as buffered.*** *Together, these complementary and necessary dimensions to test the demographic buffering hypothesis remain a major challenge to link life history continua to population responses to environmental stochasticity.*

Lines 81-104: Here, we examine how environmental regimes, key life history traits, and evolutionary history predict the extent to which natural populations of plants can buffer against environmental stochasticity along a demographic buffering continuum. Specifically, we parameterise a phylogenetically-corrected Bayesian Generalised Linear Mixed Model (GLMM) with high-resolution vital rate data from 121 natural populations of 78 plant species to test the following hypothesis: (H_1) the position of plant species' populations along the demographic buffering continuum is only weakly predicted by the fast-slow and reproductive strategies continua. Although a study using 36 populations of different species found that longevity (a life history trait aligned with the fast-slow continuum[10,11,14]) is a good proxy of demographic buffering[18], a more recent study with a greater number of populations and accounting for their evolutionary history provided opposing evidence[25]. Moreover, because each vital rate responds differently to environmental variability[26], it is challenging to anticipate a general pattern of temporal variation across all vital rates, thus blurring our current understanding of buffering in organisms. To better understand the contribution of vital rates on demographic buffering, we test (H_2) whether the phylogenetic signal of the buffering capacities of the examined species is stronger in the vital rates that contribute most to λ_S, since demographic buffering is a result of natural selection[16,27]; ***Finally, none of the existing studies exploring demographic buffering have explicitly considered the amount of environmental stochasticity faced by the studied population. This omission likely inflates the perceived roles of life history traits and phylogenetic relationships in explaining population responses to environmental variability, which leads us to hypothesise (H3) that the extent to which a natural population displays demographic buffering strongly depends on its environmental regime****. Specifically, higher stochasticity in precipitation and temperature is expected to lead to greater variances in vital rates, shifting populations toward the less buffered end of the demographic buffering continuum.*

Similarly, the Results section is packed with statistical details, which makes it hard to extract the most important results.

R4> In retrospect, we agree with the reviewer that the results section was rather packed. We have implemented several key changes to aid in the clearer identification of our main findings:

- We have rewritten the result section to improve its clarity as well as to accommodate the removal of animals in our analyses (see R5). The focus is now exclusively on plants, which keeps the narrative more centred. This focus allows us to provide a more detailed presentation of the role of each vital rate in determining plant populations' positioning along the buffering continuum, which helps better understand the main results presented in Figures 2 and 3. See the new paragraph below.

> **Lines 142-158 (2nd paragraph of the results section showing that each vital rate driven the populations position along the buffering continuum differently):** "*It is important to consider that each vital rate contributes differently to demographic buffering and presents a different degree of phylogenetic conservatism. Indeed, the position of populations along the demographic buffering continuum is mainly determined by the temporal variance of survival ($E^{\sigma}_{Survival}$ = 50.26 ± 27.60%; Figure 2b), followed by individual-level growth (E^{σ}_{Growth} = 21.13± 21.01%) and reproduction ($E^{\sigma}_{Reproduction}$ = 18.12± 27.05%), with minor contributions from individual-level shrinkage ($E^{\sigma}_{Shrinkage}$ = 5.67 ± 10.12 %) and clonality ($E^{\sigma}_{Clonality}$ = 4.81± 18.57 %; Figure 2b and Table S5). However, contrary to our expectations (H_2), we did not find a positive relationship between the vital rates that contribute most to this buffering capacity and their phylogenetic signal (r=-0.7, S=34, p-value=0.230). Indeed, survival, the vital rate that contributes most to buffering, shows the weakest phylogenetic signal (Pagel's λ of $E^{\sigma}_{Survival}$ = 0.240; Figure 3). On the other hand, the contributions of temporal variation in sexual reproduction and clonality to λ_s ($E^{\sigma}_{Reproduction}$ and $E^{\sigma}_{Clonality}$) are highly phylogenetically conserved, as revealed by the strongest phylogenetic signal (Pagel's λ of $E^{\sigma}_{Reproduction}$= 0.972 and Pagel's λ of $E^{\sigma}_{Clonality}$ = 0.823, respectively; Figure 3). This high phylogenetic signal is also evident in the GLMM models, particularly for reproduction, where the contribution of the fast-slow continuum ($PC1_{LH}$) on determining $E^{\sigma}_{Reproduction}$ disappears when phylogenetic relationships are accounted for in our analyses (see Figure 4d).*"

- To address the high complexity of the figures (see R6), we have created new figures without animals (Figures 1-4) where we have separated the phylogenetic signal into a different figure (Figure 3), and have rearranged the order to improve the reading flow. Altogether, these changes improved the overall readability of this section.

- We have also changed Figure 2 to represent the percentage of contribution of vital rates to the cumulative effect of vital rate variation in stochastic population growth rate $|\sum E^{\sigma}_{v}|$, instead of presenting the raw values. With these changes and

the restructuring of the second paragraph, readers should find it easier to understand how vital rates contribute differently to the positioning of different plant populations along our buffering continuum.

- We have added new elements to our new figures to help improve their clarity. For instance. Figures 1 and 4 now show marks informing about more buffered and less buffered populations. For figure 4, the analyses are now based on absolute values of E_v^{σ}, which make them comparable with $|\sum E_v^{\sigma}|$(results presented in figure 1).

- We have reviewed the legend of each figure, which has resulted in some significant changes in figures 1, 2, and 4.

The modified figures and their new figure legends are:

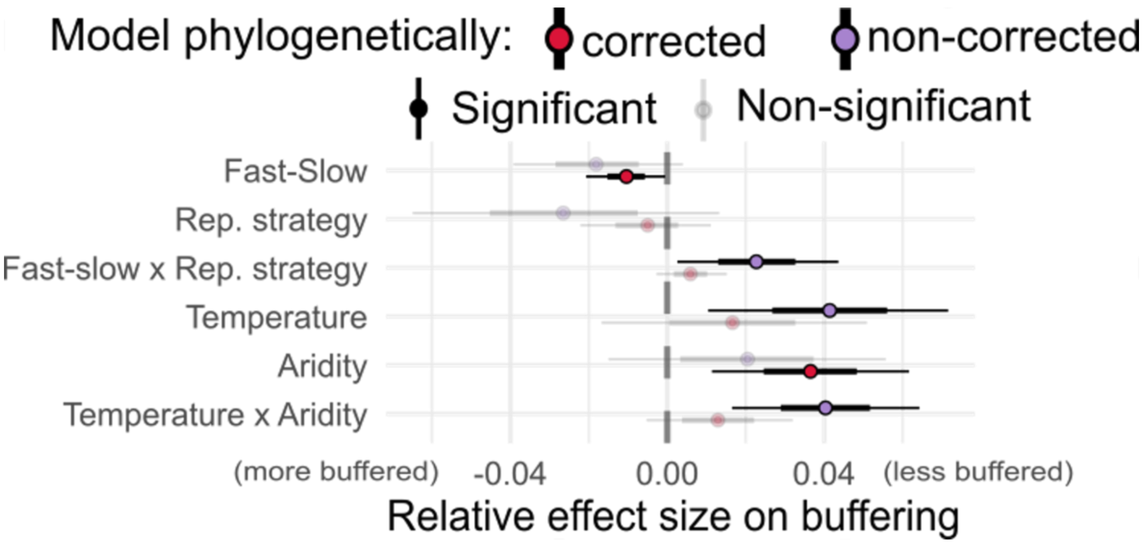

Figure 1. The position of our 121 plant populations along the buffering continuum – as quantified by the absolute value of the sum of stochastic elasticities of population growth rate to changes in the variance of all vital rates (*v*), $|\sum E_v^{\sigma}|$ – is mainly determined by environmental stochasticity in aridity. The figure shows the effect sizes (posterior distributions) of studied variables on $|\sum E_v^{\sigma}|$ obtained via MCMCglmm models with (red) and without (purple) phylogenetic corrections. Negative effect sizes indicate variables that promote demographic buffering. The models show that the contributions of reproductive strategies and temperature are only significant when phylogenetic relationships are not accounted for. The life-history axes are represented by $PC1_{LH}$ (fast–slow continuum) and $PC2_{LH}$ (reproductive-strategy continuum; see Fig. S2), while environmental stochasticity is summarised by $PC1_{Env}$ (variation in temperature) and $PC2_{Env}$ (variation in aridity; see Fig. S4).

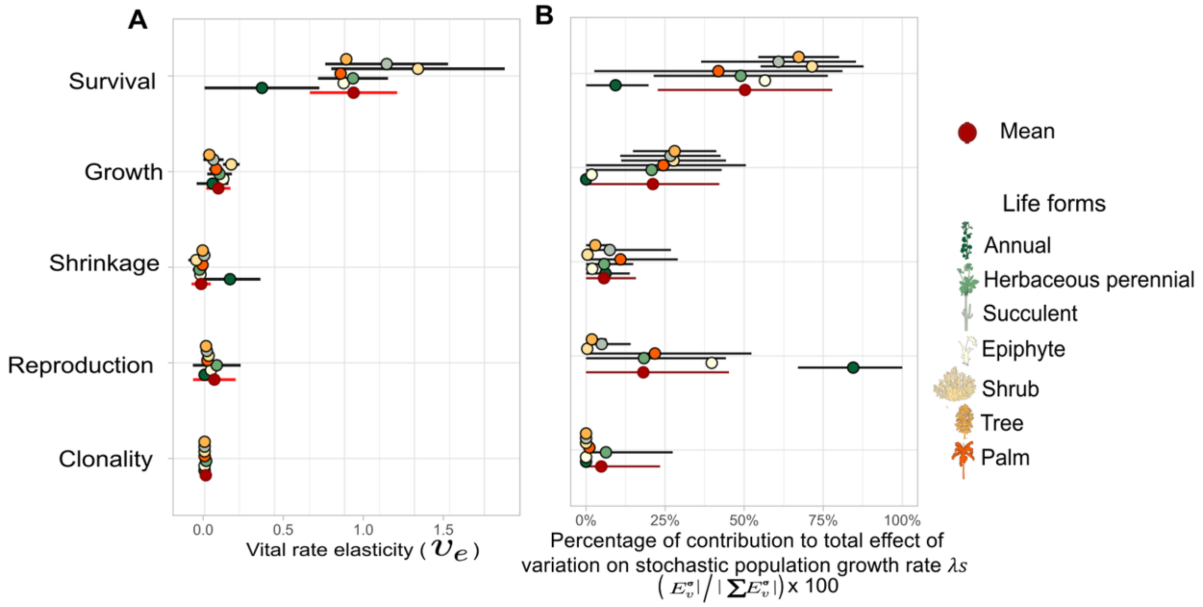

Figure 2. Temporal variance in survival is the primary driver of population responses to environmental stochasticity in most of our 121 plant populations, followed by individual-level growth and reproduction. **(A)** The proportional contribution of each vital rate (survival, growth, shrinkage, reproduction, clonality) to stochastic population growth rate λ_s quantified from the stochastic elasticities (E_v). **(B)** The proportional contribution of each vital rate to the populations' positions along the buffering continuum, as quantified by E_v^σ. Note that panel B is scaled to show proportional contributions as percentages, calculated as $\left[\frac{|E_v^\sigma|}{\sum |E_v^\sigma|}\right] \times 100$. Different life forms of plants are represented by different colours.

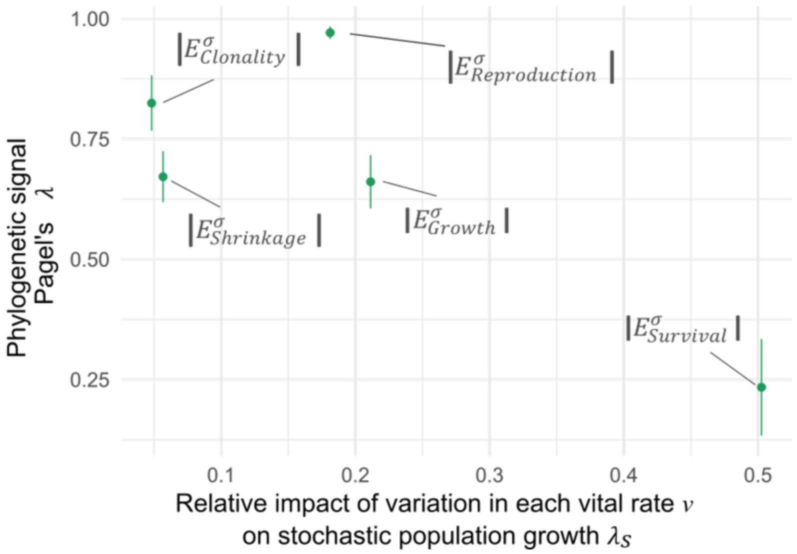

Figure 3. The vital rates that most drive buffering in plant populations are not necessarily those whose contributions are best predicted by evolutionary history. The figure shows

the absolute contribution of each vital rate *v* to populations' positions along the buffering continuum, $|E_v^\sigma|$, and its phylogenetic signal (Pagel's λ). Here, Pagel's λ quantifies how evolutionary history explains the contribution of each vital rate to demographic buffering. A phylogenetic signal closer to 1 indicates higher predictability by phylogeny. Contrary to our hypothesis (*H2*), the vital rates whose temporal variation contributed most to λ_s (i.e., $E_{Survival}^\sigma$) did not show a stronger phylogenetic signal.

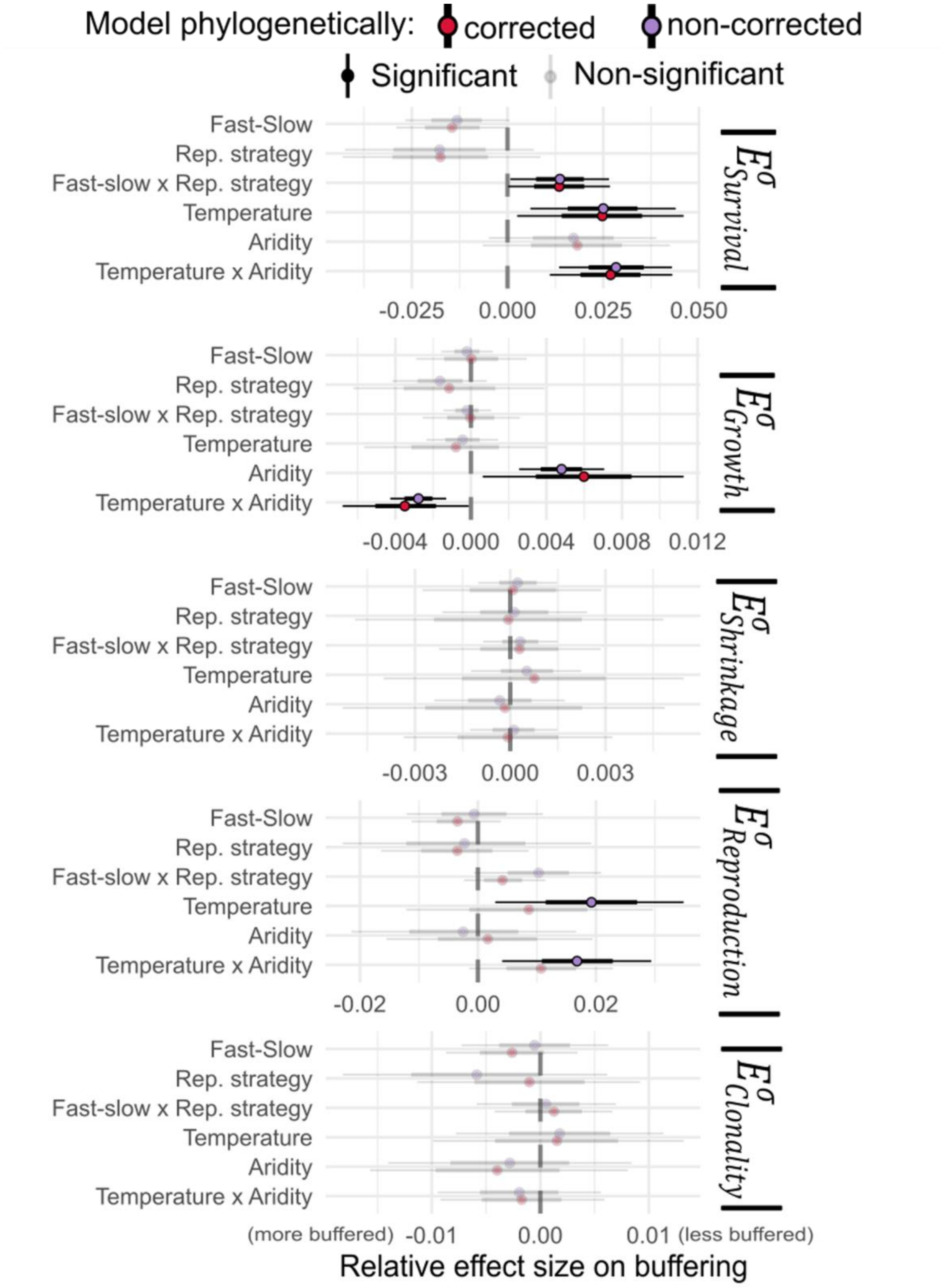

Figure 4. The effects of the contributions of life history strategies and environmental stochasticity to the position of our examined 121 plant populations along the buffering continuum vary greatly across vital rates ($|E^{\sigma}_{v}|$; *e.g.*, $|E^{\sigma}_{Survival}|$, $|E^{\sigma}_{Clonality}|$). MCMCglmm models with (red) and without (purple) phylogenetic corrections are presented. The life-history axes are represented by $PC1_{LH}$ (fast–slow continuum) and $PC2_{LH}$ (reproductive-strategy continuum; see Fig. S2), while environmental

stochasticity is summarised by $PC1_{Env}$ (variation in temperature) and $PC2_{Env}$ (variation in aridity; see Fig. S4).

- Models' outputs are now provided in the supplementary material (lines 852-864), thus helping those readers interested in further details of Figure 1 delve deeper. For those interested in the model output for each vital rate, the fully commented R scripts are also now provided as a long html report, where these outputs can be checked and tested for their reproducibility.

Lines: 864-874: *Table S3. The outputs of the models with and without phylogenetic corrections based on the most plausible model, after model selection. Effect size here means the extent to which the axes of life-history and environmental principal component analyses (PCAs) determine the position of our 121 plant populations along the buffering continuum, as quantified by* $|\sum E_v^\sigma|$. *Note that higher values of* $|\sum E_v^\sigma|$ *indicate a higher responsiveness of population growth rate (*λ_s*) to variation in vital rates. Thus, negative effect sizes indicate variables that help reduce the temporal variation in vital rates and consequently stabilise* λ_s*. Model outputs include posterior means estimated for each covariate (effect size) and its 95% credible interval (95% CI). We also show the effective sample size used to estimate the parameters (eff.samp) along the iterations in the Monte Carlo Markovian Chain and a p-value equivalence (pMCMC). Covariates are considered to significantly drive the buffering capacities of plant populations* $|\sum E_v^\sigma|$ *when the 95% CI do not cross zero (bold lines).*

		Effect size on $|\sum E_v^\sigma|$ (posterior mean)	95%CI	eff.samp	pMCMC
Phylogenetically corrected model					
	Intercept	0.068	[-0.276 - 0.410]	9900.000	0.676
	**Fast-slow ($PC1_{LH}$)**	**-0.011**	**[-0.022 - 0.000]**	**722.010**	**0.042**
	Rep. strategy ($PC2_{LH}$)	-0.006	[-0.024 - 0.013]	263.650	0.520
	Temperature ($PC1_{Env}$)	0.017	[-0.017 - 0.052]	1393.830	0.323
	**Aridity ($PC2_{Env}$)**	**0.036**	**[0.008 - 0.060]**	**1552.420**	**0.017**
	Fast-slow × Rep. strategy ($PC1_{LH} \times PC2_{LH}$)	0.007	[-0.004 - 0.017]	72.020	0.168
	Temperature × Aridity ($PC1_{Env} \times PC2_{Env}$)	0.014	[-0.008 - 0.035]	204.420	0.177
Non-phylogenetically corrected model					
	**Intercept**	**0.066**	**[0.030 - 0.102]**	**10176.00**	**0.001**
	Fast-slow ($PC1_{LH}$)	-0.018	[-0.004 - 0.004]	9900.00	0.105
	Rep. strategy ($PC2_{LH}$)	-0.026	[-0.065 - 0.015]	9900.00	0.204
	**Temperature ($PC1_{Env}$)**	**0.042**	**[0.012 - 0.072]**	**9571.00**	**0.007**
	Aridity ($PC2_{Env}$)	0.020	[-0.015 - 0.056]	9900.00	0.250
	**Fast-slow × Rep. strategy ($PC1_{LH} \times PC2_{LH}$)**	**0.023**	**[0.002 - 0.044]**	**9603.00**	**0.035**
	**Temperature × Aridity ($PC1_{Env} \times PC2_{Env}$)**	**0.041**	**[0.017 - 0.064]**	**9900.00**	**0.001**

We think these changes help to identify the key findings of our research.

The conclusion that animals do not align with the fast-slow continuum or reproductive strategies could be influenced by data limitations rather than reflecting a true biological pattern, especially given that only three years of empirical data were used as a filter for species inclusion. Additionally, the database contains a disproportionate number of plant species compared to animal species (78 plant species versus 11 animal species), which may skew the conclusions.

R5> We had been pondering our ability to talk about the generality of our findings in the animal kingdom prior to the submission of the first version, and indeed we are in a way relieved that this reviewer has brought it up independently. To address the challenge of small representativity in our animal dataset, after much careful thinking, we have decided to re-focus our manuscript on plants alone and restructured the manuscript accordingly. The limitation of animal data accessibility has been included in main text for transparency (lines *269-283*).

Lines 269-283: "A second area for improvement is the suboptimal representation of long-term demographic studies across taxonomies and geographies required for comparative demographic studies. The existing databases we use here, though state-of-the-art in regards of number of demographic studies, still remain biased toward perennial species in the Plant Kingdom [49,50]. For instance, while the robustness of survival as the main driver of buffering holds for most of our studied populations, annual plants may represent an alternative strategy for how survival-reproduction trade-off shape population performance on environmental stochasticity. However, the limited sample size for annual plants limited any inference pointing out the need for further studies. Similarly, given the small number of available data of animal populations, we failed to satisfactorily perform similar analyses for comparison. Altogether with the taxonomical covering, filling the demographic gap across continental scales, particularly South America and Africa, remains a pressing issue to gain a holistic understand of how natural populations respond to environmental stochasticity[49,50]. High-resolution demographic data, however, take by definition a long time to be produced, which makes filling this gap an enduring commitment by population ecologists in the coming decades.
"

We hope to return to this research question in the animal kingdom once COMADRE, PADRINO, and similar databases allow us to tackle its generality.

The figures are quite complex due to the multifactorial analyses. Including a schematic figure that summarizes the main results could help improve clarity for readers.

R6> To facilitate the understanding of our figures, we have:

- Moved Figure 1 to supplementary material – now Figure S1.

- Split Figure 3 to make the phylogenetic signal separate and easier to understand (Now Figures 3 and 4).
- Improved Figure 4 to make the contribution of each vital rate to $|\sum E_v^\sigma|$ easier to understand.
- New elements have been added to Figures 1 and 4 to highlight more and less buffering on the x axis.
- All figures have been simplified as there are no longer data on animals.

Please see R4 above for a detailed reply on how the figures have been improved. We think that this simplification enhances the readability and clarity of our figures. Moreover, we have also included in the Methods a more detailed explanation of what Equation 1 means and how the effect size can be interpreted (see the new lines 516-521).

Lines 516-521: "First, all covariates in our model were z-transformed ($\mu = 0$, and *sd* = 1). With this normalisation, we can compare the proportional contribution of each covariate to driving the population's position along the demographic buffering continuum using estimates of *β* (effect size). For instance, covariates showing a positive effect size indicate an increase in $|\sum E_v^\sigma|$, thus reducing buffering *First, it is important to note that all covariates in our model were z-transformed ($\mu = 0$, and sd = 1). With this normalisation, we can compare the proportional contribution of each covariate to driving the population's position along the demographic buffering continuum using estimates of β (effect size). For instance, covariates showing a positive effect size indicate an increase in $|\sum E_v^\sigma|$, thus reducing buffering.*"

Additionally, the Methods section lacks sufficient detail—specifically, the equation for the sum of stochastic elasticities with respect to variance is not fully described. I also found it unclear how the life history traits were derived from the data (line 429), and further clarification on this process would be beneficial.

R7> We thank the reviewer for raising this point. We have taken several steps to address this shortcoming in the previous version. Most importantly, the inclusion of a new step, the model selection, led us to considerably improve the subsection *Statistical analysis and the phylogenetic component*. The main changes in the Methods section include:

- A table in the supplementary material showing how each life-history trait was derived, and the pertinent references (see Lines 796-806).
- A new paragraph describing each covariate used in the model and how a final model was reached (Lines 507-521)
- We have corrected a few sentences that could lead to confusion and added a few more details in plain language to strengthen clear communication. For instance, the last line of the fourth paragraph in the methods now provided a nontechnical explanation to interpretate $\sum E_v^\sigma$ (see lines 338-352, particularly the bold sentence below).

Lines 796-806. **“*Table S1.*** *Formulation of the life-history traits used to assess life-history strategies in the 121 populations of 78 species of plants. Notations used here are: λ is the deterministic population growth rate, corresponding to the dominant eigenvalue of the matrix **A**[54]; l_x and m_x refer to the age-specific survivorship and fertility schedules, respectively, for each age x.* p_x *is the proportional contribution of reproduction weighted by the population trajectory* $p_x = \frac{l_x m_x \lambda^{\{-x\}}}{\sum_{x=0}^{xmax} l_y m_y \lambda^{\{-y\}}}$. *The xmax refers to the maximum age observed during the study period; **U** and **F** are the submatrices of survival- and fertility-dependent processes, respectively, whereby **A** = **F** + **U**; **U’** is the survival-independent matrix of transition probabilities[54,55]; **w** is the stable stage distribution of the matrix **A**, and i and j are the row and column entries of the matrix population model respectively. Finally, the R function refers to the command used to calculate the life history traits with Rage package v.1.6.0*[55]

Life history trait	Biological meaning	Formula	R function
Mean age at first reproduction (L_a)	Average amount of time from birth to reproduction	$L_a = \frac{\sum_{x=0}^{xmax} x l_x m_x}{\sum_{x=0}^{\max i} l_x m_x}$	*mature_age*
Probability of achieving reproduction before dying (p_α)	Probability of an individual become reproductive along their lifetime	$p_a = 1 - \prod_{x=0}^{xmax} (1 - l_x m_x)$	*mature_prob*
Mean life expectancy (η_e)	Expected lifetime of an individual at its birth	$e = \sum_{x=0}^{xmax} lx$	*life_expect_mean*
Reproductive window (*L*)	Temporal spread of reproduction throughout life span as quantified by Demetrius[82] entropy (*L*). High/low *L* values correspond to iteroparous/semelparous populations	$L = -\sum p_x \ln p_x$	*shape_surv*
Individual development (γ)	Mean probability of transitioning forward to a larger/more developed stage in the life cycle of the species, weighted by the stable stage distribution, w	$\gamma = \sum_1^m \overline{U'}_{i,j} \overline{w}_j \Big\|_{i<j}$	*vr_growth*

Distribution of mortality risk along the life cycle (*P*)	Shape of the age-specific survivorship curve l_x as quantified by Keyfitz' entropy[83]. *P* >1, =1, <1 correspond to species whose mortality hazards decrease, stay constant, or increase with age respectively.	$P = \frac{-\log(l_x)l_x}{\sum l_x}$	*shape_rep*

References: 1.Caswell, H. (John Wiley & Sons, Ltd: 2001); 2.Jones, O.R. et al. *Methods Ecol Evol* **13**, 770–781 (2022).; 3.Demetrius, L. *Proc. Natl. Acad. Sci. U.S.A.* **71**, 4645–4647 (1974). 4. De Vries, C., Bernard, C. & Salguero-Gómez, R. *Methods Ecol Evol* **14**, 1312–1319 (2023).

Lines 507-521: "*A detailed look at the biological meaning of the equation is important for better understanding methodological decisions and the resulting biological interpretation in inferential analyses. The equation yielded by the best-fitting model ($|\sum E_v^{\sigma}| = \alpha + \beta_{PC1_{LH}} \times \beta_{PC2_{LH}} + \beta_{PC1_{Env}} \times \beta_{PC2_{Env}}$) indicates that a population's buffering capacity against environmental stochasticity* (as quantified by $|\sum E_v^{\sigma}|$) *can be predicted from its position along the two principal life-history axes and the two main environmental axes experienced during the study period. Because PCA reduces the dimensionality of the dataset by transforming variables into new orthogonal axes, it is good practice to assign each component a clear context-specific interpretation that highlights its biological implications. Finally, two more technical details are necessary to be highlighted. First, all covariates in our model were z-transformed ($\mu = 0$, and sd = 1). With this normalisation, we can compare the proportional contribution of each covariate to driving the population's position along the demographic buffering continuum using estimates of β (effect size). For instance, covariates showing a positive effect size indicate an increase in $|\sum E_v^{\sigma}|$, thus reducing buffering. Second, we have distinguished between $\beta\lambda$ and β to refer to the slope estimated (i.e., the effect size) of models with and without phylogenetic corrections, respectively.*"

Lines 338-352 **(the bold sentence presents a nontechnical explanation to interpret $\sum E_v^{\sigma}$):** *The relative impact of variation in each vital rate v on stochastic population growth λ_s, can be assessed by combining two components: its deterministic elasticity (which reflects how much changes in v affect λ_s) and the temporal variability observed in v. When the observed variability in a vital rate is weighted by the vital rate's deterministic elasticity, the result is the stochastic elasticity with respect to the variance, E_v^{σ}, which represents the proportional contribution of variance in the vital rate to changes in λ_s. Summing all E_v^{σ} values results in $\sum E_v^{\sigma}$, which can be interpreted as the overall demographic impact of temporal variation in vital rates on λ_s[57,58].* ***In other words,***

$\sum E_v^\sigma$ represents how responsible variation in its vital rate v is for population performance. In the absence of variation in vital rates, or if the variation occurs only in those vital rates that contribute less to λ_s, we should find $\sum E_v^\sigma \approx \mathbf{0}$. On the other hand, even small variation in vital rates with greater contribution to λ_s is likely to increase the value of $\sum E_v^\sigma$ considerably.

Reviewer #2 (Remarks to the Author):

Manuscript 24-4911-T aims to examine the drivers of species' abilities to buffer against environmental stochasticity. This is an exciting idea with important implications especially under ongoing climate change. However, I do not think the analyses provide a robust test of the proposed idea. I have several large concerns about the interpretation of results that I detail below.

R8> We thank the reviewer for the insightful comments. We have re-analysed the data considering the comments about the Environmental PCA (see R10a,b and R11 below) and added a model selection step to address comments 2 and 3 (R11). These re-analyses did not change the general conclusions and improved the robustness of our results. Finally, there is only a minor point of disagreement regarding how the PCA axes should be presented throughout the manuscript. We have decided to keep the biological meanings (aridity and temperature) of the environmental PCA axes rather than the technical terms ($PC1_{Env}$ and $PC2_{Env}$), and we explain our reasoning for this choice in R10b.

Major concerns:

1. Many of the results compare animals and plants, (e.g., Lines 139-146,156-158), but the analyses include 121 populations belonging to 78 species of plants versus 13 populations depending to 11 species of animals. This makes for a very unequal sample size, and further making inferences for "animals" as a broad category based on 11 species is a huge over interpretation. I understand that this data is hard and time consuming to collect, and I appreciate the thorough cleaning of the data that removed many of the populations, but regardless have concerns about any inferences about animals broadly or comparisons between animals and plants given the sample size of animals.

R9> Data imbalance was indeed a general concern for all reviewers and we agree. Please, see the reply to this point in R5.

2. The inferences about the role of environmental stochasticity do not robustly isolate stochastic effects—the main framing of the manuscript—for two reasons.
2a. The first reason is that inferences are made based on the variance of each vital rate observed from the matrix population models, sum(E^\sigma_v). Low variances in observed population vital rates could arise due to small effects of environmental stochasticity, as the authors claim. However, low variances in population vital rates

could also arise due to small experienced environmental variation during the time series—even if the population is highly responsive to environmental stochasticity. If I am understanding correctly, this metric does not account for these two alternatives as currently presented, and thus cannot be used to examine sensitivity to environmental stochasticity generally. As a side note, time series length across the different data sets could also alter sum(E^\sigma_v), as longer term time series are more likely statistically to have experienced an extreme event drawing from the tail of the environmental distribution.

R10> We thank the reviewer for this very interesting comment. We have split this comment into two distinct parts, so we can address each concern properly. Part 1 below discusses whether $\sum E_v^\sigma$ differentiates between environmental stochasticity and 'population responsivity'. Part 2 then discusses whether the term $\sum E_v^\sigma$ might be biased by the time series of each study.

Part 1: The sum of stochastic elasticity with respect to variance, $\sum E_v^\sigma$, evaluates how populations respond to variation in their vital rates, rather than to environmental stochasticity. Thus, the reviewer is absolutely correct that no distinction can be made between the stochasticity experienced by populations and their sensitivity to stochasticity. This is precisely why the environmental PCA in our GLMM is crucial**.** By including a metric of stochasticity in our GLMM, we gain a comparative perspective on the factors driving species' responses to environmental stochasticity. It is worth noting that previous assessments of the buffering continuum in natural populations did not include environmental stochasticity (*e.g.*, Morris et al. 2008, McDonald et al. 2017), which makes our study unique in this sense. Most importantly, by including the environmental variables in our MCMCglmm models, we successfully capture the role of environmental stochasticity and we can then also compare its effect size to the life-history axes and phylogenetic signal. Based on this novel approach and set of results, we can thus support our hypothesis H_3, which states that the contribution of the fast-slow continuum to $|\sum E_v^\sigma|$ may be inflated when environmental stochasticity is not accounted for.

Please note that we have rewritten H3 to clarify this point – see new lines 96-104.

Lines 96-104: "*Finally, none of the existing studies exploring demographic buffering have explicitly considered the amount of environmental stochasticity faced by the studied population. This omission likely inflates the perceived roles of life history traits and phylogenetic relationships in explaining population responses to environmental variability, which leads us to hypothesise (H3) that the extent to which a natural population displays demographic buffering strongly depends on its environmental regime. Specifically, higher stochasticity in precipitation and temperature is expected to lead to greater variances in vital rates, shifting populations toward a less buffered end of the demographic buffering continuum.*

On a side note, just for further clarification, we do not claim that species with higher values of $\sum E_v^\sigma$ are more or less sensitive to environmental stochasticity. Instead, our

main claims are: 1) life history traits predict the buffering capacity of natural populations, which is supported by a negative relationship between our life history PCA and $|\sum E_v^\sigma|$, particularly $PC1_{LH}$; and 2) as environmental stochasticity increases, the buffering capacity of our 121 examined populations decreases, which is supported by a positive relationship between $PC2_{Env}$ and $\sum E_v^\sigma$. One could understand this negative relationship between $PC1_{LH}$ and $|\sum E_v^\sigma|$ as long-lived species being less reactive to environmental stochasticity. Although we think this rationale is likely to hold, this should be better supported if our best model included interactions between life history and environmental PCAs (*e.g.*, $PC1_{LH} \times PC1_{Env}$ – it means, Fast-slow × Seasonal aridity). For instance, a positive relationship between 'Fast-slow × Seasonal aridity' and $|\sum E_v^\sigma|$ are likely to inform that fast life histories amplify the effect of season aridity on stochastic population growth rate λ_s. Although it will be worth exploring in depth these interactions between life history and environmental PCAs, they were not included in the best model reached by our model selection approach (see R11). Therefore, we decided not to discuss these interactions further.

Part 2: The reviewer raised an interesting point regarding the length of the time series. As the reviewer suggests, 'longer time series are more likely statistically to have experienced an extreme event", with which we agree. However, we have now tested whether $|\sum E_v^\sigma|$ is influenced by the time series and we find no relationship (see Figure R10). In addition, we discuss that the duration of the time series should be of minor concern in our study because $\sum E_v^\sigma$ is calculated iteratively and our GLMM already accounts for environmental stochasticity. Note this analysis and its rationale were not included in the main manuscript, as the effect of time series length on $\sum E_v^\sigma$ has not yet been addressed in the literature.

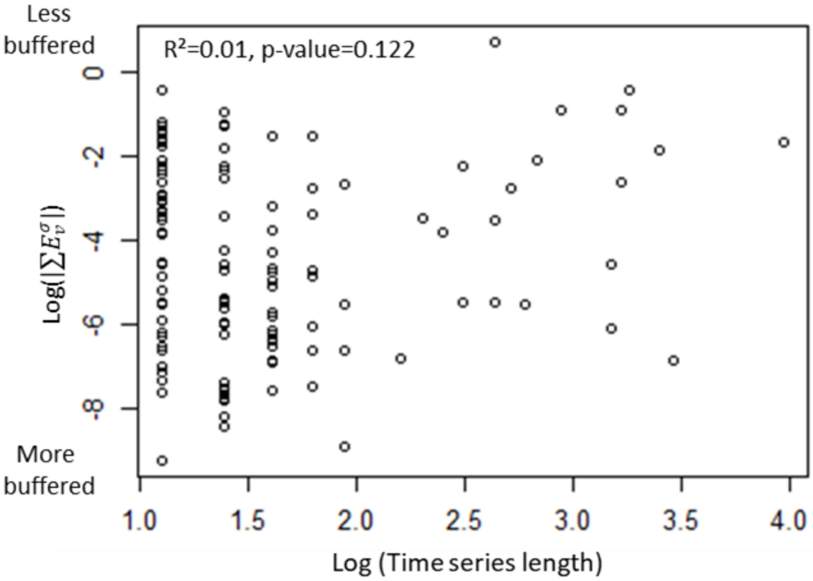

Figure R10. There is no significant relationship between the study duration of the examined 121 plant populations and their position along the continuum quantified as the sum of stochastic elasticity with respect to variance - $\sum E_v^\sigma$. Both axes were log-transformed and the resulting R^2 and p-value from a simple linear model are shown. Figure and analysis are provided in the HTML supplementary material as a separate analysis

The absence of relationship between $\sum E_v^\sigma$ and study duration is likely a result of the way $\sum E_v^\sigma$ is calculated, which involves randomising vital rates as independent and identically distributed events. This randomisation process is likely to reduce the influence of individual extreme events in longer time series and counterbalances the extreme events with a greater number of years closer to the mean. We have included the information about the randomising process in the Methods, see new lines 322-325:

Lines 329-332: "*In mathematical terms, stochastic elasticity is the partial derivative of the vital rates v (or matrix elements, denoted by* e_{ij}^s*) over the MPM time series weighted by the relative contribution to* λ_s*, and is typically expressed as* $e_{ij}^s = (a_{ij} \times \partial\lambda_s)/(\lambda_s \times \partial a_{ij})$*. Thus,* e_{ij}^s *can be calculated iteratively based on how observed differences in* a_{ij} *affected* λ_s [51]"

In addition to the randomisation process, it is important to recognise that environmental stochasticity is already represented by our Environmental PCA, which was built based on monthly temperature and precipitation during each study. Therefore, any extreme events experienced by populations during the study period are likely to be captured by these climatic variables and are explicitly incorporated into our analyses. For instance, extreme events in the climatic variables are likely to increase the proportional environmental stochasticity (R^{CV}), calculated for monthly maximum and minimum temperature and precipitation. Yet, we agree that $\sum E_v^\sigma$ reflects only the observed environmental stochasticity. While this might seem like a limitation for predicting population responses under future scenarios of extreme events, our manuscript focuses solely on response to observed environmental stochasticity. That said, study duration should not be a major concern in our study.

REFERENCE

Morris WF, Pfister CA, Tuljapurkar S, Haridas CV, Boggs CL, Boyce MS, Bruna EM, Church DR, Coulson T, Doak DF, Forsyth S, Gaillard J-M, Horvitz CC, Kalisz S, Kendall BE, Knight TM, Lee CT, Menges ES. 2008. Longevity can buffer plant and animal populations against changing climatic variability. *Ecology* 89:19–25. DOI: 10.1890/07-0774.1.

McDonald JL, Franco M, Townley S, Ezard THG, Jelbert K, Hodgson DJ. 2017. Divergent demographic strategies of plants in variable environments. *Nature Ecology & Evolution* 1:0029. DOI: 10.1038/s41559-016-0029.

2b. The manuscript uses PC1Env and PC2Env to examine effects of the environment, claiming that PC1Env describes environmental stochasticity while PC2Env describes seasonal aridity. While I do not have any concerns about this approach generally, I have concerns about the interpretation. 6 environmental variables went into the PCA analyses, including many effects unrelated to environmental stochasticity, such as mean value of the maximum temperature, relative amplitude of seasonality in minimum temperature etc. Therefore, even though PC1Env largely correlates with CV of maximum temperature, it does not directly quantify environmental stochasticity, making claims

about the effects of environmental stochasticity based on PC1Env fraught. Further, the claim (line 480) "The first two PCs emerging from this next step explained 50.9% of the variation in environmental stochasticity" is false, given that other components of the environment that are not part of stochasticity (mean value of maximum temperature) were included in the PCA analysis. Similarly, but a smaller concern, is referring to PC2Env as seasonal aridity throughout the manuscript, since aridity could itself be directly measured. Rather, I suggest referring to this as PC2Env and just stating that PC2 largely correlates with the aridity gradient.

R10b> We thank the reviewer for giving us the opportunity to clarify a few points regarding our environmental PCA, and for highlighting areas where the manuscript could be improved. First, we have carefully considered this comment and we agree that selecting the number of axes based only on the total explanatory variance was not a satisfactory approach. So, we have applied a more conventional approach, which includes all axes with eigenvalues greater than 1 in the analyses following the Kaiser criterion Legendre & Legendre (2012). This widely used practice led us to include a third axis of the environmental PCA in our analyses (model selection). This new axis is closely related to the amplitude of maximum temperature ($\Delta T_{TempMax}$) and explains 17.5% of the environmental variability, resulting in a cumulative of total variance explained by PCA of 68.2%. However, because we ended up with a new variable for which we had made no predictions in the earlier version of the manuscript, we decided to include a model selection step to inform what is the most plausible model to determine buffering capacity. By including this model selection step, we robustly select the variables that are more likely to explain $|\sum E_v^\sigma|$. Model selection was performed using Deviance Information Criteria and the best model that included was the same model we have used. Including a third axis dramatically increased the number of parameters in our model (df = 4) and reduced its plausibility (ΔDIC = -4.17). By including a model selection step that confirms that we already used the most plausible model, a new layer of robustness was included in our analyses. Please, see R11 where we discuss in depth the validity of our model.

Moreover, we also thank the reviewer for the opportunity to clarify that mean temperature was only used to decompose its timeseries in trend, seasonality, and noise (stochasticity), thus $R_{TempMax}^{CV}$ is not the coefficient of variation in maximum temperature but rather the coefficient of variation of the noise in this variable (the component not explained by trend and seasonality). In addition, we could not remove the mean trend ($\underline{T}_{Temp}$) from our PCA because this variable is likely to reflect the geographic position of the populations (tropical *vs.* subtropical areas). The same time series decomposition was applied to precipitation. Thus, even if we could quantify seasonal aridity directly or use other sources such as the WordClim raster (*e.g.,* BIO15 = Precipitation Seasonality - https://www.worldclim.org/data/bioclim.html), we risk not being able to match the environmental conditions that the populations experienced during the study. Secondly, it may not be directly comparable to the effect of temperature.

Finally, we politely disagree on how to present the PCA axes to the readers. Given the broad readership of *Nature Communications,* presenting the biological meaning of PCAs

instead of their technical terms is essential to make the main message clearer. This practice has been widely used in high-impact journals with great appeal to a large audience, particularly on defining life history axes such as fast-slow continuum, reproductive strategies (*e.g.*, Salguero-Gomez et al. 2015, 2016; Diaz et al. 2015). The same approach was extended to the environmental variables in our study. We agree that it may not sound the most technically accurate approach to refer to these PCAs axes, but we are convinced that it dramatically improves comprehension, so its pay-off. Moreover, for those interested in a more in-depth understanding of each axis of the PCAs, a detailed supplementary material is provided showing the relationship of each climatic variable with the environmental PCA.

REFERENCES

Legendre, P. & Legendre, L. 2012. Numerical Ecology. (Elsevier, Amsterdam Boston).

Salguero-Gómez R. 2017. Applications of the fast–slow continuum and reproductive strategy framework of plant life histories. *New Phytologist* 213:1618–1624. DOI: 10.1111/nph.14289.

Salguero-Gómez R, Jones OR, Jongejans E, Blomberg SP, Hodgson DJ, Mbeau-Ache C, Zuidema PA, de Kroon H, Buckley YM. 2016. Fast–slow continuum and reproductive strategies structure plant life-history variation worldwide. *Proceedings of the National Academy of Sciences* 113:230–235. DOI: 10.1073/pnas.1506215112.

Díaz S, Kattge J, Cornelissen JHC, Wright IJ, Lavorel S, Dray S, Reu B, Kleyer M, Wirth C, Colin Prentice I, Garnier E, Bönisch G, Westoby M, Poorter H, Reich PB, Moles AT, Dickie J, Gillison AN, Zanne AE, Chave J, Joseph Wright S, Sheremet'ev SN, Jactel H, Baraloto C, Cerabolini B, Pierce S, Shipley B, Kirkup D, Casanoves F, Joswig JS, Günther A, Falczuk V, Rüger N, Mahecha MD, Gorné LD. 2016. The global spectrum of plant form and function. *Nature* 529:167–171. DOI: 10.1038/nature16489.

3. I think it is important to examine life history strategy interactions with environment variables (Figures 3, 4 and corresponding analyses), as the fast-slow strategy might greatly influence species' buffering capacities in highly variable environments, but less so in less variable environments. I can easily envision interactive effects with aridity/PC2Env as well.

R11> Initially, we decided to provide a more concise analysis of the effect of the environmental variables on $|\sum E_v^\sigma|$. However, we agree and have realised that there are several potential models that could inform it better. Therefore, we have decided to include an additional model selection step to compare multiple competing alternative models that could inform $|\sum E_v^\sigma|$. This new step ended up supporting our initial model ($|\sum E_v^\sigma| = \alpha + \beta_{PC1_{LH}} \times \beta_{PC2_{LH}} + \beta_{PC1_{Env}} \times \beta_{PC2_{Env}}$) as the most plausible model.

Finally, including the model selection as a new step did not alter the conclusions we had drawn using the MCMCglmm package; instead, this step has provided a much stronger

rationale for the analyses, which also improves the readability of the manuscript. Please see the first two paragraphs in the *Statistical analysis and the phylogenetic component* section (lines 497-541) to see the changes that have been made in this regard.

Please see the competing models and their performance in the new lines 887-886.

“Table S2. Competing explanatory models for demographic buffering in plants $|\sum E_v^\sigma|$*. Models are informed according to degrees of freedom (df) and Deviance Information Criterion (DIC). Models are ranked from lowest to highest DIC. Model with lowest DIC was used in posterior MCMCglmm analyses. Variables included in model selection were those axes with eigenvalues greater than 1:* $PC1_{LH}$ (fast–slow continuum) and $PC2_{LH}$ (reproductive-strategy continuum; see Fig. S2) from the life history PCA. From the environmental PCA we used the $PC1_{Env}$ (variation in temperature), $PC2_{Env}$ (variation in aridity), and $PC3_{Env}$ (amplitude of temperature)*. Two null models were considered: a standard null model, where none of the variables are informative – named Null model, and a second null model where climatic information is no informative – named Climatic null model).*

Models	*df*	*DIC*
$|\sum E_v^\sigma| \sim PC1_{LH} \times PC2_{LH} + PC1_{Env} \times PC2_{Env}$	*9*	*-400.7529*
$|\sum E_v^\sigma| \sim PC1_{LH} \times PC2_{LH} + PC1_{Env} \times PC2_{Env} \times PC3_{Env}$	*13*	*-396.5775*
$|\sum E_v^\sigma| \sim 1$ *(Null model)*	*3*	*-384.2591*
$|\sum E_v^\sigma| \sim PC1_{LH} \times PC2_{LH} + PC3_{Env}$	*7*	*-379.2992*
$|\sum E_v^\sigma| \sim PC1_{LH} \times PC2_{LH} + PC2_{Env}$	*7*	*-376.4730*
$|\sum E_v^\sigma| \sim PC1_{LH} \times PC2_{LH} + PC2_{Env} \times PC3_{Env}$	*9*	*-376.4730*
$|\sum E_v^\sigma| \sim PC1_{LH} \times PC2_{LH} + PC1_{Env} + PC2_{Env}$	*7*	*-376.1849*
$|\sum E_v^\sigma| \sim PC1_{LH} \times PC2_{LH} + PC1_{Env} + PC2_{Env}$	*8*	*-373.1743*
$|\sum E_v^\sigma| \sim PC1_{LH} \times PC2_{LH} + PC1_{Env} + PC2_{Env} + PC3_{Env}$	*9*	*-372.6623*
$|\sum E_v^\sigma| \sim PC1_{LH} \times PC2_{LH} + PC1_{Env} \times PC3_{Env}$	*9*	*-364.3317*
$|\sum E_v^\sigma| \sim PC1_{LH} \times PC2_{LH}$ *(Climatic null model)*	*6*	*-342.2609*
$|\sum E_v^\sigma| \sim PC1_{LH} \times PC2_{LH} + PC1_{Env}$	*7*	*-340.1330*
$|\sum E_v^\sigma| \sim PC1_{LH} \times PC2_{LH} \times PC1_{Env} \times PC2_{Env} \times PC3_{Env}$	*34*	*-331.3077*
$|\sum E_v^\sigma| \sim PC1_{LH} \times PC2_{LH} \times PC1_{Env} \times PC2_{Env}$	*18*	*-257.7108*

Minor Comments:

1. Lines 151-153. Would an alternative interpretation be that the reproductive strategy continuum is important for populations’ responses, but that reproductive strategy is largely phylogenetically conserved?

R12> We thank the reviewer for this suggestion, and the opportunity to clarify this point. In our study, the inclusion of the phylogenetic analysis serves a dual purpose: it discounts the impact of other considered traits and emphasises the evolutionary significance of demographic patterns. This comment shows us that we could provide a more detailed explanation of the role of phylogenetic signal in demographic buffering theory, particularly with regard to its influence on buffering patterns. In the revision, we have made extensive changes to the explanation of the phylogenetic signal, including better contextualisation as a new separate hypothesis (new H2), see below.

First, to clarify potential confusion, we need to remind the reviewer/readership that high values of E_v^σ mean that variation in this vital rate has a greater effect on the stochastic population growth rate (λ_s). Thus, in our case, variation in reproduction ($E_{Reproduction}^\sigma$) can be interpreted as contributing much less to λ_s than variation in survival ($E_{Survival}^\sigma$). However, this result differs for our estimates of Pagel's λ, which indicates the phylogenetic conservatism of a given trait. Indeed, $E_{Reproduction}^\sigma$ shows a higher Pagel's λ compared to $E_{Survival}^\sigma$. Altogether, this result can be interpreted as the total effect of the vital rate on the population's response to environmental stochasticity (measured as $|\sum E_v^\sigma|$) being more predictable by phylogeny than the contribution of $E_{Survival}^\sigma$ to $|\sum E_v^\sigma|$.

We now provided a better explanation of the phylogenetic signal, including a better contextualisation to what is now our new H2 (see lines 92-96) which will avoid any further confusion.

Lines 92-96: "*..., it is challenging to anticipate a general pattern of temporal variation across all vital rates, thus blurring our current understanding of buffering in organisms. To better understand the contribution of vital rates on demographic buffering, we test (H_2) whether the phylogenetic signal of the buffering capacities of the examined species is stronger in the vital rates that contribute most to λ_s, since demographic buffering is a result of natural selection*[19,28]"

We have also substantially rewritten part of the results section to clarify the role of phylogeny in demographic buffering theory. See below.

Lines 142-158: "*It is important to consider that each vital rate contributes differently to demographic buffering and presents a different degree of phylogenetic conservatism. Indeed, the position of populations along the demographic buffering continuum is mainly determined by the temporal variance of survival ($E_{Survival}^\sigma$ = 50.26 ± 27.60%; Figure 2b), followed by individual-level growth (E_{Growth}^σ = 21.13± 21.01%) and reproduction ($E_{Reproduction}^\sigma$ = 18.12± 27.05%), with minor contributions from individual-level shrinkage ($E_{Shrinkage}^\sigma$ = 5.67 ± 10.12 %) and clonality ($E_{Clonality}^\sigma$ = 4.81± 18.57 %; Figure 2b and Table S5). However, contrary to our expectations (H_2), we did not find a positive relationship between the vital rates that contribute most to this buffering capacity and their phylogenetic signal (r=-0.7, S=34, p-value=0.230). Indeed, survival, the vital rate that contributes most to buffering, shows the weakest phylogenetic signal (Pagel's λ of $E_{Survival}^\sigma$ = 0.240; Figure 3). On the other hand, the contributions of temporal variation in sexual reproduction and clonality to λ_s ($E_{Reproduction}^\sigma$ and $E_{Clonality}^\sigma$) are highly*

phylogenetically conserved, as revealed by the strongest phylogenetic signal (Pagel's λ of $E^{\sigma}_{Reproduction}$= 0.972 and Pagel's λ of $E^{\sigma}_{Clonality}$ = 0.823, respectively; Figure 3). This high phylogenetic signal is also evident in the GLMM models, particularly for reproduction, where the contribution of the fast-slow continuum ($PC1_{LH}$) on determining $E^{\sigma}_{Reproduction}$ disappears when phylogenetic relationships are accounted for in our analyses (see Figure 4d)

2. Lines 121-125, Figure 2 and Table S1. The results discuss the average effect across animals versus plants. Is this from a weighted average based on coverage in the database? This general statement doesn't hold for many of the taxa, especially for the statement that individual-level growth comes second in terms of importance (e.g. reproduction is key for arthropoda, and annual plants).

R13> We used a simple average to draw our conclusions. In our analyses, the contribution of vital rates to buffering varies among different life forms and taxa, but survival is by far the most important vital rate for most of them. Other vital rates such as growth and reproduction alternate as the second most important, and only for annual plants (also for Arthropoda - but animals have been removed) reproduction exceeded survival. These few exceptions, however, offer limited conclusions because they are based on a reduced number of populations. For instance, we only have four populations of annual plants, each of a different species. Due to this small sample size, we decided to avoid an in-depth discussion of taxa/life-form inferences, which in turn will no doubt open new avenues for investigation in the near future once we have acquired a better representation of demographic data across taxa and growth forms. However, to improve transparency, we have added a few inserts in results and discussion to address the difference presented by annual species. In brief we now (i) suggest that annual plants may represent an alternative strategy for how survival-reproduction trade-off shape population performance on environmental stochasticity; however, due to limited annual sample size, we could not offer a robust inference in this regard (see lines 270-276); (ii) highlight the high phylogenetic conservatism might be particularly important to consider on modelling annual plant species (lines 245-253).

Lines 245-253 **(new insert in bold)**: "Additionally, we suggest that because the effects of reproduction on population responses to environmental stochasticity are highly predictable by evolutionary history, there is an opportunity to improve the parameterisation and accuracy of demographic models in population viability analyses[47]. **This opportunity includes models examining early impacts of exploitation, such as seed harvesting[48], and might be particularly important for modelling annual plants, as reproduction is particularly important to drive their population response to environmental stochasticity**. Moreover, when survival, the most important and less predictable vital rate, cannot be fitted in model parameterization, reducing uncertainty in other vital rates, such as reproduction, is imperative[47]. Our findings show a promising avenue for research in this direction.'

Lines 270-276: "The existing databases we use here, though state-of-the-art in regards of number of demographic studies, remain biased toward perennial species in the Plant

Kingdom [49,50]. For instance, while the robustness of survival as the main driver of buffering holds for most of our studied populations, annual plants may represent an alternative strategy for how survival-reproduction trade-off shapes population performance on environmental stochasticity. However, the limited sample size for annual plants prevented any inference pointing out the need for further studies."

3. Results: referring to figure subpanels would be helpful.

R14> Done.

4. Figure 2: For annual plants, is seed banking ability included in the models? If not, the extreme result for reproduction is unsurprising. It is not clear to me how seedbanking would fit into the vital rates, but is a critical aspect to include for analyzing annual plants.

R15> We agree that the inclusion of seedbanks in annual plant species models is important. This is an area of research where some of my coauthors have published quite a bit, and where the emerging message is that unfortunately more explicit work below ground needs to be done (Nguyen et al.2019; Bucharova et al. 2023). All annual populations in our study included seed bank dynamics in their models; however, because we only have four populations of four different species of annual plant, no robust inferences can be made.

References:

Nguyen V, Buckley YM, Salguero-Gómez R, Wardle GM. 2019. Consequences of neglecting cryptic life stages from demographic models. *Ecological Modelling* 408:108723. DOI: 10.1016/j.ecolmodel.2019.108723.

Bucharova A, Bossdorf O, Scheepens JF, Salguero-Gómez R. 2023. Sustainability of seed harvesting in wild plant populations: an insight from a global database of matrix population models. DOI: 10.1101/2023.01.12.523821.

5. Figures 3 and 4: Suggest changing "Estimated effect size" to "Effect on sum(E^\sigma_v)" or something similar so the reader knows what the response variable of interest is.

R16> Done. Please, see Figures 1 and 4, and R4. We have also improved the clarity of the figures by providing the following: 1) better clarification in their legends (see below in bold); and 2) a note indicating that a negative effect size in Figures 1 and 4 means variables promoting buffering.

Figure 1 legend (see R4). "**Figure 1.** The position of our 121 plant populations along the buffering continuum – as quantified by the absolute value of the sum of stochastic elasticities of population growth rate to changes in the variance of all vital rates (v), $|\sum E_v^\sigma|$ – is mainly determined by environmental stochasticity in aridity. The figure shows the effect sizes (posterior distributions) of studied variables on $|\sum E_v^\sigma|$ obtained via

MCMCglmm models with (red) and without (purple) phylogenetic corrections. **Negative effect sizes indicate variables that promote demographic buffering**. The models show that the contributions of reproductive strategies and temperature are only significant when phylogenetic relationships are not accounted for. The life-history axes are represented by $PC1_{LH}$ (fast–slow continuum) and $PC2_{LH}$ (reproductive-strategy continuum; see Fig. S2), while environmental stochasticity is summarised by $PC1_{Env}$ (variation in temperature) and $PC2_{Env}$ (variation in aridity; see Fig. S4).

6. Ln 266: Define the demographic liability hypothesis.

R17:> Done:

Lines 256-259 *"First, demographic buffering and its counterpart, the demographic lability hypothesis—which proposes that natural selection favours temporal variation in the most influential vital rates*[19,21,48] *— both rest on evolutionary mechanisms that have never been directly tested."*

Reviewer #3 (Remarks to the Author):

This manuscript represents a sizable contribution to eco-evolutionary theory by putting commonly held notions on life-history continuums into an appropriate environmentally-influenced and phylogenetically corrected-for context. I found the paper and it's findings genuinely interesting and I think this will be a well-citated piece of work. My main comments are related to the structure of some sections, in that the wording can be a bit opaque or indirect in places and so it can take several read-through's to fully grasp what the authors are saying. I hope my comments are helpful in this respect.

R18:> We are grateful for this reviewer's positive appraisal of our work. We have carefully addressed all their comments below.

Specific comments
Line 70-80: it's not super clear to me why one would 'expect' H1b, given the authors have set up why these two continua should be related to demographic buffering in the previous paragraph. I get that there are caveats (eg Jensen's inequality) but the text doesn't fully set up why we would expect this to be the case as a hypothesis. This is made clearer below, but I just feel like there is something off with the structuring of this paragraph of the intro such that the progression of ideas isn't super clear, and since it's a key point of the paper I think it just needs a slight bit of reorganization.

R19> We thank the reviewer for highlighting the 'lack of flow' in this specific section of the introduction. Following this and feedback from reviewer #1 (see R3 above), we have thoroughly revised the introduction to make the text clearer.

What we argue (and provide evidence for, see lines 61-78 below) is that the fast-slow continuum and the reproductive strategies continuum have been strongly related to

demographic buffering by previous researchers because environmental stochasticity and evolutionary history are not explicitly considered. Moreover, previous attempts to understand demographic buffering have often failed to consider different reaction norms and differences in vital rates (see Koons et al. 2006 and Lawson et al. 2015). In the context of accounting for these key omissions, variation in vital rates should be minimised, in general.

Please note that the new version of this paragraph includes a clearer explanation of Jensen's inequality, as well as a clearer contextualisation of life history continuums and buffering capacity. Here we highlighted in bold the sentences where these points have been clarified.

Lines 61-80: "So far, the link between life history continua and demographic buffering remains largely untapped for multiple reasons. First, because we are still lacking a general understanding of how each vital rate drives populations' response to environmental stochasticity. Second, the demographic buffering hypothesis[17,19] often assumes linear or concave (∩-shaped) shapes of environment-vital rate reaction norms[20–22], which indeed tends to result in negative effects on long-term of stochastic population growth (λ_s) when environmental variation increases. **Alternatively, convex (∪-shaped) reaction norms may represent an entirely different outcome, yet this possibility remains largely overlooked in demographic buffering analyses[21]. In convex (∪-shaped) vital rate reaction norms, environmental variability may amplify the beneficial outcomes of higher values of the vital rate, thus outweighing the detrimental effects of lower values[21,24] via the so-called Jensen's inequality[23]**. In other words, environmental variability is beneficial when the mean stochastic population growth rate, λ_s, is higher than deterministic population growth rate, λ. Importantly, there are limits to the amount of variance that a vital rate can exhibit without driving a population locally extinct[25]. **Third, and equally important, environmental stochasticity has, to date, not been explicitly considered in studies of demographic buffering. Consequently, populations that show reduced temporal variability during the study time frame are prone to being interpreted as buffered, regardless of whether they have been exposed to high or low environmental stochasticity**. Altogether, these complementary parts of the demographic buffering hypothesis remain a major challenge in linking life history continua to populations' response to environmental stochasticity in the context of climate change."

Lawson, C. R., Vindenes, Y., Bailey, L. & van de Pol, M. Environmental variation and population responses to global change. *Ecol Lett* **18**, 724–736 (2015).

Koons, D. N., Pavard, S., Baudisch, A. & Jessica E. Metcalf, C. Is life-history buffering or lability adaptive in stochastic environments? *Oikos* **118**, 972–980 (2009).

Line 125 – This may show my animal-bias, but I don't fully understand shrinkage and how traits related to shrinkage are not the same as those related to growth/survival.

The methods section also lists it but doesn't really describe it. Again, given that it is a key finding, I think laying out explicitly what shrinkage is and what it means would be really helpful for the reader.

R20> Thank you for raising this concern and give us the opportunity for clarification.

- First, note that we have removed animals from our analyses – please see R5 above.
- Second, we realised that shrinkage was indeed poor explained in the manuscript. This new version provides a more detailed description of shrinkage, including a clear distinction between growth and shrinkage (see lines 312-321).
    - Lines 312-321: "*Each matrix element a_{ij} in a **A** represents the contribution of a current (st)age to the next (st)age via survival, sexual and clonal reproduction, and development of individuals*[51,54]. ***Development, in turn, can be decomposed into two processes: growth (development/progression to a larger or more advanced stage) and shrinkage (retrogression to a smaller or earlier stage)***. *In total, five vital rates are entangled in matrix **A:** survival, growth, shrinkage, reproduction, and clonality. To disentangle each vital rate, these **A** matrices should be decomposed into submatrices representing the different processes of the life cycle: the **U** submatrix describes survival-dependent transitions (e.g., progression, retrogression), while the **F** and **C** submatrices describe sexual and clonal reproductions, respectively*[52]."
- Finally, if we may, we would like to provide some internal clarity to the reviewer: shrinkage in animals happens rather frequently among corals and sponges (and other modular organisms, *e.g.*, Lasker 1990). More generally, retrogression (which is a more comprehensive demographic process that includes shrinkage and de-development; Caswell 2001) occurs frequently among animals like birds and some mammals, who can oscillate between non-reproductive after reproduction stages. In our dataset, retrogression was represented by very few populations such as the polar bear (*Ursus maritimus; Hunter et al. 2010 see below*). Shrinkage/retrogression can be particularly important for species with high parental care, where mothers can take a 'sabbatical year' (or more), during which time they provide care for their offspring or recover from the high reproductive investment, or for species that undergo diapause/hibernation/dormancy.

See the life-cycle provided in Hunter et al. 2010 (below) for illustration. Note that the fourth stage does not contribute directly to recruitment (which is two-year old individuals in stage 1). Thus, two retrogression events are possible in this life-cycle: from stage 5 to 4 and from 6 to 4.

PUBLISHED DATA FIGURE REDACTED

Figure 2 from Hunter et al. (2010). Polar bear life cycle graph; σi is the probability an individual in stage i survives from time t to t + 1, σL0 and σL1 are the probabilities that at least one member of a cub-of-the-year or yearling litter, respectively, survives from time t to t + 1, f is the expected size of a yearling litter that survives to 2 years, and βi is the conditional probability, given survival, of an individual in stage i breeding and thereby producing a cub-of-the-year litter with at least one member surviving until the following spring.

Lasker HR. 1990. Clonal Propagation and Population Dynamics of a Gorgonian Coral. *Ecology* 71:1578–1589. DOI: 10.2307/1938293.

Hunter CM, Caswell H, Runge MC, Regehr EV, Amstrup SC, Stirling I. 2010. Climate change threatens polar bear populations: a stochastic demographic analysis. *Ecology* 91:2883–2897. DOI: 10.1890/09-1641.1.

Line 127 – There is something I'm missing here. In the intro the authors state that their hypothesis '(H1b) the position of species' populations along the demographic buffering continuum is not predicted by the fast-slow and reproductive strategies continua', but here say 'Our analyses provide support for H1b in plants, but not in animals. Indeed, the position of plant populations along the demographic buffering continuum, as quantified by |∑E_v^σ |, is predicted by the fast-slow and reproductive strategies continua'. I get that the authors then state that this isn't the case when phylogenetic ancestry is considered (and then mostly focus on plants), but the wording here is quite confusing. It took me a few readings of the full paragraph to get what the authors mean here, and so I expect others might get tripped up as well.

R21> We agree with the reviewer that there were a few sentences in the previous version where the hypothesis H1b and its results/conclusions were not sufficiently clear. To improve this point, we have slightly reframed H1b, to make it clear, and it now states:

L. 86-88: "*the position of plant species' populations along the buffering continuum is only weakly predicted by the fast-slow and reproductive strategy continua*"

Moreover, this new statement is followed by a detailed explanation of why life history strategies can only partially predict the position along the buffering continuum. In brief, potential factors the diminish the contribution of life-history strategies to predicting species' positions along the buffering continuum include: different contributions/responses of vital rates to buffering, evolutionary history, and the extent of environmental stochasticity populations face. When all these factors are accounted for, we should expect a gradual reduction in the importance of the fast-slow continuum and reproductive strategies in determining populations' position along the buffering continuum.

Lines 96-104 "*Finally, none of the existing studies exploring demographic buffering have explicitly considered the amount of environmental stochasticity faced by the studied population. This omission likely inflates the perceived roles of life history traits and phylogenetic relationships in explaining population responses to environmental variability, which leads us to hypothesise (H3) that the extent to which a natural population displays demographic buffering strongly depends on its environmental regime. Specifically, higher stochasticity in precipitation and temperature is expected to lead to greater variances in vital rates, shifting populations toward a less buffered end of the demographic buffering continuum*"

Line 173, does the '>> 0' symbol not indicate 'much lower than zero', whereas here the authors mean 'approaching zero'

R22> By mathematical convention, ">>0" means "much greater than zero". However, we thank the reviewer for his comment which helped us spot a mistake that might have caused some confusion. Specifically, in the previous version, we stated that the buffering end was placed on the right side, when in reality it is on the left. This is now fixed and the figure was moved to supplementary material (lines 791-793). The new sentence provides the correct information and avoids further misunderstandings.

Lines 791-793: "*At the buffering-end of the continuum (left; $|\sum E_v^\sigma|$ ~0), vital rates either vary less or the observed variation has little to negligible effect on* λ_s. *At the not buffered end (right;* $|\sum E_v^\sigma|$ *>>0), the observed variation on vital rates strongly shapes* λ_s values."

Line 201 – saying that plants and animals fall along a single axis in their responses to environmental stochasticity seems a bit off since only one axis is investigated. Just a slightly word clarification here would help

R23> We agree with this concern. In rewriting this sentence, we want to emphasise that populations' responses to environmental stochasticity can be captured by life history and evolutionary history along a single axis of variation, as we have shown – from more buffered to less buffered. Please see new lines 179-186.

Lines 184-191: "*Despite significant progress in the pertinent theory*[17] *and praxis*[43], *we still lack a solid understanding of the drivers of population responses to environmental stochasticity, their relationship to the well-known fast-slow and reproductive strategy continua, and the role of evolutionary history and environmental regimes*[17,27]. *Here, we*

provide a critical assessment of this knowledge gap and report several key findings: (i) Populations' responses to environmental stochasticity can be linked to life-history strategies and environmental stochasticity along a single axis of variation, from more buffered to less buffered;[….]"

Line 479 – There is something here that doesn't seem right. Is 'Delta T precip' supposed to be the amplitude in trend of precipitation (not temperature)? As written, it doesn't make sense with the result below of PC2Env

R24> We thank the reviewers for bringing this point up to our attention. It is indeed ΔT_{Prec}. We have corrected this typo in the revised manuscript.

Line 788 should read 'Pearson's correlation'

R25> Done.

Line 794 – PC3 here explains very nearly as much variance in PC2. Is there a formal criteria the authors used to decide on two PCs instead of more? It's not super clear from just the scree plot.

R26> We thank the reviewer for the opportunity to clarify this point. Please, see response R10b for a detailed reply.

In brief, we initially decided to cut off the number of axes to two because it was sufficient to explain 50% of total variation. We realised that this might not be a common practice, and then decided to define a threshold to include all axes with eigenvalues greater than 1, following established statistical approach. This is now reflected in the Methods section. Please, see new lines 473-475.

Lines 477-479: "*Similarly to the PCA of life history traits, we performed the PCA using the PCA function from the factoMineR package in R*[76]*.We retained the first three axes because they show eigenvalues greater than 1 and together, they explained 68.21% of the variation.*"

Line 834- I particularly like the inclusion of the table listing all the studies/species included in the analysis. Good for transparency.

R27> We appreciate the feedback. We have worked hard to improve transparency and reproducibility in this manuscript.

NCOMMS-24-49711-T

Dear editor,

We are grateful for the overall positive evaluation of our previous revision and for the additional feedback provided, as well as for the opportunity to further clarify our work and findings. We agree with the suggestions made by both reviewers, and we have carefully implemented these recommendations in the newly revised manuscript. The main modifications include:

- Change the term “reaction norm” to simply “environmental-vital rate response”, which clarifies its meaning. In the previous version, we used the term “environmental-vital rate reaction norm” to refer to the shape of a given vital rate’s response under a distribution of environmental conditions. We agree, however, that the previous usage of the term “reaction norm” was perhaps not the most suitable term, despite being frequently used in influential articles on demographic buffering. See the response R1 for a detailed clarification of how/where we have changed it.
- We have now: 1) provided a clear definition of environmental stochasticity (R2d); 2) established a clear link between the metrics of environmental stochasticity derived in our study and vital rate stochasticity (R2d); 3) better described the biological interpretation of the axis $PC1_{Env}$ (R2a); 4) included an additional analysis in the Supplementary Material using the raw environmental variables instead of the PCA axes (R3c). Finally, we highlight that most of the discussion and the title of our manuscript were driven by the phylogenetically corrected MCMCglmm model used to determine populations’ position along the buffering continuum, $|\sum E_v^{\sigma}|$. The results of this analysis show that only $PC2_{Env}$ was significant, $not\ PC1_{Env}$. Thus, most of the discussion, including the title, were based on the definition of $PC2_{Env}$, not the definition of $PC1_{Env}$. Given the limited importance of $PC1_{Env}$, any potential concerns regarding the biological interpretation of this axis should be considered of minor importance (R2e).

Below the reviewers' comments are followed by our numbered reply (i.e., responses R1 and R2)

Reviewer #1

Thank you for your thorough and careful revisions. The focus on plants has significantly improved the clarity of your results, and the updated figures are much more effective in conveying your key points. However, I still find the manuscript quite technical in both its framing and language. While your contributions to understanding demographic buffering are valuable, some sections—particularly the discussion of Jensen's inequality and the shape of environment–vital rate reaction norms—remain difficult to follow for a broader audience. This may limit the accessibility of your findings beyond a specialist readership.

I also remain unconvinced by the emphasis on convex (U-shaped) reaction norms as a key conceptual innovation. While this is an interesting mathematical possibility, from a biological standpoint U-shaped functional relationships are not commonly expected. More often, bell-shaped (∩-shaped) curves—such as those seen in thermal performance or tolerance—are anticipated due to physiological limits or trade-offs. The relevance of U-shaped norms for vital rates is not clearly justified, and I would encourage you to either provide stronger biological motivation or shift the emphasis toward more ecologically grounded scenarios. Despite these concerns, I found your work to be rigorous and intellectually engaging.

R1> We thank the reviewer for raising this comment and providing us with the opportunity to tackle it more explicitly. We understand the scepticism of the reviewer with the convex (U-shaped) reaction norms and the difficulty in following some technical aspects of the introduction. To address both points, we first recognise the inaccurate use of the term "environmental-vital rate reaction norm" in the previous version, which we have now replaced with "environmental-vital rate response" in the revised submission, as shown below:

Lines 61-66 **(we highlight the alternative to the term 'reaction norm' in bold)**: *So far, the link between life-history continua and demographic buffering remains largely untapped for multiple reasons. First, we still lack a general understanding of how each vital rate drives populations' responses to environmental stochasticity. Second, the demographic buffering hypothesis*[16,17] *often assumes linear or concave (∩-shaped)* ***environmental-vital rate responses***[19–21]*, which tend to result in negative effects in the long-term of stochastic population growth (λ_s) when environmental variation increases*

We argue that the inaccurate use of the term 'reaction norm' is likely to have caused confusion in our paper, making the text sound overly technical. As such, in addition to having removed the term 'reaction norm', we have carried out the following actions in the lines 66-73 (shown below):

1. We rewrote the sentence describing the U-shaped relationship between environmental conditions and vital rates. This revised version focuses on the exponential response to environmental conditions, which can be considered a form of U-shaped response.

2. We removed the reference to 'Jensen's inequality' in order to make the introduction more accessible to a general audience

3. We included a brief statement acknowledging that U-shaped environmental–vital rate responses are highly context-dependent—for instance, at extreme environmental conditions, performance may decline, resulting in more complex shapes such as sigmoid or U-shaped curves followed by a drop. Therefore, their occurrence in nature and their role as an alternative to demographic buffering (which typically assumes concave responses) remain debated.

Lines 66-73: *Alternatively, some vital rates, particularly growth or reproduction, may be convex (U-shape; e.g., exponential response)* [20,22]*, showing disproportionately high values under favourable conditions,*

which then offset losses in bad years[23]*. In these alternative cases, the resulting stochastic population growth rate,* λ_S*, may exceed the deterministic population growth rate,* λ*, obtained from the mean environment. This outcome, while theoretically possible, requires (1) a substantial contribution from the vital rate to* λ *and (2) environmental variability that stays within a range where its response remains convex. The likelihood of both conditions occurring in nature is debatable*[24]*.*

All the changes mentioned above have been incorporated into a single paragraph. Below, we present these revised lines within their corresponding paragraph. An in-depth discussion is provided in the following section in case the reviewers require further evidence.

Lines 61-79: *So far, the link between life history continua and demographic buffering remains largely untapped for multiple reasons. First, we still lack a general understanding of how each vital rate drives populations' response to environmental stochasticity. Second, the demographic buffering hypothesis*[16,17] *often assumes linear or concave (∩-shaped)* ***environmental-vital rate responses***[19–21]*, which indeed tends to result in negative effects on long-term of stochastic population growth (*λ_S*) when environmental variation increases. Alternatively, some vital rates, particularly growth or reproduction, may be convex (∪-shape; e.g., exponential response)*[20,22]*, showing disproportionately high values under favourable conditions, which then offset losses in bad years*[23]*. In these alternative cases, the resulting stochastic population growth rate,* λ_S*, may exceed the deterministic population growth rate,* λ*, obtained from the mean environment. This outcome, while theoretically possible, requires (1) a substantial contribution from the vital rate to* λ *and (2) environmental variability that stays within a range where its response remains convex. The likelihood of both conditions occurring in nature is debatable*[24]*. Third, and equally important, environmental stochasticity has, to date, not been explicitly considered in studies of demographic buffering. Consequently, populations exposed to reduced temporal variability in climate variables during the study time frame are prone*

to being interpreted as buffered, regardless of whether they have been exposed to high or low environmental stochasticity. Altogether, these complementary parts of the demographic buffering hypothesis remain a major challenge in linking life history continua to populations' response to environmental stochasticity in the context of climate change.

In this further discussion, we refer to Koons et al. (2009), a notable piece to the development of demographic buffering theory, to showcase the use of the term reaction norm and to show how U-shaped environmental–vital rate responses may manifest. While we have already addressed the reviewer's concern regarding our use of the term *reaction norm*, we include this example to clarify any potential concerns that may remain about our rationale for the shape of environmental–vital rate responses.

Below, we reproduce the Figure 1 from Koons et al. (2009), which shows four hypothetical environmental-vital rate responses to environmental conditions. The green and yellow lines are the most important for our case and represent two distinct responses that nevertheless show essentially the same sigmoidal shape. Although these lines are better described as sigmoidal, both include an exponential-like phase characterised by a positive second derivative, which we indicate with red arrows. While the red arrows mark the points of maximum curvature (i.e., strongest convexity), the red dots indicate the approximate range over which the environmental–vital rate responses can be interpreted as U-shaped. Notably, although most of the green curve remains convex throughout its trajectory, its overall shape can still be generalised as U-shaped within the relevant environmental range.

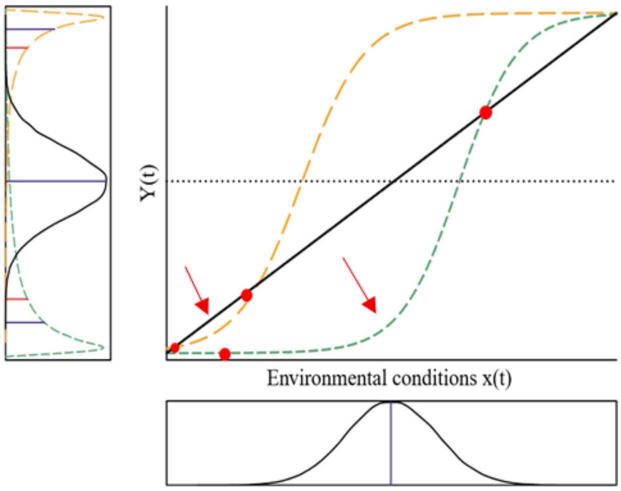

Above, we display a modified Figure 1 from Koons et al. (2009). This figure shows four different responses of vital rate (Y(t)) to environmental conditions, which are described as reaction norms; see the underlined sentence in the original legend. **Original legend***: Four hypothetical reaction norms between environmental conditions (x) and any demographic parameter Y. The lower box illustrates mean environmental conditions (blue bar) and its temporal distribution. The left box indicates resulting temporal distributions of Y(t) for each curve (i.e. reaction norm) in the main figure (blue bars indicate Y(t) in average environmental conditions, and red bars indicate the temporal mean of Y(t) across all environmental conditions Y): Depending on the reaction norm, environmental stochasticity may decrease (orange dashed lines), have no effect (black solid lines), or increase (green dashed lines) Y relative to its value in average environmental conditions. The black dotted line serves a reference where Y(t) is perfectly robust to environmental change.* ***MODIFICATIONS: We included two red arrows showing where values of the second-order derivative are positive, which characterise U-shape portions of the reaction norm informed by Koons et al. 2009.***

Despite not being shown in the figure provided, another kind of U-shape response is the exponential response. However, we agree that the prevalence of the U-shape response is highly dependent on the extent of the environmental conditions measured. For

instance, even if individual growth responds exponentially to temperature (let's say an insect), this response will decay if the temperature exceeds the physiological limits of the species.

Finally, we recognise that completely U-shape curves might not be as common in nature, particularly in an adaptive way, as described by Morris and Doak (in press). We have recognised this limitation to be more realistic and in line with Morris and Doak (in press). It has been addressed in lines 70-73, included above and pasted below again for convenience:

Lines 70-73: *This outcome, while theoretically possible, requires (1) a substantial contribution from the vital rate to λ and (2) environmental variability that stays within a range where its response remains convex. The likelihood of both conditions occurring in nature is debatable*[24]

References

1. Koons DN, Pavard S, Baudisch A, Jessica E. Metcalf C. 2009. Is life-history buffering or lability adaptive in stochastic environments? Oikos 118:972–980. DOI: 10.1111/j.1600-0706.2009.16399.x.
2. Morris WF, Doak DF. What is demographic lability and when might we expect to see it? The American Naturalist. DOI: 10.1086/737720.

Reviewer #2

This is Reviewer 2 from the first round of revisions. First off, I really appreciate all of the effort that the authors clearly put into the revision. I think that the focus on the plant data makes the manuscript much stronger. The clarification to the writing and figures has also made the manuscript less dense and much easier to read. Adding analyses, such as examining time series length, incorporating PC3 etc., have made conclusions more

robust than in the previous version. I can tell this was a lot of work, and think the manuscript is much improved.

R2> We appreciate the positive evaluation of our revision, and once again, we are thankful for the opportunity to clarify some remaining areas for improvement. Due to our somewhat long response below, we have broken it down into different pieces to reply point-by-point. Each point is then assigned a letter: R2a, R2b, R2c, etc.

In summary:

- **R2a:** We recognised the combined contribution of $R^{CV}_{TempMax}$ and $\underline{T}_{TempMax}$ in determining $PC1_{Env}$, and we have made several adjustments to better clarify the biological interpretation of this axis. In brief, we have retained the interpretation of $PC1_{Env}$ as representing 'stochasticity in temperature'; however, we now also highlight a general trend in which $PC1_{Env}$ decreases towards the tropics. We argue that this latitudinal trend, which is shaped by both $R^{CV}_{TempMax}$ and $\underline{T}_{TempMax}$, provides the most relevant context for interpreting the ecological meaning of the $PC1_{Env}$.
- **R2b**: We demonstrate that, despite $R^{CV}_{TempMax}$ and $\underline{T}_{TempMax}$ showing high R^2 values in relation to $PC1_{Env}$, the contribution of $R^{CV}_{TempMax}$ to $PC1_{Env}$ is stronger than $\underline{T}_{TempMax}$. Consequently, $R^{CV}_{TempMax}$ and $\underline{T}_{TempMax}$ are not interchangeable to define $PC1_{Env}$, as the reviewer had suggested.
- **R2c**: We performed the MCMCglmm analysis using the raw variables originally used to build both the environmental and life-history PCAs. Despite yielding interesting results — including support for $R^{CV}_{TempMax}$ being more important than $\underline{T}_{TempMax}$ — we discuss that this analysis may not be appropriate due to several limitations.
- **R2d:** We defined what we mean by stochasticity and how it is related to the demographic and environmental metrics used in our study.

We think that there are two key points that must be clarified in advance to ensure our response is concise and coherent: (i) the definition of environmental stochasticity and

(ii) the role of temperature stochasticity in our MCMCglmm models. First, we define stochasticity as environmental variability. A detailed explanation of how this was done, along with its rationale, is provided in the relevant section (see R2d). If the reviewer thinks that a deeper understanding of our definition of stochasticity is necessary to proceed, we suggest starting with the R2d response. Second, it is important to note that most of our discussion is based on the phylogenetically corrected model, in which $PC1_{Env}$ — the axis most closely aligned with both $R^{CV}_{TempMax}$ and $\underline{T}_{TempMax}$ — was not significant. Thus, we consider that the validity of H3 depends minimally on the definition of $PC1_{Env}$, which was not significant to define species position along the buffering continuum.

I still have one major concern remaining for the revision, which is that the link between demographic results and environmental stochasticity as a driver is not, in my view, robust. This relates to previous comment 2b, response R10b>, and the interpretation of PC1 as environmental stochasticity.

To clarify, I have no concerns about the validity of the statistical tests, but rather their interpretation, which I do not feel was addressed in the last round of revision—I think this was just a miscommunication between myself and the authors on the comment. To hopefully clarify for this round, the authors arguments for H3 relies on the role of PC1, which they argue is equivalent to environmental stochasticity. Yet, in PCA analyses, all six environmental variables do (to varying levels) contribute to different PC axes. Indeed, from Figure S4 (right hand side), it looks like bar(T)_TempMax also contributes very significantly to PC1, with other environmental variables, such as bar(T)_Prec contributing, but with a more moderate correlation coefficient. Thus, from the PCA analyses, it is not robust to say that relationships with PC1 are driven by environmental stochasticity. Rather, these results could (nearly) equally be driven by negative relationships with bar(T)_TempMax, given the strong correlation with PC1.

R2a> We agree that, although $PC1_{Env}$ appears to be highly defined by $R^{CV}_{TempMax}$, $\underline{T}_{TempMax}$ still makes a substantial contribution to defining its axis, and this cannot be

ignored. While we have always been cautious, we agree that this caution was not reflected in the text. We have made a few amendments to the Methods and Discussion sections to ensure that the text is more transparent. In brief, we now discuss how $PC1_{Env}$ relates to latitude and how it must reflect the difference between the tropical and temperate realms. See lines 481-495 (Materials and Methods) and 268-285 (Discussion). Please note that Figure S5 in the previous version of the manuscript already provided the necessary visual representation of $PC1_{Env}$. However, in that version, both $PC1_{Env}$ and $PC2_{Env}$ were rescaled in the figure to make size and colour directly comparable. We have now reconsidered this approach and restored both variables to their original scales. These changes are likely to increase the transparency of the relationship between $R^{CV}_{TempMax}$ and $\underline{T}_{TempMax}$, and how it relates to demographic buffering in the studied populations

> Lines: 481-495 **(in bold, we highlight the general trend between $R^{CV}_{TempMax}$ and $\underline{T}_{TempMax}$ and the potential biological meaning of this correlation)**: "Similar to the PCA of life-history traits, we performed the PCA for the environmental variables using the *PCA* function from the *factoMineR* package in R[76]. We retained the first three axes because they show eigenvalues greater than 1, and together they explained 68.21% of the variation. Principal component axis 1 ($PC1_{Env}$) explains 30.08% of the variation, and primarily reflects a gradient contrasting high mean maximum temperatures with high stochasticity in maximum temperature. **Accordingly, populations with lower $PC1_{Env}$ scores are characterised by warmer but more stable temperature (lower values of $R^{CV}_{MaxTemp}$), whereas those with higher $PC1_{Env}$ scores experience cooler but more variable thermal environments (Figure S4). A closer examination of $PC1_{Env}$ reveals that it decreases towards the tropics, mirroring the expected trend of higher $\bar{T}_{MaxTemp}$ and lower $R^{CV}_{MaxTemp}$ in tropical regions (Figure S5).** In contrast, $PC2_{Env}$, which explains 20.59% of the environmental variation, describes differences in the amplitude of trend in precipitation, ΔT_{Prec}. $PC2_{Env}$ is higher in more arid places such as deserts and the Mediterranean region. Therefore, we refer to these environmental axes as temperature stochasticity

($PC1_{Env}$) and seasonal aridity ($PC2_{Env}$). Finally, the third axis of the environmental PCA, $PC3_{Env}$, explained 17.55% of the environmental variation and was related to the amplitude of maximum temperature in the time series ($\Delta T_{TempMax}$), which is likely to reflect how the environment has changed during the study time."

Lines 268-285 (**in bold, sentence highlighting a general trend of $PC1_{Env}$ in the discussion section**): *"A second area for improvement is the suboptimal representation of long-term demographic studies across taxonomies and geographies required for comparative demographic studies. The existing databases we use here, though state-of-the-art in regards of number of demographic studies, still remain biased toward perennial species in the Plant Kingdom[52,53]. For instance, while the robustness of survival as the main driver of buffering holds for most of our studied populations, annual plants may represent an alternative strategy for how survival-reproduction trade-offs shape population performance in stochastic environments. However, the limited sample size for annual plants limited any inference pointing out the need for further studies. Similarly, given the small amount of available data on animal populations, we failed to perform similar analyses satisfactorily for comparison. With this taxonomic coverage, filling the demographic gap across continental scales, particularly in South America and Africa, remains a pressing issue to gain a holistic understanding of how natural populations respond to environmental stochasticity[52,53].* ***It is worth noting that stochasticity in temperature shows a trend to increase from tropical areas to higher latitudes, suggesting further studies comparing demographic buffering across latitude might be an interesting venue.*** *However, the acquisition and communication of high-resolution demographic data take, by definition, a long time, which makes filling this gap an enduring commitment by population ecologists in the coming decades[50].* "

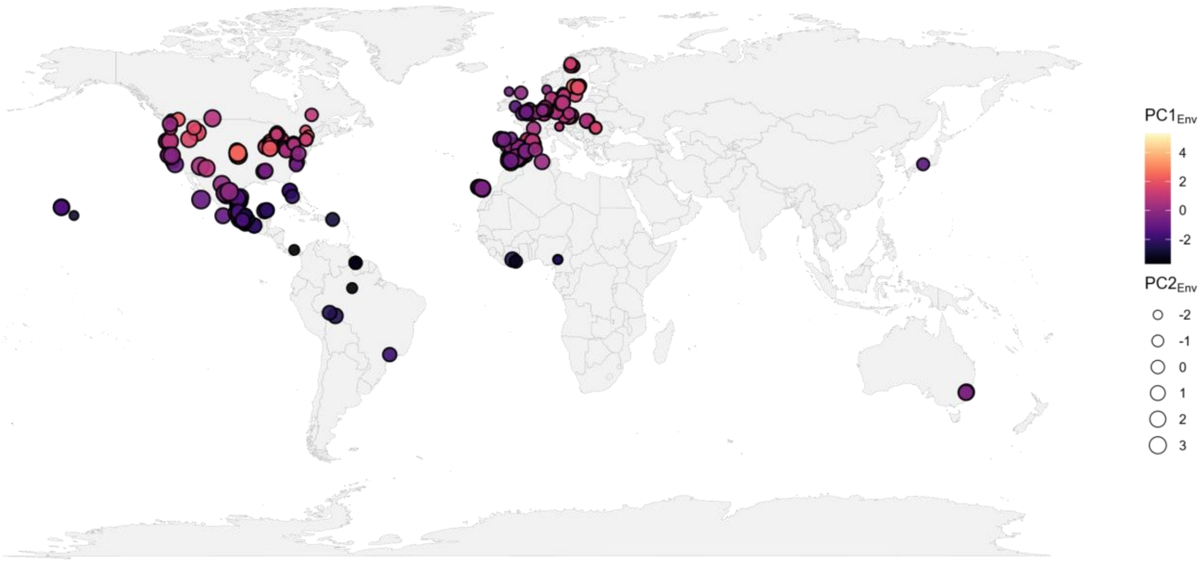

Figure S5. The 121 studied populations from 78 plant species are mapped along the environmental gradients captured by the environmental principal components analysis. $PC1_{Env}$, which represents stochasticity in temperature fluctuations, shows a noticeable trend of increasing with latitude. Despite a more elusive trend, it is possible to identify that $PC2_{Env}$ increases from continental to insular areas; however, this pattern might be masked by topographic features (e.g., altitude), which are not shown.

As such, an equivalent interpretation and/or framing for the manuscript, given the statistical tests, could center on the role of the mean value of the maximum temperature in driving demographic buffering. In other words, the authors could use these exact analyses (or nearly so) to write a paper about the role of the mean trend of maximum temperature in driving demographic buffering, including a brief caveat sentence that this environmental variable had a strong positive relationship with mean minimum temperature and a strong negative correlation with relative stochasticity of maximum temperature (from Figure S3). At the end of the day, the challenge lies in the strong correlations between environmental drivers, which is a challenge across a lot of ecology! These strong correlations mean that multiple variables map onto the same PCA axis, it appears. Further, given these strong correlations arise in the datasets analyzed here, interpreting this as the effect of environmental stochasticity alone is a concern to me.

R2b> While we agree that $\underline{T}_{TempMax}$ contributes nearly as much as $R^{CV}_{TempMax}$, the loadings of $PC1_{Env}$ favour $R^{CV}_{MaxTemp}$ (R^2 = 0.87) over $\underline{T}_{TempMax}$ (R^2 = -0.74). Therefore, these two variables do not contribute equally to defining $PC1_{Env}$, and they cannot be used interchangeably as the reviewer suggested (please see the table of R^2 values for the environmental PCA axes below, it has been provided in Supplementary Material S2).

Also, please note that this difference is particularly relevant for $PC2_{Env}$, where the contrast is even more pronounced: seasonality in aridity plays a much larger role in determining $PC2_{Env}$ than the mean value of the precipitation trend (ΔT_{Prec} = 0.79 versus $\underline{T}_{Prec}$ =–0.548).

Table showing R^2 of the PCA's axes and the raw variables

```
## $Dim.1
##
## Link between the variable and the continuous variables (R-square)
##
=============================================================================
## correlation p.value
## R^CV_MaxTemp           0.8668087  6.183297e-100
## Ampli_trend_TMin       0.4590744  2.130222e-18
## Ampli_trend_Prec       0.2177142  7.386559e-05
## Ampli_trend_TMax       0.1981180  3.193055e-04
## Mean_trend_Prec       -0.4508056  1.008298e-17
## T_TempMax             -0.7434070  1.507656e-58
##
## $Dim.2
##
## Link between the variable and the continuous variables (R-square)
```

```
##
===========================================================================
===========
##                    correlation      p.value
## Ampli_trend_Prec    0.7886580 2.021982e-70
## Mean_trend_TMax     0.4022397 4.147529e-14
## Ampli_trend_TMin   -0.2481373 5.784008e-06
## Ampli_trend_TMax   -0.2935288 6.707701e-08
## Mean_trend_Prec    -0.5476851 6.527546e-27
```

I note that in the authors' response, they state that they couldn't remove the mean trend of temp, and I agree. The problem isn't that it is included, but rather that it ALSO contributes a decent amount to PC1, and as such interpretation of results of PC1 cannot be as simple as environmental stochasticity, since other environmental variables – that do not relate to stochasticity whatsoever – also contribute to PC1.

Instead, I wonder about actually examining the environmental stochasticity effects directly themselves, rather than through a PCA analysis, with the different PC axes used as covariates. Doing so would allow for incorporating other environmental covariates that the authors think/know are important, while directly interpreting stochastic effects themselves. Even with this, including a discussion about the challenges in disentangling environmental stochasticity effects is critically, especially given the strong correlations in environmental covariates.

R2c> We appreciate the suggestion to perform the GLMM analysis using the original environmental variables rather than the PCA axes. Accordingly, we conducted this analysis and have included the results in Supplementary Material S1 (see lines 907-919). This may help readers check how such variables determine buffering. Although this analysis is valuable to provide insights for further studies, it suffers from several drawbacks, such as the following:

1. The model included a large number of environmental variables (six in total), all of which are related in some way to mean and variance values in the time series. This increases the risk of false positive relationships (Type I error). It is also important to note that the same model structure was applied separately to each of the five vital rates (survival, reproduction, growth, shrinkage, and clonality), likely compounding the risk of Type I error.
2. Interactions among environmental variables may have biological relevance and were indeed shown to be important in our current model selection, particularly in the interactions $PC1_{Env} \times PC2_{Env}$ and $PC1_{LH} \times PC2_{LH}$. However, including such interactions would exponentially increase the number of parameters in the model, potentially leading to overfitting. Therefore, we chose not to include interaction terms in this analysis.
3. Since we used raw variables from the environmental PCA in this analysis, we considered it appropriate to apply the same rationale to the life-history PCA and use the raw life-history variables as well. As mentioned earlier, interactions between $PC1_{LH}$ and $PC2_{LH}$ likely occur in nature, but were not included for the same reason - to avoid overparameterizing the model. The same concern applies to potential interactions between environmental and life-history PCA variables (i.e., $PC1_{LH}*PC2_{LH}*PC1_{Env}*PC2_{Env}$), which were also excluded for this reason.

Given the issues discussed above, we argue that it is more appropriate to present this analysis in Supplementary Material S1, allowing readers to interpret the results and draw insights independently, rather than including it in the main manuscript. In this context, it is particularly noteworthy that $R^{CV}_{MaxTemp}$ and ΔT_{Prec} were the only environmental variables found to be significant in the model (see Figure S6, new lines 949-961). This result supports both our discussion and our methodological approach, particularly our interpretation of $PC1_{Env}$ as primarily reflecting the influence of $R^{CV}_{MaxTemp}$."

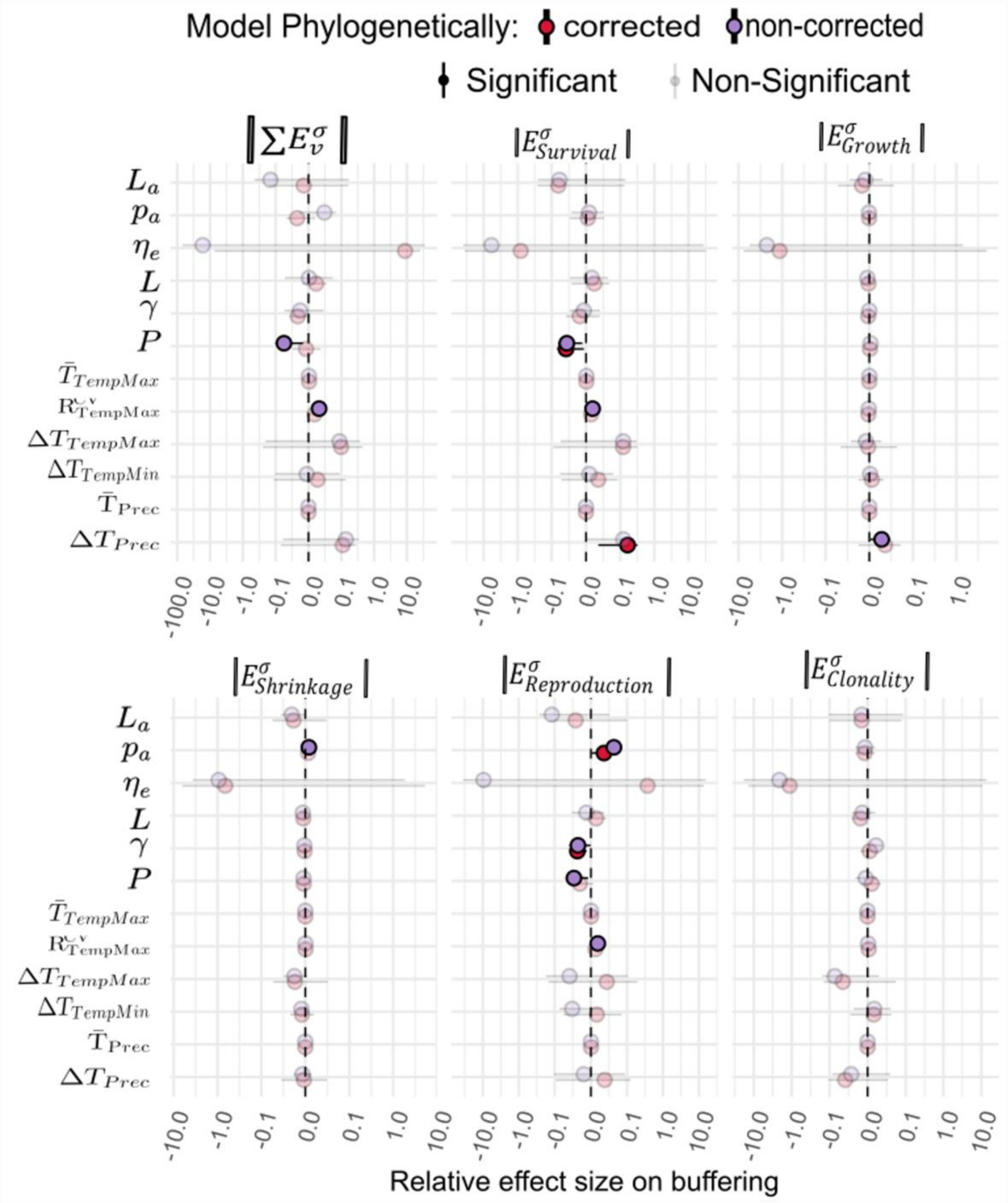

Figure S6. Effects of life-history traits and environmental variables on the positions of the 121 plant populations studied along the buffering continuum and on the contributing vital rates. MCMCglmm models with (red) and without (purple) phylogenetic correction are shown. Models are additive and do not include interactions. Notations represent six life-history traits and six environmental variables as follows. Life-history traits: individual development (γ); mean life expectancy (η_e), distribution of mortality risk throughout the life cycle (P); probability of achieving reproduction before

dying (p_α); mean age at first reproduction (L_a); and reproductive window (L). Environmental variables: mean trend in maximum temperature ($\underline{T}_{TempMax}$); relative stochasticity of maximum temperature ($R^{CV}_{TempMax}$); relative amplitude of the trend of the maximum temperature ($\Delta T_{TempMax}$); relative amplitude of the trend of the minimum temperature ($\Delta T_{TempMin}$); mean value of the precipitation trend ($\underline{T}_{Prec}$); and relative amplitude of the trend of the precipitation (ΔT_{Prec}).

All of this led to an important, but more minor comment, which is that the manuscript never defines environmental stochasticity. I think this is really important to do in the first or second paragraph, especially given confusion in the literature about stochasticity vs. variability vs. heterogeneity vs. unaccounted for “randomness.”

R2d> We thank the reviewer for pointing out the complexity of the term environmental stochasticity and the necessity to define it clearly in the manuscript. While environmental stochasticity has been described many times in the context of demography as *variation in vital rates across the time* (e.g., Lande et al. 2003, Tuljapurkar et al. 2003,), we realised that this definition does not necessarily hold across other fields in ecology (see Shoemaker et al. 2020). Because our goal in this paper is not to deepen the discussion about the definition of environmental stochasticity, but rather to understand the role of vital-rate responses to environmental variability, we have decided to define environmental stochasticity as temporal variability toward the mean value of the chosen climatic variables (i.e., temperature and precipitation) (see lines 40-42).

> Lines 40-42 “*Climate change projections anticipate a global increase not only in the mean of key abiotic drivers (e.g., temperature, precipitation), but also their temporal variability*[1] *– the so-called environmental stochasticity.*”

This simplified description is intended to avoid overcomplicating the Introduction section of our manuscript, which Reviewer #1 already pointed out as too technical (see R1 above). Later (lines 450-469), during the description of the choice of environmental

drivers of vital rate variation, we define demographic buffering and how this phenomenon is related to the environmental stochasticity, as well as how the metrics derived from the environmental time series match the concept of demographic stochasticity used in our study.

> Lines 450-469 **(in bold, we link the concept of demographic stochasticity with the biological meaning of the environmental variables in our study):** *Because we are interested in demographic responses to environmental stochasticity rather than mean temperature or precipitation, we decomposed each climatic variable Y into three groups of variables that represent trend (T), seasonality (S), and stochasticity (R). We derived these variables using a seasonal decomposition through an additive moving averages model toward the decompose function in the base R package[75]. Briefly, this function computes the trend component of the variable of interest (e.g., monthly temperature) using an additive moving average approach. This trend is then subtracted from the time series. The resulting time series represents the periodic fluctuation - the seasonality component (S). Finally, trend and season are discounted from the raw time series and the residuals represent the stochasticity of the environmental component - R[75]. For each component of time series Y (i.e., T_Y, S_Y, R_Y), this approach allows us to derive the mean ($T_Y^{-}, S_Y^{-}, R_Y^{-}$), relative amplitude ($\frac{max-min}{mean}$; $\Delta T_Y, \Delta S_Y, \Delta R_Y$), and coefficient of variation (relative stochasticity hereafter, $T_Y^{CV}, S_Y^{CV}, R_Y^{CV}$).* ***It is worth recalling that the studied variable (demographic buffering, $\sum E_v^{\sigma}$) captures the extent to which temporal variation in vital rates affected the population growth rate. Therefore, we only retained those climatic variables strictly related to changes in mean-variance environment and with direct biological or climatic meaning, as they are the potential sources of variation for these vital rates.*** *Thus, only the mean trend of the environmental conditions (T_Y^{-}), amplitude of trend component (ΔT_Y) and the relative stochasticity of the random component (R_Y^{CV}) were retained. While the R_Y^{CV} component represents the monthly environmental uncertainty, the other environmental variables*

represent how the environment has changed during the study time compared to the mean conditions."

We argue that this approach provides the necessary definitions and background for non-expert readers to understand what we mean by environmental stochasticity and how it matches the variability in the vital rates we have estimated in our work.

References:

- Lande, R., Engen, S., & Saether, B. E. (2003). *Stochastic population dynamics in ecology and conservation*. OUP Oxford.
- Tuljapurkar S, Horvitz CC, Pascarella JB. 2003. The Many Growth Rates and Elasticities of Populations in Random Environments. *The American Naturalist* 162:489–502. DOI: 10.1086/378648.
- Shoemaker LG, Sullivan LL, Donohue I, Cabral JS, Williams RJ, Mayfield MM, Chase JM, Chu C, Harpole WS, Huth A, HilleRisLambers J, James ARM, Kraft NJB, May F, Muthukrishnan R, Satterlee S, Taubert F, Wang X, Wiegand T, Yang Q, Abbott KC. 2020. Integrating the underlying structure of stochasticity into community ecology. *Ecology* 101:e02922. DOI: 10.1002/ecy.2922.

Finally, I note that neither H1 or H2 rely on interpreting environmental stochasticity, which means that many of the results feel really strong to me, despite my longwinded comment above. It's H3 that is problematic, and, to me, reads as the key framing for the manuscript. Yet, H1 and H2 are still really interesting, especially with the revised wording for H2 (which helped me a lot).

R2e> We appreciate the recognition that H1 and H2 are robust and independent of the definition of environmental stochasticity and the MCMCglmm models. Moreover, we hope to have demonstrated that the reviewer's concerns about the $PC1_{Env}$ have been properly addressed and should now be considered solved or minimised, given that:

1) $PC1_{Env}$ is now much better described in the manuscript; and

2)The $PC1_{Env}$ axis plays a limited role in our conclusions, as it was not significant in the central MCMCglmm analysis used to determine populations' positions along the buffering–lability continuum.

Reviewer #3

I am satisfied that the authors have adequately dealt with my previous comments/concerns, and agree that this represents a substantially clearer representation of their findings. My only comment is that I would advise the authors to perhaps re-order the legend in Figure 2 to match the order in which each group appears (top to bottom) in the figure itself. As such, it took me a bit of time to realize that the different life forms were represented by different colors, even though this was indicated in the legend.

R3> Thanks for this acknowledgement as well as for this final advice. We have reordered the legend in Figure 2, and we agree that it was a really good point. Please see the new figure below.

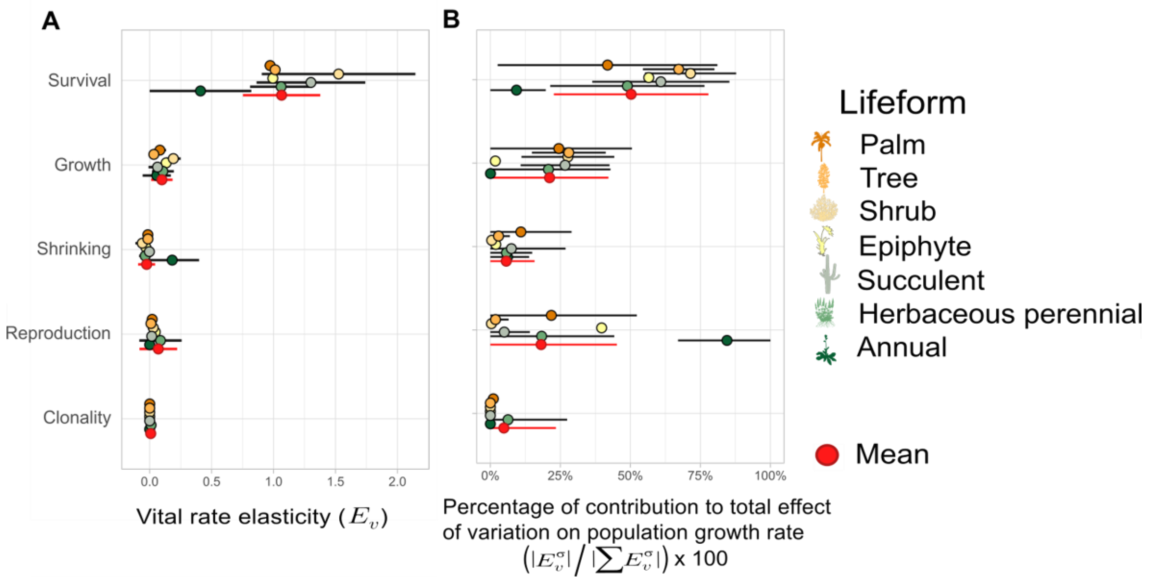

Figure 2. Temporal variance in survival is the primary driver of population responses to environmental stochasticity in most of our 121 plant populations, followed by individual-

level growth and reproduction. (**A**) The proportional contribution of each vital rate (survival, growth, shrinkage, reproduction, clonality) to stochastic population growth rate λ_s quantified from the stochastic elasticities (E_v). **(B)** The proportional contribution of each vital rate to the populations' positions along the buffering continuum, as quantified by E_v^σ. Note that panel B is scaled to show proportional contributions as percentages, calculated as $\left[\frac{|E_v^\sigma|}{\sum|E_v^\sigma|}\right] \times 100$. Different life forms of plants are represented by different colours.

NCOMMS-24-49711-T

We sincerely appreciate the reviewer's patience and continued engagement throughout the review process. Below, we provide a point-by-point response to the reviewer's concern regarding the interpretation and use of the term *environmental stochasticity* throughout the manuscript. These points are addressed below as responses R1, R2, R3, *etc*.

In brief, we recognise that, in previous versions, we did not manage to successfully capture the nuances in the term "environmental stochasticity". While we understand the use of the term "stochasticity" has been used rather permissively in the literature (Vellend et al., 2014; Shoemaker et al., 2020), we have opted to follow the comprehensive and rather effective definition of stochasticity provided by the Reviewer (see **R4**, below). Consequently, we have reframed our manuscript around "environmental variability" (see **R1**), a term that encompasses both deterministic environmental fluctuation and stochastic events (Beissinger & Gibbs, 1993; Fujiwara, 2009). This slight reframing based on the term better aligns with the quantification of the extent to which variability in vital rates affects population growth rate ($\sum E_v^{\sigma}$). This change is also aligned with previous approaches using the *coefficient of variation* of vital rates as a metric to quantify demographic responses in a stochastic environment (Morris & Doak, 2004; Doak et al., 2005; Koons et al., 2009). Indeed, despite the theory regarding demographic stochasticity, key literature cited in our manuscript uses the term environmental variability (or variable environment), such as Stearns (1976), Tuljapurkar et al. (2003) and Haridas & Tuljapurkar (2005). Therefore, replacing 'stochasticity' with 'variability' brings the manuscript into line with the terminology used by its predecessors and more broadly with the common usage in the field.

Finally, we really appreciate the reviewer's persistence in clarifying that our $PC1_{Env}$ is strongly linked to the M*ean value of the maximum temperature trend* ($\bar{T}_{TempMax}$) and *Relative stochasticity of maximum temperature* ($R^{CV}_{TempMax}$). As such, in the revised version, we no longer refer to $PC1_{Env}$ as environmental stochasticity, but as a coupled gradient of mean and stochastic thermal conditions (see **R2** for details). Moreover, we have emphasised that $PC2_{Env}$ — being strongly aligned with the amplitude of the precipitation trend (ΔT_{Prec}) and only weakly related to $R^{CV}_{TempMax}$ — captures the

deterministic component of environmental variability, rather than environmental stochasticity.

References

1. Beissinger SR, Gibbs JP. 1993. Are Variable Environments Stochastic? A Review of Methods to Quantify Environmental Predictability. In: Yoshimura J, Clark CW eds. Adaptation in Stochastic Environments. Lecture Notes in Biomathematics. Berlin, Heidelberg: Springer Berlin Heidelberg, 132–146. DOI: 10.1007/978-3-642-51483-8_7.
2. Fujiwara M. 2009. Environmental Stochasticity. In: Encyclopedia of Life Sciences. Wiley,. DOI: 10.1002/9780470015902.a0021220.
3. Doak DF, et al. 2005. Correctly estimating how environmental stochasticity influences fitness and population growth. The American Naturalist 166:14–21.
4. Haridas CV, Tuljapurkar S. 2005. Elasticities in variable environments: Properties and implications. The American Naturalist 166:481–495. DOI: 10.1086/444444.
5. Koons DN, et al. 2009. Is life-history buffering or lability adaptive in stochastic environments? Oikos 118:972–980. DOI: 10.1111/j.1600-0706.2009.16399.x.
6. Morris WF, Doak DF. 2004. Buffering of life histories against environmental stochasticity: Accounting for a spurious correlation between the variabilities of vital rates and their contributions to fitness. The American Naturalist 163:579–590. DOI: 10.1086/382550.
7. Shoemaker LG, et al. 2020. Integrating the underlying structure of stochasticity into community ecology. Ecology 101:e02922. DOI: 10.1002/ecy.2922.
8. Stearns SC. 1976. Life-history tactics: A review of the ideas. The Quarterly Review of Biology 51:3–47. DOI: 10.1086/409052.
9. Tuljapurkar S, et al. 2003. The many growth rates and elasticities of populations in random environments. The American Naturalist 162:489–502. DOI: 10.1086/378648.
10. Vellend M, et al. 2014. Assessing the relative importance of neutral stochasticity in ecological communities. Oikos 123:1420–1430. DOI: 10.1111/oik.01493.

Reviewer #2 (Remarks to the Author):

After a careful read through of the revised version of "Plant population responses to environmental stochasticity are primarily driven by survival-reproduction trade-offs and mediated by aridity" my major concerns from the last revision remain unaddressed. R1> We understand the reviewer's frustration, and we apologise for delaying addressing this important point. While we understand the use of the term stochasticity is permissively used in the scientific literature, the whole field of demography of stochastic environments has its operational tools based on variability, not stochasticity *per se*. For instance, the manuscript "The Many Growth Rates and Elasticities of Populations in Random Environments" (Tuljapurkar, Horvitz & Pascarella, 2003), where we based the metric to quantify population response to environmental conditions, uses variability (*i.e.*, standard deviation of the vital rate) instead of a strict stochastic component.

The nuance brought up by the reviewer had escaped us in the previous round of review, and we are glad the reviewer insisted on framing our manuscript in a more comprehensive and accurate definition of stochasticity.

For instance, consider the lines 64-66, 83-85, and the title, to see how the manuscript has been reframed around variability.

> Lines 64-66: *So far, the link between life history continua and demographic buffering remains largely untapped for multiple reasons. First, we still lack a general understanding of how each vital rate drives populations' response to environmental* ***variability****.*

> Lines 83-85: *Here, we examine how environmental regimes, key life history traits, and evolutionary history predict the extent to which natural populations of plants can buffer against environmental* ***variability*** *along a demographic buffering continuum.*

> **Title:** *Plant population responses to environmental* ***variability*** *are primarily driven by survival-reproduction trade-offs and mediated by aridity*

The authors still argue that PC1_Env represents environmental stochasticity, but the table they show in the response letter, and their supplementary figures S3, S4, and S5, show that PC1 represents multiple components of the environment. It represents a latitudinal gradient, which in turn represents changes in mean temperature (here bar(T_TempMax)) and variability in temperature (here stochasticity or R CV TempMax) in temperature. In the response they argue that this axis represents primarily environmental stochasticity but it has a correlation of 0.87 with stochasticity while T_TempMax has an almost equally strong negative correlation of -0.74. This is unsurprising and a classic challenge for inferring underlying environmental drivers across a latitudinal gradient, given it is well known that these climate covariates strongly covary, as occurs in this study as well.

The issue remains that the main text introduction, results, and discussion still fully attribute this effect to environmental stochasticity, with only a single sentence at the end (lines 308-310) mentioning this critical fact in the main text. While the methods text is now more precise, this nuance is not translated into the manuscript framing or results. The issue remains that the authors attribute effects to environmental stochasticity when the correlation structure of the covariates makes it difficult or impossible to disentangle temperature stochasticity effects from mean temperature effects from latitudinal effects.

R2> We agree with the reviewer. We have now solved the concern regard the naming of the environmental PCAs via two key changes: (i) we have reframed the manuscript around environmental variability, instead of stochasticity (See R1 above), and (ii) we now stress along the manuscript that $PC1_{Env}$ is better aligned toward a coupled gradient of mean and stochastic thermal conditions, while $PC2_{Env}$ is better aligned with the deterministic component of environmental variability in aridity (see lines 485-493). We also stressed the differentiation between the deterministic and stochastic components of our measurement of environmental variability in lines 461-468. These changes follow the reviewer's suggestions.

Lines 485-493 (**highlighted in bold the names of each environmental principal component axis**): *Accordingly, populations with lower* $PC1_{Env}$ *scores are characterised by warmer but more stable*

temperatures (lower values of $R^{CV}_{TempMax}$), whereas those with higher $PC1_{Env}$ scores experience cooler but more variable thermal environments (Figure S4). A closer examination of $PC1_{Env}$ reveals that it decreases towards the tropics, mirroring the expected trend of higher $\overline{T}_{TempMax}$ and lower $R^{CV}_{TempMax}$ in tropical regions (Figure S5). In contrast, $PC2_{Env}$, which explains 20.59% of the environmental variation, describes differences in the amplitude of the trend in precipitation, ΔT_{Prec}. $PC2_{Env}$ is higher in more arid places such as deserts and the Mediterranean region. ***Therefore, we refer to these environmental axes as coupled gradient of mean and stochastic thermal conditions ($PC1_{Env}$) and aridity amplitude ($PC2_{Env}$).***

Lines 461-469 (**highlighted in bold the differentiation between stochastic and deterministic components of environmental variability)**: *[…] we retained only those climatic variables strictly related to changes in mean-variance environment and with direct biological or climate meaning, as these are the potential sources of variation in the vital rates. Thus, retained only the mean trend of the environmental conditions ($\overline{T}_Y$), the amplitude of the trend component (ΔT_Y), and the relative magnitude of the stochastic component within the environmental variability (R^{CV}_Y).* ***While R^{CV}_Y represents the monthly environmental uncertainty (i.e., the stochastic component of environmental variability), the other two variables (T_Y and ΔT_Y) collectively capture both the mean magnitude of the trend component (T_Y) and the magnitude of fluctuations in that trend (ΔT_Y) over the study period****.*

In the response letter, the authors note, “Given the limited importance of PC1_Env, any potential concerns regarding the biological interpretation of this axis should be considered of minor importance (R2e).” If this is the case, it calls into question for me the main framing focused on the role of environmental stochasticity, including: (a the title “plant population responses to environmental stochasticity are primarily driven by survival-reproduction trade-offs and mediated by aridity,” (b) the key take away “(i) Populations’ responses to environmental stochasticity can be linked to life-history strategies and

environmental stochasticity along a single axis of variation, from more buffered to less buffered" (lines 210-212), and (c) further discussion points such as "environmental stochasticity outweighs life history traits in predicting the buffering capacities of natural populations" (lines 224-225). Given the current manuscript framing, environmental stochasticity is represented by PCA1_Env. So, if this axis is of limited importance, this contradicts key takeaway points throughout the manuscript.

R3> Similarly to R2, the main point regarding the limited contribution of PC1_Env on $\sum E_v^\sigma$ should dissipate as the revised manuscript is now focused on the environmental variability, not the stochasticity per se. See, for example, the new lines summarising the main take-home messages.

> Lines 189-202: *Here, we provide a critical assessment of this knowledge gap and report several key findings: (i) Populations' responses to environmental variability can be represented along a single axis—from more buffered to less buffered; (ii) This axis reflects the combined influence of species' life-history (i.e., fast-slow and reproductive strategies), evolutionary history, and the environmental variability faced by each population; (iii) However, these relationships are complex, and considering phylogenetic relationships offers a different perspective. Notably, only the fast-slow continuum — but not the reproductive strategies continuum — remains an important predictor of populations' responses to environmental variability; (iv) Yet, the predictive power of the fast-slow continuum on the ability of a population to buffer against the environment is outweighed by variation in aridity; (v) And finally, whilst plants' populations responses to environmental variability are strongly determined by evolutionary history, contrary to predictions, the phylogenetic signal is stronger in reproduction (sexual and clonal) instead of the survival – the vital rate where temporal variance contributes most to population growth rate.*

The revision adds in a definition of environmental stochasticity equating temporal

variability with environmental stochasticity (lines 43-44). This is incorrect. Environmental stochasticity refers to random fluctuations in the environment, while temporal variability includes deterministic trends such as seasonality too – as the authors account for in their analyses. Having a precise definition is critical for the manuscript.

R4> We agree that we have been too permissive with the definition of environmental stochasticity. We have opted for a comprehensive and accurate definition of environmental stochasticity as requested by the reviewer. Please see the new lines 40-45.

> *Lines 40-45: Climate change projections anticipate a global increase in not only the mean of key abiotic drivers (e.g., temperature, precipitation), but also their temporal variability[1]. This variability encompasses both deterministic patterns (e.g., seasonality, long-term directional trends) and environmental stochasticity—the random, unpredictable fluctuations around those patterns[2,3]. Together, these sources of variation shape the environmental context in which populations persist and evolve, and both can strongly influence long-term population viability[4,5]. Consequently, climate change has already introduced significant eco-evolutionary challenges to the performance and survival of natural populations[5,6]. Indeed, climate change has been identified as a major driver of biodiversity loss worldwide[7]. As such, understanding whether and how the expected negative effects of environmental variability can be minimised has become a primary mission of Ecology, Evolution, and Conservation Biology[8].*

This lack of precision also occurs in the Results/Methods when discussing PCA2_Env. In the results, this axis is referred to as "temporal variation in aridity" (line 184), "stochasticity in aridity" (line 189), and just "aridity" (line 190). In the methods, it is stated that this axis is referred to as "seasonal aridity" (line 554) which is a shorter description of "relative amplitude of the trend of the precipitation" (line 554). While this could be a relatively minor fix in the main text, the comment in the response letter that PC1_Env is of "minor importance" made me wonder if the authors were also including PC2_Env as a measure of stochasticity, as stated on line 189. However, this axis has a very small loading from the stochasticity variable (R CV TempMax) according to Figure S4, so does not represent stochasticity.

R5> As previously mentioned (R4), we agree that we have been permissive with the definition of stochasticity. We also noted a confusion we have made regarding the word seasonal aridity. Instead of seasonal, the definition should be amplitude of the trend.

We have made a few amendments to the manuscript to make sure the axes of the environmental PCA now read accurately. We have reviewed all manuscript to standardise how we call PC1 and PC2. Please check lines 163-168 and lines 487-493 to see how the examples demonstrated by the reviewer have been addressed:

> Lines 163-168: *Indeed, in our 121 natural populations of plants,* ***temporal variation in aridity*** *($|\sum E_v^{\sigma}|$, $\beta\lambda_{PC2_{Env}}$ = 0.036 [0.008 – 0.060]), more so than in temperature ($\beta\lambda_{PC1_{Env}}$ = 0.017 [-0.017 – 0.052]; Figure 1), act as the main determinant of the placement of populations along the demographic buffering continuum. Moreover, the* ***effect of temporal variation in aridity*** *($\beta\lambda_{PC2_{Env}}$) outweighs over three-fold the estimated effect of the fast-slow continuum ($\beta\lambda_{PC1_{LH}}$) (posteriors $|\beta\lambda_{PC2_{Env}}/\beta\lambda_{PC1_{LH}}|$ = 0.036 /-0.017).*
>
> Lines 487-493 (definition of $PC2_{Env}$ is highlighted in bold): *A closer examination of $PC1_{Env}$ reveals that it decreases towards the tropics, mirroring the expected trend of higher $\overline{T}_{TempMax}$ and lower $R^{CV}_{TempMax}$ in tropical regions (Figure S5). In contrast, $PC2_{Env}$, which explains 20.59% of the environmental variation,* ***describes differences in the amplitude of the trend in precipitation, ΔT_{Prec}.*** *$PC2_{Env}$ is higher in more arid places such as deserts and the Mediterranean region. Therefore, we refer to these environmental axes as coupled gradient of mean and stochastic thermal conditions ($PC1_{Env}$) and aridity amplitude ($PC2_{Env}$).*

This new version also clearly stresses that $PC2_{Env}$ is better aligned with a deterministic component of the environmental variability. See lines 461-469.

Lines 461-469 (**highlighted in bold the differentiation between stochastic and deterministic components of environmental variability**): *[…] we retained only those climatic variables strictly related to changes in mean-variance environment and with direct biological or climate meaning, as these are the potential sources of variation in the vital rates. Thus, retained only the mean trend of the environmental conditions* ($\bar{T_Y}$)*, the amplitude of the trend component* (ΔT_Y)*, and the relative magnitude of the stochastic component within the environmental variability* (R_Y^{CV})*.* ***While*** $\boldsymbol{R_Y^{CV}}$ ***represents the monthly environmental uncertainty (i.e., the stochastic component of environmental variability), the other two variables (*** $\boldsymbol{T_Y}$ ***and*** $\boldsymbol{\Delta T_Y}$***) collectively capture both the mean magnitude of the trend component (*** $\boldsymbol{T_Y}$***) and the magnitude of fluctuations in that trend (*** $\boldsymbol{\Delta T_Y}$***) over the study period***.
